# Development and Validation of a Treatment Benefit Index to Identify Hospitalized Patients With COVID-19 Who May Benefit From Convalescent Plasma

**DOI:** 10.1001/jamanetworkopen.2021.47375

**Published:** 2022-01-25

**Authors:** Hyung Park, Thaddeus Tarpey, Mengling Liu, Keith Goldfeld, Yinxiang Wu, Danni Wu, Yi Li, Jinchun Zhang, Dipyaman Ganguly, Yogiraj Ray, Shekhar Ranjan Paul, Prasun Bhattacharya, Artur Belov, Yin Huang, Carlos Villa, Richard Forshee, Nicole C. Verdun, Hyun ah Yoon, Anup Agarwal, Ventura Alejandro Simonovich, Paula Scibona, Leandro Burgos Pratx, Waldo Belloso, Cristina Avendaño-Solá, Katharine J Bar, Rafael F. Duarte, Priscilla Y. Hsue, Anne F. Luetkemeyer, Geert Meyfroidt, André M. Nicola, Aparna Mukherjee, Mila B. Ortigoza, Liise-anne Pirofski, Bart J. A. Rijnders, Andrea Troxel, Elliott M. Antman, Eva Petkova

**Affiliations:** 1 Division of Biostatistics, Department of Population Health, New York University Grossman School of Medicine, New York; 2 Department of Environmental Medicine, New York University Grossman School of Medicine, New York; 3 Department of Biostatistics, School of Public Health, University of Washington, Seattle; 4 Biostatistics and Research Decision Sciences, Merck Research Labortory, Merck & Co Inc, Rahway, New Jersey; 5 Translational Research Unit of Excellence, Council Of Scientific And Industrial Research–Indian Institute of Chemical Biology, Kolkata, India; 6 Infectious Disease, Beleghata General Hospital, Kolkata, India; 7 School of Tropical Medicine, Kolkata, India; 8 Medical College Hospital, Kolkata, India; 9 Center for Biologics Evaluation and Research, Office of Biostatistics and Epidemiology, Analytics and Benefit-Risk Assessment Team, US Food and Drug Administration, Silver Spring, Maryland; 10 Office of Blood Research and Review, Center for Biologics Evaluation and Research, US Food and Drug Administration, Silver Spring, Maryland; 11 Albert Einstein College of Medicine and Montefiore Medical Center, Bronx, New York; 12 Indian Council of Medical Research, New Delhi, India; 13 Clinical Pharmacology Section, Department of Internal Medicine and Department of Research, Hospital Italiano de Buenos Aires, Buenos Aires, Argentina; 14 Clinical Pharmacology Section, Internal Medicine Service, Hospital Italiano de Buenos Aires, Buenos Aires, Argentina; 15 Transfusional Medicine Service, Hospital Italiano de Buenos Aires, Buenos Aires, Argentina; 16 Department of Research, Hospital Italiano de Buenos Aires, Buenos Aires, Argentina; 17 Hospital Universitario Puerta de Hierro Majadahonda, Madrid, Spain; 18 Department of Medicine, University of Pennsylvania Perelman School of Medicine, Philadelphia; 19 Zuckerberg San Francisco General, University of California, San Francisco; 20 Department of Intensive Care Medicine, University Hospitals Leuven, Leuven, Belgium; 21 Hospital Universitário de Brasília, University of Brasília, Brasília, Brazil; 22 Departments of Medicine and Microbiology, New York University Grossman School of Medicine, New York; 23 Department of Internal Medicine, Section of Infectious Diseases, Erasmus University Medical Center, Rotterdam, the Netherlands; 24 Brigham and Women’s Hospital, Harvard Medical School, Boston, Massachusetts; 25 Department of Child and Adolescent Psychiatry, New York University Grossman School of Medicine; 26 Nathan S. Kline Institute for Psychiatric Research, Orangeburg, New York

## Abstract

**Importance:**

Identifying which patients with COVID-19 are likely to benefit from COVID-19 convalescent plasma (CCP) treatment may have a large public health impact.

**Objective:**

To develop an index for predicting the expected relative treatment benefit from CCP compared with treatment without CCP for patients hospitalized for COVID-19 using patients’ baseline characteristics.

**Design, Setting, and Participants:**

This prognostic study used data from the COMPILE study, ie, a meta-analysis of pooled individual patient data from 8 randomized clinical trials (RCTs) evaluating CCP vs control in adults hospitalized for COVID-19 who were not receiving mechanical ventilation at randomization. A combination of baseline characteristics, termed the treatment benefit index (TBI), was developed based on 2287 patients in COMPILE using a proportional odds model, with baseline characteristics selected via cross-validation. The TBI was externally validated on 4 external data sets: the Expanded Access Program (1896 participants), a study conducted under Emergency Use Authorization (210 participants), and 2 RCTs (with 80 and 309 participants).

**Exposure:**

Receipt of CCP.

**Main Outcomes and Measures:**

World Health Organization (WHO) 11-point ordinal COVID-19 clinical status scale and 2 derivatives of it (ie, WHO score of 7-10, indicating mechanical ventilation to death, and WHO score of 10, indicating death) at day 14 and day 28 after randomization. Day 14 WHO 11-point ordinal scale was used as the primary outcome to develop the TBI.

**Results:**

A total of 2287 patients were included in the derivation cohort, with a mean (SD) age of 60.3 (15.2) years and 815 (35.6%) women. The TBI provided a continuous gradation of benefit, and, for clinical utility, it was operationalized into groups of expected large clinical benefit (B1; 629 participants in the derivation cohort [27.5%]), moderate benefit (B2; 953 [41.7%]), and potential harm or no benefit (B3; 705 [30.8%]). Patients with preexisting conditions (diabetes, cardiovascular and pulmonary diseases), with blood type A or AB, and at an early COVID-19 stage (low baseline WHO scores) were expected to benefit most, while those without preexisting conditions and at more advanced stages of COVID-19 could potentially be harmed. In the derivation cohort, odds ratios for worse outcome, where smaller odds ratios indicate larger benefit from CCP, were 0.69 (95% credible interval [CrI], 0.48-1.06) for B1, 0.82 (95% CrI, 0.61-1.11) for B2, and 1.58 (95% CrI, 1.14-2.17) for B3. Testing on 4 external datasets supported the validation of the derived TBIs.

**Conclusions and Relevance:**

The findings of this study suggest that the CCP TBI is a simple tool that can quantify the relative benefit from CCP treatment for an individual patient hospitalized with COVID-19 that can be used to guide treatment recommendations. The TBI precision medicine approach could be especially helpful in a pandemic.

## Key Points

**Question:**

What patient characteristics are associated with benefit from treatment with COVID-19 convalescent plasma (CCP)?

**Findings:**

This prognostic study of 2287 patients hospitalized with COVID-19 identified a combination of baseline characteristics that predict a gradation of benefit from CCP compared with treatment without CCP. Preexisting health conditions (diabetes, cardiovascular and pulmonary diseases), blood type A or AB, and earlier stage of COVID-19 were associated with a larger treatment benefit.

**Meaning:**

These findings suggest that simple patient information collected at hospitalization can be used to guide CCP treatment decisions for patients with COVID-19.

## Teaser

This prognostic study develops an index for predicting the expected relative COVID-19 convalescent plasma treatment benefit for patients hospitalized for COVID-19 using patients’ baseline characteristics.

## Introduction

Participants in randomized clinical trials (RCTs) typically exhibit heterogeneity of the treatment effect (HTE) of tested interventions. The traditional approach of focusing on the average effect has important limitations when making clinical decisions for individual patients.^1,2,3,4^ Precision medicine approaches have been developed to identify individual patients most likely to benefit from specific therapies.^5,6,7,8,9^

In this article, we report on an investigation to discover profiles of patients with COVID-19 associated with different benefit from COVID-19 convalescent plasma (CCP) treatment. The approach is based on a treatment benefit index (TBI), a continuous measure defined as a combination of patient characteristics that maximizes its interaction with CCP treatment.^10,11^ The TBI was derived using the COMPILE study^12^ and was validated in multiple external data sets.

The COMPILE study pooled individual patient data from 8 international RCTs and found no overall association between CCP and patient outcomes.^13^ The precision medicine investigations were prespecified in the COMPILE study’s statistical analysis plan.^14^

### HTE

Heterogeneity of CCP treatment benefit in COMPILE was observed with respect to (1) outcomes (clinical status based on the WHO ordinal scale^15^; binary outcomes of mechanical ventilation or death and all-cause mortality); (2) timing of assessments (day 14 vs 28); (3) quarter of enrollment (April to June 2020, July to September 2020, October to December 2020, and January to March 2021); and (4) patient demographic and clinical characteristics (eg, age, sex, comorbid medical conditions). While the first 3 factors correspond to nonpatient-related sources, factor 4 reflects patient-related heterogeneity that the TBI was constructed to identify. Quarter of enrollment, possibly through the evolving standard of care, might also affect the relative efficacy of CCP for patients with the same profile, but in different time periods; therefore, we also investigated the potential influence of quarter of enrollment on changes in the TBIs.

### TBI Objectives

The goal of this study was to guide CCP treatment recommendations by providing an estimate of a differential treatment outcome when a patient is treated with CCP vs without CCP. A larger differential in favor of CCP would indicate a more compelling reason for recommending CCP.

Two objectives were balanced: simplicity in terms of patient characteristics for implementation and accuracy in terms of benefit prediction for individual patients. Recognizing that not all patient information might be available when treatment decisions need to be made urgently, a complementary goal became the development of a basic TBI, on which improvements are possible with additional information. To demonstrate this idea, a basic TBI, using only easily obtained characteristics not including blood type, was derived, and then an expanded TBI, augmented with blood type information, was developed, improving the benefit prediction.

## Methods

### Data for TBI Development

COMPILE included 2369 hospitalized adults, not receiving mechanical ventilation at randomization, enrolled April 2020 to March 2021 (Table). COMPILE was approved by the New York University institutional review board, which determined that the study did not involve human participants because it used only deidentified data, thereby waiving the requirement for informed consent. This study followed the Transparent Reporting of a Multivariable Prediction Model for Individual Prognosis or Diagnosis (TRIPOD) reporting guideline. The control treatments varied across RCTs: standard of care (SOC), SOC plus saline, and SOC plus nonconvalescent plasma. While a few patients were not treated according to the randomization (due to administrative errors), the TBI derivation used treatment as randomized.

**Table.** Baseline Characteristics of Patients in the COMPILE Study by Benefit Level, Determined From the Expanded Treatment Benefit Index

Characteristic	Patients, No. (%)			
	Overall (N = 2287)	Benefit level B1 (n = 629)^a^	Benefit level B2 (n = 953)^a^	Benefit level B3 (n = 705)^a^
Age, mean (SD), y^b^	60.31 (15.2)	63.77 (13.8)	59.35 (15.5)	58.51 (15.5)
Sex				
Female	815 (36)	214 (26)	330 (41)	271 (33)
Male	1472 (64)	415 (28)	623 (42)	434 (30)
Baseline WHO score^b^				
4	447 (20)	214 (48)	233 (52)	0
5	1433 (63)	322 (22)	625 (44)	486 (34)
6	407 (18)	93 (23)	95 (23)	219 (54)
Blood type^b^				
O	1060 (46)	118 (11)	436 (41)	506 (48)
A	766 (34)	426 (56)	322 (42)	18 (2)
B	371 (16)	35 (9)	157 (42)	179 (48)
AB	90 (4)	50 (56)	38 (42)	2 (2)
Diabetes^b^				
Yes	768 (34)	309 (40)	307 (40)	152 (20)
Pulmonary disease^b^				
Yes	266 (12)	142 (53)	68 (26)	56 (21)
Cardiovascular disease^b^				
Yes	965 (42)	499 (52)	407 (42)	59 (6)
Enrollment quarter				
April to June 2020	619 (27)	176 (28)	291 (47)	152 (25)
July to September 2020	451 (20)	128 (28)	183 (41)	140 (31)
October to December 2020	867 (38)	236 (27)	355 (41)	276 (32)
January to March 2021	350 (15)	89 (25)	124 (35)	137 (39)
Days since symptoms				
0-3	279 (12)	87 (31)	103 (37)	89 (32)
4-6	812 (36)	228 (28)	332 (41)	252 (31)
7-10	816 (36)	224 (27)	346 (42)	246 (30)
11-14	253 (11)	66 (26)	115 (45)	72 (28)
>14	127 (6)	24 (19)	57 (45)	46 (36)

Abbreviation: WHO, World Health Organization.

^a^ B1 corresponds to expected large benefit; B2 corresponds to expected modest benefit; and B3 corresponds to expected potential harm or no benefit.

^b^ Baseline covariate included in the expanded treatment benefit index.

Six end points were available. There were 3 outcomes (ie, the ordinal WHO 11-point scale,^15^ a binary indicator of WHO score 7 to 10 [receiving mechanical ventilation or death], and binary indicator of WHO score 10 [mortality]) at 2 assessment times (ie, 14 ± 1 and 28 ± 2 days post randomization [hereafter day 14 and day 28]).^13^ The ordinal WHO scores and the indicators for mechanical ventilation or death at day 14 were coprimary outcomes in COMPILE. The TBIs were developed on the ordinal day-14 WHO score and then tested on all other outcomes.

### Statistical Analysis

#### Deriving the TBIs

The TBIs were developed using a single-index regression for estimating interactions between treatment and covariates,^11^ extended to accommodate ordinal outcomes.^16^ The TBIs are linear combinations of baseline characteristics constructed to optimally differentiate the association of CCP treatment with outcomes on day-14 WHO scores from that of control, using cumulative proportional odds models^16^ (POM; eAppendix 1 and eAppendix 2 in the Supplement). Candidate TBIs with different sets of baseline characteristics were identified from extensive internal cross-validation to optimize the generalizability of the TBIs (eFigure 1 in the Supplement), and the selected basic and expanded TBIs were tested using external data. Several forms of cross-validation were used: cross-validation from multiple random splits of the whole sample into training and testing sets (split-sample simulation; eAppendix 3 in the Supplement); cross-validation based on different RCTs (leave-one-RCT-out) to assess generalizability across RCTs (eAppendix 4 and eTable 1 in the Supplement); and cross-validation based on enrollment quarters (leave-one-enrollment-quarter-out) to assess stability of the performance over time (eAppendix 5 and eFigure 2 in the Supplement). Final sets of baseline characteristics were specified for the basic and expanded TBIs, and the associated POMs were reestimated from the whole data to give the final coefficients for the TBIs.

#### Utility Evaluation

In the derivation cohort, performance was measured by the CCP benefit in 2 subgroups identified from POM, one expected to benefit (B) and one not expected to benefit (NB) from CCP, in terms of their subgroup-specific odds ratios (ORs) and their ratios (ie, OR for B divided by OR for NB) to measure the difference between the CCP benefit in B vs NB. The cut point is where the CCP and the control curves crossed. An OR of less than 1 indicates CCP efficacy, with a smaller ratio of ORs indicating better TBI performance. An additional performance measure was the value, defined for the binary outcomes as the expected proportion of patients with the outcome if individuals in B are treated with CCP and those in NB are treated with the control; lower values were preferable since the outcomes are undesirable (eAppendix 2 in the Supplement).

The TBIs range from 0 to 1: larger values are associated with larger CCP benefit. For clinical utility, we operationalized the continuous TBIs as 3 benefit levels: large benefit (B1), modest benefit (B2), and no benefit or potential harm (B3) (eAppendix 6 in the Supplement). We evaluated the TBIs based on the within–benefit level CCP efficacy ORs. While the TBIs were derived on the ordinal WHO score at day 14, efficacy was assessed with respect to all 6 outcomes. ORs were obtained from models used in the main COMPILE analysis^13,14^: Bayesian POMs and logistic regressions, adjusted for the same covariates as in the COMPILE analysis with the same prior distributions. Bayesian posterior distributions of the respective ORs were obtained for each benefit level. Tests were 2-tailed. We contrasted the utility of the TBIs vs that of the individual baseline variables prespecified in COMPILE with outcomes to assess the advantage of the TBIs for guiding clinical decisions as an alternative to using individual baseline covariates. Finally, we contrasted the benefit levels with respect to the comparison of CCP vs control on time to all-cause mortality (log-rank test, stratified by RCT) and time to discharge within 28 days (Gray competing risk analysis^17^) to test for differences between the cumulative incidence functions.^18^

#### Alternative Development Methods

Fourteen alternative methods for developing treatment decision rules were used in search of a superior characterization of the HTE (eAppendix 7, eTable 2, and eTable 3 in the Supplement). However, none of them outperformed the TBI approach.

#### External Validation

Four CCP data sets (single-arm and RCTs) external to COMPILE were used to test the selected basic and expanded TBIs. The validation was based on the ordering of the ORs in the 3 benefit levels (ie, the B1 group having the smallest ORs and the B3 group having the largest would constitute validation). All data processing, analysis, and visualization were performed in R version 2021 (R Project for Statistical Computing). For frequentist inference, statistical significance was set at α = .05, and all tests were 2-tailed.

## Results

### TBIs Derivation

Baseline characteristics of the 2369 participants of the COMPILE study appear in eAppendix 8 and eTable 4 in the Supplement. The median (IQR) age was 60 (50-72) years, and 845 participants (35.7%) were women. For the day-14 ordinal WHO score, the posterior median CCP efficacy OR was 0.94 (95% credible interval [CrI], 0.74-1.19). Details appear in Troxel et al.^13^ The TBIs were developed on 2287 complete cases. The marginal and joint distributions of missing data appear in eFigure 3 in the Supplement. In the derivation cohort, the mean (SD) age was 60.3 (15.2) years, and 815 participants (35.6%) were women. The covariates’ main effect coefficients’ portion of model development, which is not a part of the TBI, appears in eAppendix 9 and eTable 5 in the Supplement. To identify variables included in the interaction parts of POM that define the TBIs, we considered 24 combinations of baseline characteristics (eAppendix 10 and eTable 6 in the Supplement). Results from split-sample cross-validation (eAppendix 11, eTable 7, eTable 8, and eFigure 4 in the Supplement), leave-one-RCT-out cross-validation (eAppendix 12, eTable 9, and eTable 10 in the Supplement), and leave-one-enrollment-quarter-out cross-validation (eAppendix 13 and eTables 11-13 in the Supplement) identified the basic and expanded TBIs. Additional results from the split-sample cross-validation investigations appear in eAppendix 14 and eFigures 5 to 10 in the Supplement.

eAppendix 15 and eTable 14 in the Supplement report the coefficients and 95% bootstrap CIs of the linear combinations that define the basic and expanded TBIs. The improvement in the benefit prediction by TBI because of the inclusion of information on blood type can be assessed by comparing the basic and expanded TBIs with respect to the OR ratios (eTables 7, 9, 11, and 12 in the Supplement) and value (eTables 8, 10, and 13 in the Supplement). Additional comparison based on cross-classification^19^ appears in eAppendix 16 and eTable 15 in the Supplement. The proportional odds assumption for POM was assessed to be reasonable (eAppendix 17 and eTables 16 and 17 in the Supplement). Additional information on the fitted POM is given in eAppendices 30 and 31 and eFigures 28 to 33 in the Supplement.

### Major Findings From the Internal Cross-Validation

The TBIs were assessed through leave-one-RCT-out cross-validation (eTable 1 in the Supplement for the 8 RCTs), which provides support for their generalizability (eTables 9 and 10 in the Supplement). The TBIs’ performance in the leave-one-enrollment-quarter-out cross-validation (eTables 11-13 in the Supplement) indicated that although the efficacy of SOC changed over time, the relative benefit from CCP was determined by the same combination of patient characteristics.

### Internal Evaluation of CCP TBI

**Figure 1.**
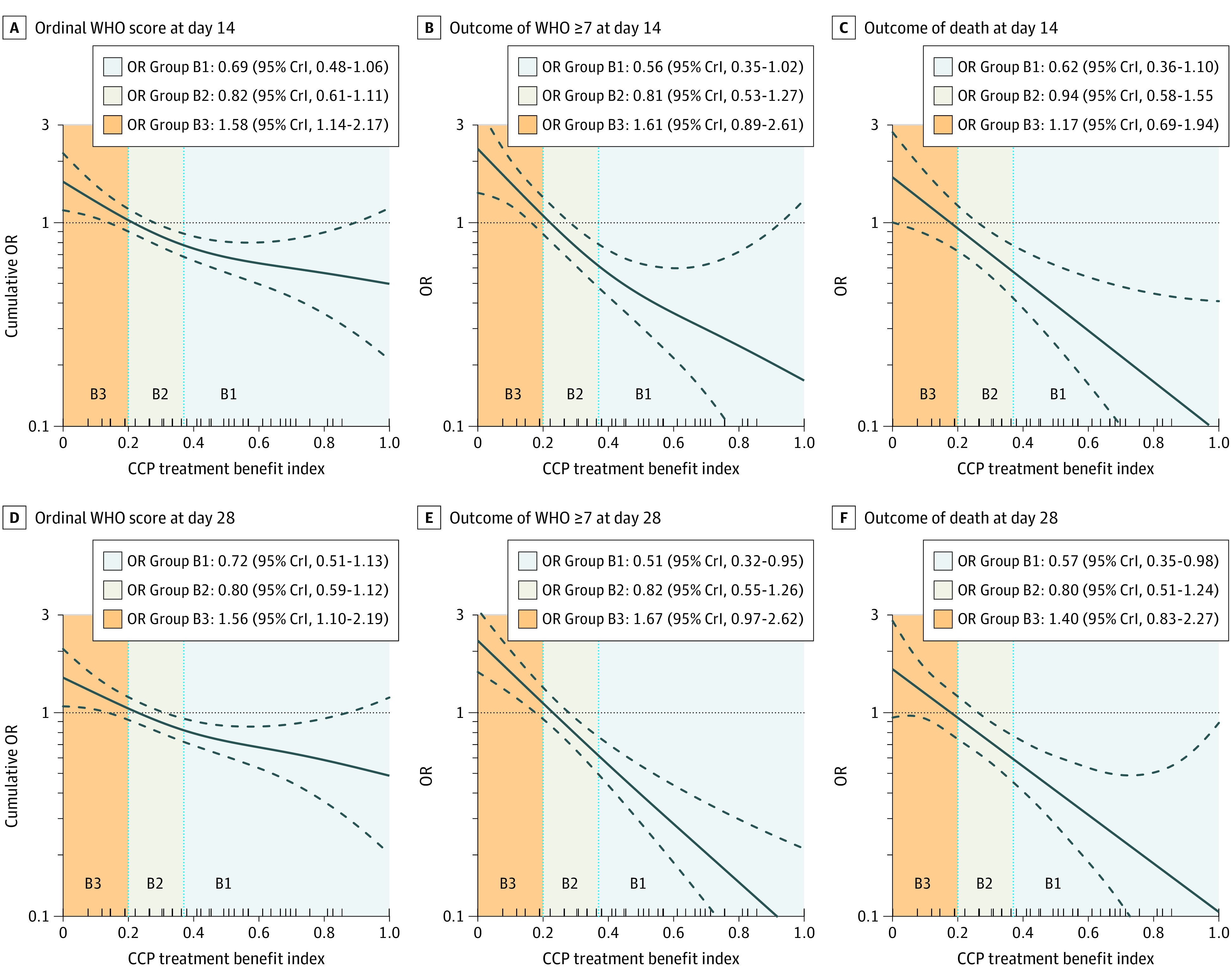
Odds Ratios of COVID-19 Convalescent Plasma (CCP) Efficacy and Expanded Treatment Benefit Index For all 6 outcomes, odds ratios of CCP efficacy (vs control) are shown as a function of the expanded treatment benefit index developed on the outcome of day-14 ordinal World Health Organization (WHO) scale. The plotted odds ratios (ORs) were estimated from cumulative proportional odds models or logistic models, depending on the outcome. The regressors were treatment, spline-represented treatment benefit index, and spline-represented treatment benefit index × treatment interaction, not adjusted for any other covariates. ORs for CCP efficacy of less than 1 indicate better outcome with CCP treatment than control. The cut points distinguishing benefit levels B1, B2, and B3 were 0.20 and 0.37 and are the same for all panels. The solid curves represent the ORs from the model, and the dashed curves indicate the associated 95% bootstrap confidence bands. The ORs for the 3 benefit levels are estimated from the primary bayesian models used in the analysis of the main results.

Figure 1 shows the expanded TBI (developed on day-14 ordinal WHO scores) plotted against the unadjusted ORs for CCP efficacy for all 6 outcomes. All panels show a monotonically decreasing trend of the ORs (indicating an increase in the CCP benefit) as the TBI score increases from 0 to 1. Some of the OR curves and the 95% confidence bands exceeded 1 for very small TBI values, suggesting the possibility of harm from CCP as TBI approaches 0.

The dotted vertical lines in Figure 1 mark the groupings corresponding to large benefit (B1), modest benefit (B2), and no benefit/potential harm (B3) groups that operationalize the TBI for clinical utility. The benefit levels were determined by the day-14 ordinal WHO score, and the cut points were chosen to optimize the 3-category model fit as described in eAppendix 6 in the Supplement. eAppendix 18, eFigures 11 and 12, and eTables 18 and 19 in the Supplement contain more details on this categorization and the internal cross-validation results. The in-sample proportion of patients in these 3 groups were as follows: 629 participants (27.5%) in B1; 953 participants (41.7%), B2; and 705 (30.8%), B3. These same cut points and benefit levels are used for all panels of Figure 1 and in all analyses that follow.

The Table describes the distribution of patient characteristics by the 3 benefit levels (eAppendix 19, eTables 20 and 21, and eFigure 13 in the Supplement). The benefit level–specific CCP efficacy ORs; the posterior probability of an OR of less than 1, indicating the evidence of any CCP benefit; and the posterior probability of an OR of less than 0.80, indicating probability of more than minimal CCP benefit for all 6 outcomes appear in eAppendix 20, eTable 22, and eFigure 14 in the Supplement. With respect to day-14 ordinal WHO scores, the B1 group had a posterior median OR of 0.69 (95% CrI, 0.48-1.06), a posterior probability of any CCP benefit of 96%, and the posterior probability of more than minimal benefit of 77%, indicating strong evidence for benefit from CCP; for B2, the posterior median OR was 0.82 (95% CrI, 0.61-1.11), and posterior probabilities of any benefit of 90% and more than minimal benefit of 42%, indicating modest evidence for CCP efficacy; and for B3, the posterior median OR of 1.58 (95% CrI, 1.14-2.17) and zero posterior probabilities for any CCP benefit and more than minimal benefit, indicating strong evidence for no efficacy and potential harm. For all outcomes, patients in B1 were expected to have large benefit, patients in B2 expected to have modest benefit, and patients in B3 expected to have at least a potential for harm from CCP. Additionally, eAppendix 21 and eFigures 15 to 20 in the Supplement include forest plots of ORs for subgroups defined by individual patient characteristics for the 6 outcomes. The benefit levels defined by the TBI show stronger separation of ORs across all outcomes than any individual covariate.

**Figure 2.**
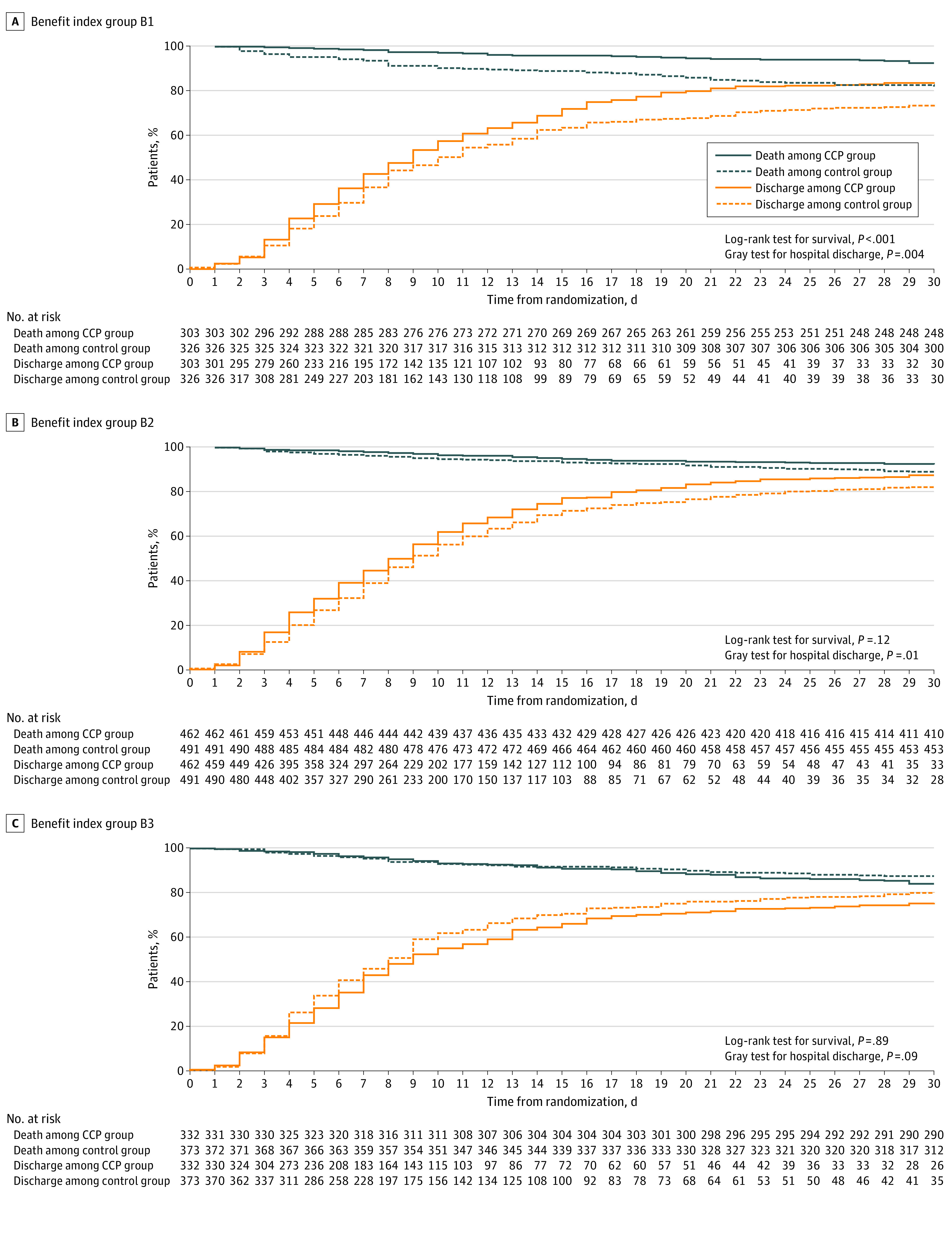
Time to Death and Discharge Within 28 Days in 3 Benefit Level Groups Log-rank tests stratified for randomized clinical trials were used to compare COVID-19 Convalescent Plasma (CCP) and control for the mortality outcome. Gray competing risk test was used to compare CCP and control for the discharge outcome.

Figure 2 summarizes results for time to death and time to hospital discharge (up to day 28). There was a large benefit with respect to mortality in B1, modest benefit in B2, and no benefit in B3. With respect to time to discharge, patients in B1 were discharged 2.3 (95% CI, 0.7-3.8) days earlier if treated with CCP compared with control treatment. Results for the basic TBI were similar (eAppendices 22-25, eFigures 21-24, and eTables 23-25 in the Supplement).

**Figure 3.**
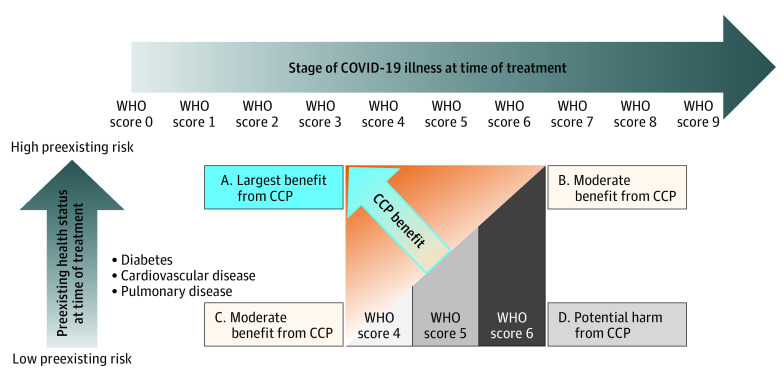
Preexisting Health Status, Stage of COVID-19 Illness at Time of Treatment, and Benefit From COVID-19 Convalescent Plasma (CCP) Patients in the upper left corner (A), who have high preexisting risk but are at an early stage of COVID-19, are expected to have large benefit from CCP treatment. Patients with high preexisting risk who are at an advanced stage of COVID-19 (upper-right corner; B) as well as patients with low preexisting risk who are at early stage of COVID-19 (lower-left corner; C) are expected to benefit less from CCP. Patients with low preexisting risk who are at an advanced stage of COVID-19 (lower-right corner; D) are not expected to benefit and might experience harm from CCP treatment. WHO indicates World Health Organization.

**Figure 4.**
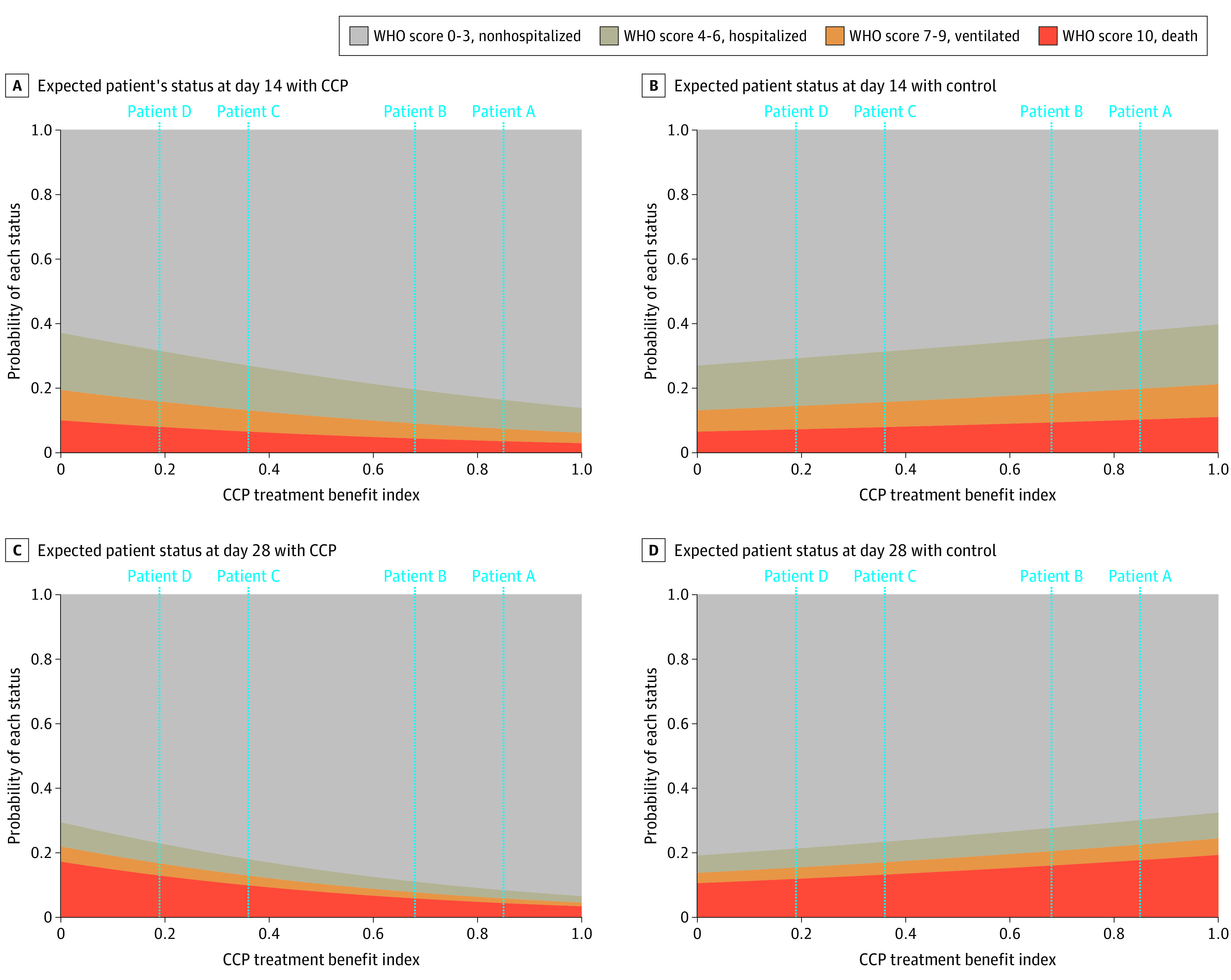
Predicted Patient Status for 4 Sample Patients All 4 hypothetical patients were aged 60 years and had blood type O. Patient A had high preexisting risk (ie, cardiovascular disease, diabetes, and pulmonary disease) and early-stage COVID, with a treatment benefit index score of 0.85 (benefit level B1); patient B, high preexisting risk and later-stage COVID-19, with a treatment benefit index score of 0.68 (benefit level B1); patient C, low preexisting risk and early-stage COVID-19, with a treatment benefit index score of 0.36 (benefit level B2); and patient D, low preexisting risk and late-stage COVID-19, with a treatment benefit score of 0.19 (benefit level B3). CCP indicates COVID-19 convalescent plasma; WHO, World Health Organization.

Figure 3 provides a visual interpretation of the TBI. The vertical axis corresponds to preexisting health risk and the horizontal axis corresponds to the stage of COVID-19 at time of treatment. CCP benefit depends on both: CCP is most associated with benefit for patients with high preexisting risk who have early-stage COVID-19 at time of treatment (ie, patients in the upper-left corner of the figure), and it is least associated with benefit—and potentially associated with harm—for patients with low preexisting risk and an advanced stage of COVID-19. Figure 4 shows 4 hypothetical patients with different preexisting health risks and stages of the disease at time of treatment; these patients roughly correspond to the 4 corners of the rectangle in Figure 3, ie, patient A has early-stage COVID-19 (WHO score 4) and high preexisting risk; patient B, later-stage COVID-19 (WHO score 6) and high preexisting risk; patient C, early-stage COVID-19 (WHO score 4) and low preexisting risk; and patient D, later-stage (WHO score 6) and low preexisting risk. The probabilities of these patients’ expected WHO scores on days 14 and 28 appear in the top and bottom panels of Figure 4, respectively. The recommendation for patients A, B, and C is treatment with CCP, with the most substantial benefit compared with control expected for patient A (TBI score, 0.85), followed by patients B (TBI score, 0.68) and C (TBI score, 0.36). For patient D (TBI score, 0.19), CCP treatment is not recommended given that this patient has a benefit level of B3.

### External Validation

#### Expanded Access Program Study

Early in the pandemic in the United States, a single-arm expanded access program (EAP) sponsored by the Mayo Clinic was established to provide access to CCP for hospitalized patients with COVID-19.^20,21^ Mortality on day 28 was the outcome. Of those who received CCP, 8698 had all baseline characteristics for computing the TBIs. Given that no participants in a control group were available from the EAP study, an EAP sample was matched to the COMPILE control participants enrolled during the concurrent time (April to September 2020), using exact matching on categorical variables and coarsened exact matching on age, yielding 1896 patients receiving CCP and 212 patients not receiving CCP. The expanded and basic TBIs were computed for these patients, and they were stratified into the predefined B1, B2 and B3 benefit levels. The ORs for the expanded TBI were as follows: for the B1 group, 0.41 (95% CI, 0.24-0.71); B2 group, 0.71 (95% CI, 0.45-1.12); and B3 group, 1.09 (95% CI, 0.65-1.81), supporting validation of the TBI (eAppendix 26 and eTables 26-30 in the Supplement).

#### Emergency Use Authorization Study

Under Emergency Use Authorization (EUA), CCP was permitted outside of clinical studies^22^ in the United States (eAppendix 27 in the Supplement). Overall, 216 hospitalized participants (210 of whom were not receiving mechanical ventilation at time of treatment) were treated with CCP (HA Yoon, email and telephone, March 13 to April 20, 2021). The outcome was day-14 ordinal WHO scores. Patients receiving CCP were matched on age, sex, and baseline WHO status with COMPILE control participants enrolled in concurrent times (October 2020 to March 2021), resulting in a matched set of 210 patients receiving CCP and 210 control patients. The ORs of the expanded TBI satisfied the conditions for validation and were as follows: for the B1 group, 0.91 (95% CI, 0.50-1.65); B2, 1.17 (95% CI, 0.67, 2.04); and B3, 3.00 (95% CI, 1.64-5.46) (eAppendix 27 and eTables 31-34 in the Supplement).

#### First RCT Not in COMPILE

Data from an RCT^23^ external to COMPILE comparing CCP vs SOC in hospitalized adults not receiving mechanical ventilation at randomization was provided for testing the TBIs. A total of 80 patients were randomized 1:1 to CCP and SOC (eAppendix 20 in the Supplement). The study found no significant CCP treatment effect.^23^ The outcome was 30-day mortality. Blood type information was only available for patients randomized to CCP, and therefore, only the basic TBI was used for the validation. The ORs for the predefined benefit levels indicated validation of the TBI: for B1 group, 0.31 (95% CI, 0.02-4.41); for B2 group, 0.69 (95% CI, 0.21-2.30); and for B3 group, 0.80 (95% CI, 0.09-6.85) (eAppendix 28, eTables 35-38, and eFigure 25 in the Supplement).

#### Second RCT Not in COMPILE

A total of 333 patients in an RCT were randomized to 2:1 to CCP and saline.^24^ The outcome was a 6-point version of the ordinal WHO scale. Overall, 309 patients had all baseline covariates for computing the TBIs. Both the basic and the expanded TBIs could be tested, and both were validated. With respect to day-14 WHO 6-point ordinal outcome, the ORs for the expanded TBI were as follows: for the B1 group, 0.44 (95% CI, 0.12-1.65); B2, 0.99 (95% CI, 0.49, 2.01), and B3, 1.04 (95% CI, 0.55-1.96) (eAppendix 29, eTables 39-42, and eFigures 26 and 27 in the Supplement).

## Discussion

The TBIs reported in this study consist of simple combinations of baseline patient characteristics. While continuous, the TBIs can be operationalized as discrete benefit levels for the utility of making clinical decisions about treating patients with COVID-19 with CCP. Within the B1 group, patients with TBI scores approaching 1 were expected to experience large, clinically meaningful benefits from CCP. Within the B3 group, patients with TBIs approaching 0 were expected to experience harm, while the rest of the patients in this group would likely experience no benefit. Patients in the B2 group were expected to experience modest benefit.

The proposed TBIs were validated on 4 external data sets, providing evidence of its generalizability outside COMPILE and utility in practice. While the prevalence of COMPILE individuals in the benefit level groups (ie, B1, B2, and B3) was 28%, 42% and 31% respectively, the general prevalence would depend on the composition of the COVID-19 hospitalized population in different regions of the world at different times. The prevalence of patients in COMPILE expected to benefit most from CCP (B1 group) decreased, while the prevalence of patients expected to have potential harm (B3 group) increased over the enrollment quarters (Table). This may, at least partly, explain the observed decreasing trend over time in CCP efficacy (eAppendix 5 and eFigure 2 in the Supplement).

The basic TBI can be augmented with additional pretreatment characteristics to make further refinements of the clinical recommendations. This feature can be particularly useful when data on patients’ pretreatment antibody levels or other laboratory values are available. We found that no individual characteristic alone was as effective as the TBI in characterizing HTE.

### Limitations

This study has limitations. As COVID-19 continues to evolve through mutations, the TBIs developed from the COMPILE study may need to be updated to reflect these potential changes. Just as current COVID-19 vaccines may lose their effectiveness as the virus mutates,^25^ the optimal TBI composition might also change. Additionally, this study did not discuss the association of antibody levels in the donors’ plasma with CCP efficacy. However, the method for deriving the TBI can be extended by allowing for a continuous measure of treatment^16^ (eg, titer quantities in donors’ plasma).

## Conclusions

The TBI presented in this study is a simple tool that provides predictions for individual patients regarding their relative benefit from treatment with vs without CCP. The proposed TBIs are implemented as an application available for desktops and mobile devices.^26^

## Notes

Supplement. **eAppendix 1.** TBI Models and Abbreviations **eAppendix 2.** Workflow and Internal Cross-validation **eFigure 1.** Workflow Diagram for Developing the TBI **eAppendix 3.** Split-Sample-Simulation Cross-validation, 1000 replications **eAppendix 4.** Leave-One-RCT-Out Cross-validation **eTable 1. **RCTs Participating in COMPILE **eAppendix 5.** Leave-One-Enrollment-Quarter-Out Cross-validation **eFigure 2.** Median ORs and 95% Credible Intervals From the Posterior Probability Distributions for the ORs, by Patient Enrollment Quarters **eAppendix 6.** Determination of 3 Levels of Benefit **eAppendix 7.** Application of Other Methods for Treatment Classification Rules **eTable 2.** Results in Terms of Odds Ratios from Cross-validation Based on 400 Splits Into Training Data Set and Validation Data Set **eTable 3.** Results in Terms of Values From Cross-validation Based on 400 Splits Into Training Data Set and Validation Data Set **eAppendix 8. **Baseline Characteristics of the Participants in the Study Used to Develop the TBIs **eTable 4. **Baseline Characteristics of the Participants in the COMPILE Study by Treatments **eFigure 3.** Missingness for Each of the Key Baseline Characteristics and Outcomes and Each Combination of the Variables for the 82 Participants Dropped From the Complete Case Analysis **eAppendix 9.** Main Effect Model **eTable 5.** Main Effects Model **eAppendix 10.** Patient Features Considered for Inclusion in the TBI **eTable 6.** Candidate Sets of Features Considered for Inclusion in the TBI **eAppendix 11.** Results From 1000 Split-Sample-Simulation Cross-validation **eTable 7.** Results From 1000 Split-Sample Simulation Cross-validation **eFigure 4.** Subgroup-Specific OR Distributions Obtained From a 1000 Split-Sample Simulation Cross-validation **eTable 8.** Value Results From the 1000 Split-Sample Simulation Cross-validation **eAppendix 12.** Results From Leave-One-RCT-Out Cross-validation **eTable 9.** Results From a Leave-One-RCT-Out Cross-validation **eTable 10.** Value Results From a Leave-One-RCT-Out Cross-validation **eAppendix 13.** Results From Leave-One-Enrollment-Quarter-Out Cross-validation **eTable 11.** Results From a Leave-One-Enrollment-Quarter-Out Cross-validation **eTable 12.** Results From a Leave-One-Enrollment-Quarter-Out Cross-validation, Before Averaging Across the 4 Enrollment Quarters (Q2-Q5) for the Basic and Expanded TBIs **eTable 13. **Value Results From a Leave-One-Enrollment-Quarter-Out Cross-validation **eAppendix 14.** Comparisons With a Naive Treatment Decision Approach **eFigure 5.** Distributions of Subgroup-Specific ORs Obtained From 1000 Randomly Split Testing Sets for the Ordinal Outcome WHO Score at Day 14 **eFigure 6. **Distributions of Subgroup-Specific ORs Obtained From 1000 Randomly Split Testing Sets for the Ordinal Outcome WHO Score at Day 28 **eFigure 7. **Distributions of Subgroup-Specific ORs Obtained From 1000 Randomly Split Testing Sets for Binary Outcome of WHO Score of at Least 7 at day 14 **eFigure 8. **Distributions of Subgroup-Specific ORs Obtained From 1000 Randomly Split Testing Sets for Binary Outcome of WHO Score of at Least 7 at Day 28 **eFigure 9.** Distributions of Subgroup-Specific ORs Obtained From 1000 Randomly Split Testing Sets for Death at Day 14 **eFigure 10. **Distributions of Subgroup-Specific ORs Obtained From 1000 Randomly Split Testing Sets for Death at Day 28 **eAppendix 15.** Specification of the Basic and Expanded TBIs **eTable 14. **Coefficients of the Linear Combination for Baseline Patient Characteristics in the Basic and Expanded TBIs **eAppendix 16.** Cross-Stratification Table Comparing the Basic and Expanded TBIs **eTable 15. **Benefit Stratification Table **eAppendix 17.** Proportional Odds Assumption Test **eTable 16. **Estimates of Coefficients Associated With HTE **eTable 17. **Brant Test Results **eAppendix 18.** Three Levels of Benefit for the Basic and Expanded TBIs **eFigure 11.** Basic TBI Model-Based Cumulative Odds Ratio for the Ordinal WHO Score at Day 14, as a Function of the Basic TBI **eFigure 12.** Expanded TBI Model-Based Cumulative Odds Ratio for the Ordinal WHO Score at Day 14, as a Function of the Expanded TBI **eTable 18. **Odds Ratios Associated with All 6 Outcomes, Under 3 Levels of Categorization From the Basic and Expanded TBIs **eTable 19. **Results Regarding the Expected Incidence (Value) of the 4 Clinically Undesirable Binary Outcomes When CCP is Administered to the 3 Patient Subgroups Identified by the Basic and Expanded TBIs **eAppendix 19.** Baseline Patient Characteristics by Benefit Levels **eTable 20.** Baseline Characteristics of COMPILE Patients in the 3 Benefit-Level Groups Defined From the Expanded TBI **eFigure 13.** Proportions of the 3 Benefit-Level Groups Identified From the Expanded TBI, by Quarter **eTable 21.** Baseline Patient Characteristics for the Subgroup of Patients Who Would and Would Not Be Advised to Receive CCP, Determined by the Expanded TBI **eAppendix 20. **CCP Efficacy in the 3 Benefit Groups With Respect to All 6 Outcomes, Determined by the Expanded TBI **eTable 22.** Posterior Probability Distributions of the ORs of CCP vs Control for All Outcomes, by Benefit Group Determined From the Expanded TBI **eFigure 14.** Forest Plots of Median ORs and 95% Credible Intervals From the Posterior Probability Distributions for the ORs by Benefit Groups Determined From the Expanded TBI for All 6 Outcomes **eAppendix 21.** Differential Treatment Effect by Benefit Levels, in Comparison With Differential Treatment Effect by Single Patient Characteristics **eFigure 15. **Odds Ratios and 95% Credible Intervals for Subgroup-Specific Odds for Individual Baseline Covariates and 3 Benefit Groups for WHO Score on Day 14 **eFigure 16. **Odds Ratios and 95% Credible Intervals for Subgroup-Specific Odds for Individual Baseline Covariates and 3 Benefit Groups for WHO Score 7 to 10 on Day 14 **eFigure 17. **Odds Ratios and 95% Credible Intervals for Subgroup-Specific Odds for Individual Baseline Covariates and 3 Benefit Groups for WHO Score on Day 28 **eFigure 18. **Odds Ratios and 95% Credible Intervals for Subgroup-Specific Odds for Individual Baseline Covariates and 3 Benefit Groups for WHO Score 7 to 10 on Day 28 **eFigure 19. **Odds Ratios and 95% Credible Intervals for Subgroup-Specific Odds for Individual Baseline Covariates and 3 Benefit Groups for Mortality on Day 14 **eFigure 20. **Odds Ratios and 95% Credible Intervals for Subgroup-Specific Odds for Individual Baseline Covariates and 3 Benefit Groups for Mortality on Day 28 **eAppendix 22.** CCP Benefit as a Function of the Basic TBI **eFigure 21. **CCP Benefit as a Function of the Basic TBI **eAppendix 23.** Baseline Patient Characteristics by Benefit Levels Determined From the Basic TBI **eTable 23.** Baseline Characteristics of COMPILE Patients in the 3 Benefit-Level Groups Defined by the Basic TBI **eFigure 22.** Proportions of the 3 Benefit Groups (B1, B2 and B3) Identified From the Basic TBI, by Quarter **eTable 24.** Baseline Patient Characteristics for the Subgroup of Patients Who Would and Would Not Be Advised to Receive CCP, Determined by the Basic TBI **eAppendix 24. **CCP Efficacy in the 3 Benefit Groups With Respect to All 6 Outcomes, Determined by the Basic TBI **eTable 25.** Posterior Probability Distributions of the Odds Ratios of CCP vs Control for All Outcomes, by Benefit Group Determined From the Basic TBI **eFigure 23.** Forest Plots of Median Odds Ratios and 95% Credible Intervals From the Posterior Probability Distributions for the Odds Ratios by Benefit Groups Determined From the Basic TBI for All 6 Outcomes **eAppendix 25.** Kaplan-Meier Plots for Time to Death and Time to Hospital Discharge, With Log-Rank Test for Time to Death and Gray Competing Risk Test for Time to Hospital Discharge, Basic TBI **eFigure 24.** Comparison Between CCP and Control Patients for Time to Death and Time to Hospital Discharge by Benefit Level, Determined by the Basic TBI **eAppendix 26. **Validation on Expanded Access Program (EAP) Data **eTable 26.** Baseline Characteristics for Participants Treated Under the EAP With Available Data for All Specified Parameters **eTable 27.** Summary of Balance for Matched Data for EAP **eTable 28.** Summary of Balance for Matched Data for EAP, High Titer Only **eTable 29.** Participants in the Matched Data, by Benefit Levels Defined by the Expanded and the Basic TBIs and Depending on Whether All EAP Patients or Only Those Treated With High-Titer Plasma Were Used For Validation **eTable 30.** CCP Benefit Odds Ratios Within Different Benefit Levels Defined by the Basic and the Expanded TBIs and Depending on Whether Subgrouping Was Restricted to Patients with High-Titer CCP **eAppendix 27. **Validation on an Emergency Use Authorization (EUA) Study **eTable 31.** Baseline Characteristics of CCP Recipients at Montefiore Medical Center, Bronx, NY **eTable 32.** Summary of Balance for Matched Data for the EUA **eTable 33.** Patients in Different Benefit Levels defined by the Basic TBI and the Expanded TBI for the EUA **eTable 34. **Subgroup-Specific CCP Benefit Odds Ratios for Benefit Levels Defined by the Basic TBI and the Expanded TBI for the EUA **eAppendix 28.** Validation on External RCT 1 **eTable 35.** Baseline Patient Characteristics and Clinical Information of the First RCT **eTable 36.** Major Pharmacotherapy as the Standard of Care **eTable 37.** Patients in Different Benefit Levels Based on the Basic TBI in the First RCT **eTable 38. **Subgroup-Specific CCP Benefit Odds Ratios With Respect to 30-Day Mortality, by Benefit Level Defined by the Basic TBI, in the First RCT **eFigure 25.** CCP vs Control as a Function of the Basic TBI (Which Does Not Require Blood Type Information), With the 3 Benefit Groups Overlaid, Evaluated on the First External RCT **eAppendix 29.** Validation on External RCT 2 **eTable 39.** Baseline Patient Characteristics for the Second RCT **eTable 40.** Patients in Different Benefit Levels Based on the Expanded and Basic TBIs for the Second RCT **eTable 41.** CCP Benefit Odds Ratios for Ordinal Outcomes at Days 14 and 30 for the Benefit Groups Defined by the Basic and Expanded TBIs, Evaluated on the Second External RCT **eFigure 26. **CCP Benefit Odds Ratios for WHO 6-Point Scale Ordinal Outcomes at Days 14 and 30, as Functions of the Basic and Expanded TBIs, Evaluated on the Second External RCT **eTable 42.** CCP Benefit Odds Ratios for the Binary Outcome of Ventilation or Worse at Days 14 and 30 for Benefit Groups Defined by the Basic and Expanded TBIs, Evaluated on the Second External RCT **eFigure 27. **CCP Benefit Odds Ratios for Binary Outcome of Ventilation or Worse at Days 14 and 30 for Benefit Groups Defined by the Basic and Expanded TBIs, Evaluated on the Second External RCT **eAppendix 30. **More Results From the Expanded TBI **eFigure 28.** Probability of the 11-Point WHO Ordinal Scales Categorized into 4 Ordinal Categories, as a Function of the Expanded TBI **eAppendix 31.** Response Surface and Scatterplots Associated With the Expanded TBI Model **eFigure 29.** Fitted POM **eFigure 30.** Fitted POM Displayed on the Probability Scale **eFigure 31.** Scatterplot of the Risk Index and the TBI in the Fitted POM **eFigure 32.** Scatterplot of the Risk Index and the TBI-by-Treatment Interaction Term in the Fitted POM **eFigure 33. **Empirical Cumulative Distribution Functions for the Basic and Expanded TBIs With the Associated Benefit Levels Overlaid
