# Supplementary material for: Development and Validation of a Treatment Benefit Index to Identify Hospitalized Patients With COVID-19 Who May Benefit From Convalescent Plasma
Source: JAMA Netw Open. 2022 Jan 25;5(1):e2147375. doi: 10.1001/jamanetworkopen.2021.47375 (PMC8790670; doi:10.1001/jamanetworkopen.2021.47375)
Supplement: Supplement. — eAppendix 1. TBI Models and Abbreviations eAppendix 2. Workflow and Internal Cross-validation eFigure 1. Workflow Diagram for Developing the TBI eAppendix 3. Split-Sample-Simulation Cross-validation, 1000 replications eAppendix 4. Leave-One-RCT-Out Cross-validation eTable 1. RCTs Participating in COMPILE eAppendix 5. Leave-One-Enrollment-Quarter-Out Cross-validation eFigure 2. Median ORs and 95% Credible Intervals From the Posterior Probability Distributions for the ORs, by Patient Enrollment Quarters eAppendix 6. Determination of 3 Levels of Benefit eAppendix 7. Application of Other Methods for Treatment Classification Rules eTable 2. Results in Terms of Odds Ratios from Cross-validation Based on 400 Splits Into Training Data Set and Validation Data Set eTable 3. Results in Terms of Values From Cross-validation Based on 400 Splits Into Training Data Set and Validation Data Set eAppendix 8. Baseline Characteristics of the Participants in the Study Used to Develop the TBIs eTable 4. Baseline Characteristics of the Participants in the COMPILE Study by Treatments eFigure 3. Missingness for Each of the Key Baseline Characteristics and Outcomes and Each Combination of the Variables for the 82 Participants Dropped From the Complete Case Analysis eAppendix 9. Main Effect Model eTable 5. Main Effects Model eAppendix 10. Patient Features Considered for Inclusion in the TBI eTable 6. Candidate Sets of Features Considered for Inclusion in the TBI eAppendix 11. Results From 1000 Split-Sample-Simulation Cross-validation eTable 7. Results From 1000 Split-Sample Simulation Cross-validation eFigure 4. Subgroup-Specific OR Distributions Obtained From a 1000 Split-Sample Simulation Cross-validation eTable 8. Value Results From the 1000 Split-Sample Simulation Cross-validation eAppendix 12. Results From Leave-One-RCT-Out Cross-validation eTable 9. Results From a Leave-One-RCT-Out Cross-validation eTable 10. Value Results From a Leave-One-RCT-Out Cross-validation eAppendix 13. Results [file jamanetwopen-e2147375-s001.pdf]

# Supplemental Online Content

Park H, Tarpey T, Liu M, et al. Development and validation of a treatment benefit index to identify hospitalized patients with COVID-19 who may benefit from convalescent plasma. *JAMA Netw Open.* 2022;5(1):e2147375. doi:10.1001/jamanetworkopen.2021.47375

**eAppendix 1.** TBI Models and Abbreviations

**eAppendix 2.** Workflow and Internal Cross-validation

**eFigure 1.** Workflow Diagram for Developing the TBI

**eAppendix 3.** Split-Sample-Simulation Cross-validation, 1000 replications

**eAppendix 4.** Leave-One-RCT-Out Cross-validation

**eTable 1.** RCTs Participating in COMPILE

**eAppendix 5.** Leave-One-Enrollment-Quarter-Out Cross-validation

**eFigure 2.** Median ORs and 95% Credible Intervals From the Posterior Probability Distributions for the ORs, by Patient Enrollment Quarters

**eAppendix 6.** Determination of 3 Levels of Benefit

**eAppendix 7.** Application of Other Methods for Treatment Classification Rules

**eTable 2.** Results in Terms of Odds Ratios From Cross-validation Based on 400 Splits Into Training Data Set and Validation Data Set

**eTable 3.** Results in Terms of Value From Cross-validation Based on 400 Splits Into Training Data Set and Validation Data Set

**eAppendix 8.** Baseline Characteristics of the Participants in the Study Used to Develop the TBIs

**eTable 4.** Baseline Characteristics of the Participants in the COMPILE Study by Treatments

**eFigure 3.** Missingness for Each of the Key Baseline Characteristics and Outcomes and Each Combination of the Variables for the 82 Participants Dropped From the Complete Case Analysis

**eAppendix 9.** Main Effect Model

**eTable 5.** Main Effects Model

**eAppendix 10.** Patient Features Considered for Inclusion in the TBI

**eTable 6.** Candidate Sets of Features Considered for Inclusion in the TBI

**eAppendix 11.** Results From 1000 Split-Sample-Simulation Cross-validation

**eTable 7.** Results From 1000 Split-Sample Simulation Cross-validation

**eFigure 4.** Subgroup-Specific OR Distributions Obtained From a 1000 Split-Sample Simulation Cross-validation

**eTable 8.** Value Results From the 1000 Split-Sample Simulation Cross-validation

**eAppendix 12.** Results From Leave-One-RCT-Out Cross-validation

**eTable 9.** Results From a Leave-One-RCT-Out Cross-validation

**eTable 10.** Value Results From a Leave-One-RCT-Out Cross-validation

**eAppendix 13.** Results From Leave-One-Enrollment-Quarter-Out Cross-validation

**eTable 11.** Results From a Leave-One-Enrollment-Quarter-Out Cross-validation

**eTable 12.** Results From a Leave-One-Enrollment-Quarter-Out Cross-validation, Before Averaging Across the 4 Enrollment Quarters (Q2-Q5) for the Basic and Expanded TBIs

**eTable 13.** Value Results From a Leave-One-Enrollment-Quarter-Out Cross-validation

**eAppendix 14.** Comparisons With a Naive Treatment Decision Approach

**eFigure 5.** Distributions of Subgroup-Specific ORs Obtained From 1000 Randomly Split Testing Sets for the Ordinal Outcome WHO Score at Day 14

**eFigure 6.** Distributions of Subgroup-Specific ORs Obtained From 1000 Randomly Split Testing Sets for the Ordinal Outcome WHO Score at Day 28

**eFigure 7.** Distributions of Subgroup-Specific ORs Obtained From 1000 Randomly Split Testing Sets for Binary Outcome of WHO Score of at Least 7 at Day 14

**eFigure 8.** Distributions of Subgroup-Specific ORs Obtained From 1000 Randomly Split Testing Sets for Binary Outcome of WHO Score of at Least 7 at Day 28

**eFigure 9.** Distributions of Subgroup-Specific ORs Obtained From 1000 Randomly Split Testing Sets for Death at Day 14

**eFigure 10.** Distributions of Subgroup-Specific ORs Obtained From 1000 Randomly Split Testing Sets for Death at Day 28

**eAppendix 15.** Specification of the Basic and Expanded TBIs

**eTable 14.** Coefficients of the Linear Combination for Baseline Patient Characteristics in the Basic and Expanded TBIs

**eAppendix 16.** Cross-Stratification Table Comparing the Basic and Expanded TBIs

**eTable 15.** Benefit Stratification Table

**eAppendix 17.** Proportional Odds Assumption Test

**eTable 16.** Estimates of Coefficients Associated With HTE

**eTable 17.** Brant Test Results

**eAppendix 18.** Three Levels of Benefit for the Basic and Expanded TBIs

**eFigure 11.** Basic TBI Model-Based Cumulative Odds Ratio for the Ordinal WHO Score at Day 14, as a Function of the Basic TBI

**eFigure 12.** Expanded TBI Model-Based Cumulative Odds Ratio for the Ordinal WHO Score at Day 14, as a Function of the Expanded TBI

**eTable 18.** Odds Ratios Associated with All 6 Outcomes, Under 3 Levels of Categorization From the Basic and Expanded TBIs

**eTable 19.** Results Regarding the Expected Incidence (Value) of the 4 Clinically Undesirable Binary Outcomes When CCP is Administered to the 3 Patient Subgroups Identified by the Basic and Expanded TBIs

**eAppendix 19.** Baseline Patient Characteristics by Benefit Levels

**eTable 20.** Baseline Characteristics of COMPILE Patients in the 3 Benefit-Level Groups Defined From the Expanded TBI

**eFigure 13.** Proportions of the 3 Benefit-Level Groups Identified From the Expanded TBI, by Quarter

**eTable 21.** Baseline Patient Characteristics for the Subgroup of Patients Who Would and Would Not Be Advised to Receive CCP, Determined by the Expanded TBI

**eAppendix 20.** CCP Efficacy in the 3 Benefit Groups With Respect to All 6 Outcomes, Determined by the Expanded TBI

© 2022 Park H et al. *JAMA Network Open.*

**eTable 22.** Posterior Probability Distributions of the ORs of CCP vs Control for All Outcomes, by Benefit Group Determined From the Expanded TBI

**eFigure 14.** Forest Plots of Median ORs and 95% Credible Intervals From the Posterior Probability Distributions for the ORs by Benefit Groups Determined From the Expanded TBI for All 6 Outcomes

**eAppendix 21.** Differential Treatment Effect by Benefit Levels, in Comparison With Differential Treatment Effect by Single Patient Characteristics

**eFigure 15.** Odds Ratios and 95% Credible Intervals for Subgroup-Specific Odds for Individual Baseline Covariates and 3 Benefit Groups for WHO Score on Day 14

**eFigure 16.** Odds Ratios and 95% Credible Intervals for Subgroup-Specific Odds for Individual Baseline Covariates and 3 Benefit Groups for WHO Score 7 to 10 on Day 14

**eFigure 17.** Odds Ratios and 95% Credible Intervals for Subgroup-Specific Odds for Individual Baseline Covariates and 3 Benefit Groups for WHO Score on Day 28

**eFigure 18.** Odds Ratios and 95% Credible Intervals for Subgroup-Specific Odds for Individual Baseline Covariates and 3 Benefit Groups for WHO Score 7 to 10 on Day 28

**eFigure 19.** Odds Ratios and 95% Credible Intervals for Subgroup-Specific Odds for Individual Baseline Covariates and 3 Benefit Groups for Mortality on Day 14

**eFigure 20.** Odds Ratios and 95% Credible Intervals for Subgroup-Specific Odds for Individual Baseline Covariates and 3 Benefit Groups for Mortality on Day 28

**eAppendix 22.** CCP Benefit as a Function of the Basic TBI

**eFigure 21.** CCP Benefit as a Function of the Basic TBI

**eAppendix 23.** Baseline Patient Characteristics by Benefit Levels Determined From the Basic TBI

**eTable 23.** Baseline Characteristics of COMPILE Patients in the 3 Benefit-Level Groups Defined by the Basic TBI

**eFigure 22.** Proportions of the 3 Benefit Groups (B1, B2 and B3) Identified From the Basic TBI, by Quarter

**eTable 24.** Baseline Patient Characteristics for the Subgroup of Patients Who Would and Would Not Be Advised to Receive CCP, Determined by the Basic TBI

**eAppendix 24.** CCP Efficacy in the 3 Benefit Groups With Respect to All 6 Outcomes, Determined by the Basic TBI

**eTable 25.** Posterior Probability Distributions of the Odds Ratios of CCP vs Control for All Outcomes, by Benefit Group Determined From the Basic TBI

**eFigure 23.** Forest Plots of Median Odds Ratios and 95% Credible Intervals From the Posterior Probability Distributions for the ORs by Benefit Groups Determined From the Basic TBI for All 6 Outcomes

**eAppendix 25.** Kaplan-Meier Plots for Time to Death and Time to Hospital Discharge, With Log-Rank Test for Time to Death and Gray Competing Risk Test for Time to Hospital Discharge, Basic TBI

**eFigure 24.** Comparison Between CCP and Control Patients for Time to Death and Time to Hospital Discharge by Benefit Level, Determined by the Basic TBI

**eAppendix 26.** Validation on Expanded Access Program (EAP) Data

**eTable 26.** Baseline Characteristics for Participants Treated Under the EAP With Available Data for All Specified Parameters

**eTable 27.** Summary of Balance for Matched Data for EAP

**eTable 28.** Summary of Balance for Matched Data for EAP, High Titer Only

**eTable 29.** Participants in the Matched Data, by Benefit Levels Defined by the Expanded and the Basic TBIs and Depending on Whether All EAP Patients or Only Those Treated With High-Titer Plasma Were Used For Validation

© 2022 Park H et al. *JAMA Network Open.*

**eTable 30.** CCP Benefit Odds Ratios Within Different Benefit Levels Defined by the Basic and the Expanded TBIs and Depending on Whether Subgrouping Was Restricted to Patients with High-Titer CCP

**eAppendix 27.** Validation on an Emergency Use Authorization (EUA) Study

**eTable 31.** Baseline Characteristics of CCP Recipients at Montefiore Medical Center, Bronx, NY

**eTable 32.** Summary of Balance for Matched Data for the EUA

**eTable 33.** Patients in Different Benefit Levels defined by the Basic TBI and the Expanded TBI for the EUA

**eTable 34.** Subgroup-Specific CCP Benefit Odds Ratios for Benefit Levels Defined by the Basic TBI and the Expanded TBI for the EUA

**eAppendix 28.** Validation on External RCT 1

**eTable 35.** Baseline Patient Characteristics and Clinical Information of the First RCT

**eTable 36.** Major Pharmacotherapy as the Standard of Care

**eTable 37.** Patients in Different Benefit Levels Based on the Basic TBI in the First RCT

**eTable 38.** Subgroup-Specific CCP Benefit Odds Ratios With Respect to 30-Day Mortality, by Benefit Level Defined by the Basic TBI, in the First RCT

**eFigure 25.** CCP vs Control as a Function of the Basic TBI (Which Does Not Require Blood Type Information), With the 3 Benefit Groups Overlaid, Evaluated on the First External RCT

**eAppendix 29.** Validation on External RCT 2

**eTable 39.** Baseline Patient Characteristics for the Second RCT

**eTable 40.** Patients in Different Benefit Levels Based on the Expanded and Basic TBIs for the Second RCT

**eTable 41.** CCP Benefit Odds Ratios for Ordinal Outcomes at Days 14 and 30 for the Benefit Groups Defined by the Basic and Expanded TBIs, Evaluated on the Second External RCT

**eFigure 26.** CCP Benefit Odds Ratios for WHO 6-Point Scale Ordinal Outcomes at Days 14 and 30, as Functions of the Basic and Expanded TBIs, Evaluated on the Second External RCT

**eTable 42.** CCP Benefit Odds Ratios for the Binary Outcome of Ventilation or Worse at Days 14 and 30 for Benefit Groups Defined by the Basic and Expanded TBIs, Evaluated on the Second External RCT

**eFigure 27.** CCP Benefit Odds Ratios for Binary Outcome of Ventilation or Worse at Days 14 and 30 for Benefit Groups Defined by the Basic and Expanded TBIs, Evaluated on the Second External RCT

**eAppendix 30.** More Results From the Expanded TBI

**eFigure 28.** Probability of the 11-Point WHO Ordinal Scales Categorized into 4 Ordinal Categories, as a Function of the Expanded TBI

**eAppendix 31.** Response Surface and Scatterplots Associated With the Expanded TBI Model

**eFigure 29.** Fitted POM

**eFigure 30.** Fitted POM Displayed on the Probability Scale

**eFigure 31.** Scatterplot of the Risk Index and the TBI in the Fitted POM

**eFigure 32.** Scatterplot of the Risk Index and the TBI-by-Treatment Interaction Term in the Fitted POM

**eFigure 33.** Empirical Cumulative Distribution Functions for the Basic and Expanded TBIs With the Associated Benefit Levels Overlaid

This supplemental material has been provided by the authors to give readers additional information about their work.

© 2022 Park H et al. *JAMA Network Open.*

## eAppendix 1. TBI Models and Abbreviations

The development of the treatment benefit index uses the regression framework of single index models with multiple links (SIMML)[1,2] and is based on an extension for the case of ordinal categorical outcomes[3]. It uses proportional odds models (POM) with cumulative probabilities and can capture nonlinear associations between a combination of pretreatment covariates ("single-index") and an ordinal treatment outcome from two or more alternative therapies (here CCP and control). The combination of covariates is referred to, in the statistical literature, as a "single-index" and in the context of precision medicine it is a linear combination of patient pretreatment characteristics, which is termed the treatment benefit index (TBI) in the manuscript.

For the development of TBI, the log-odds of the ordinal outcome $WHO \geq y$, for $y = 1,2,\ldots,10$, are modeled through a POM:

$$logit\big(P(Y \geq y)\big) \;=\; \tau_y + \mu(\boldsymbol{Z}) + \; g(\boldsymbol{\beta}^T\boldsymbol{X}, A), \qquad y = 1,2,\ldots,10, \qquad\qquad (S1.1)$$

where the variable $A$ is the treatment indicator taking a value in either 1 (CCP) or 0 (control), and the linear combination of pre-treatment covariates $\boldsymbol{X}$ (i.e., $\boldsymbol{\beta}^T\boldsymbol{X}$) is referred to as a "single-index" or a CCP-TBI, or just TBI. In (S1.1), $\tau_y$ are cut-point parameters associated with the ordered response categories $y = 1,2,\ldots,10$, satisfying the property $\tau_1 > \tau_2 > \cdots > \tau_{10}$, and $\boldsymbol{Z}$ also represents pre-treatment covariates, but it can be different from $\boldsymbol{X}$.

In POM (S1.1), the heterogeneous CCP effect is modeled by the component $g(\boldsymbol{\beta}^T\boldsymbol{X}, A)$, which is the crucial part determining the TBI. On the other hand, the term $\mu(\boldsymbol{Z})$ in (S1.1), that models the covariates' main effects, have no impact on the differential effect of the CCP treatment and are used to factor out variation common in the outcomes under both treatments (here CCP and control) to improve the performance of the TBI.

For model identifiability, in (S1.1), we impose an identifiability constraint $E[g(\boldsymbol{\beta}^T\boldsymbol{X}, A)\,|\boldsymbol{X}] = \; 0$ on $g$. This makes the component $g(\boldsymbol{\beta}^T\boldsymbol{X}, A)$ orthogonal to $\mu(\boldsymbol{Z})$ in equation (S1.1) (see [2,3] for details of the identifiability condition). Then the covariates' main effect $\mu(\boldsymbol{Z})$ in (S1.1) can be estimated separately based on the model

$$logit\big(P(Y \geq y)\big) \;=\; \tilde{\tau}_y + \mu(\boldsymbol{Z}), \qquad y = 1,2,\ldots,10, \qquad\qquad (S1.2)$$

where $\tilde{\tau}_y$ are unknown cut-point parameters associated with the ordered response categories $y = 1,2,\ldots,10$ (with the property $\tilde{\tau}_1 > \tilde{\tau}_2 > \cdots > \tilde{\tau}_{10}$) and is augmented in the estimation of model (S1.1) (see [2,4] for the efficiency augmentation). For model parsimony, we restricted the functional form of $\mu(\boldsymbol{Z})$ to be linear with respect to the covariates $\boldsymbol{Z}$ (including transformation and interactions of patient characteristics).

Akaike Information Criteria (AIC) was used for selecting the covariates $\boldsymbol{Z}$ in the main effect. The interaction term $g(\boldsymbol{\beta}^T\boldsymbol{X}, A)$ in the TBI model (S1.1) has two elements that are estimated simultaneously: a) the coefficient of linear combination, $\boldsymbol{\beta}$, that forms the TBI (i.e., $\boldsymbol{\beta}^T\boldsymbol{X}$); and b) the link function, $g(.,.)$, relating the TBI with the treatment outcomes. Regarding b), we use a penalized spline representation for $g(.,.)$, allowing for the relationship to be nonlinear if that provided a better fit to the data.

---

[1] Park H, Petkova E, Tarpey T, Ogden RT. A single-index model with multiple-links. *J Stat Plan Inference.* 2020;205:115-128.

[2] Park H, Petkova E, Tarpey T, Ogden RT. A constrained single-index regression for estimating interactions between a treatment and covariates. *Biometrics.* 2020.

[3] Park H, Petkova E, Tarpey T, Ogden RT. A single-index model with a surface-link for optimizing individualized dose rules. *Journal of Computational and Graphical Statistics.* 2021:1-30.

[4] Tian L, Alizadeh A, Gentles A, Tibshirani R. A simple method for estimating interactions between a treatment and a large number of covariates. *Journal of the American Statistical Association*. 2014;109:508, 1517-1532.

© 2022 Park H et al. *JAMA Network Open.*

The covariates $\boldsymbol{X}$ in the TBI are selected based on cross-validation (CV) (with respect to a criterion specified in Section eAppendix 2) and the smoothing parameter associated with the function $g(.,.)$ are selected based on restricted maximum likelihood estimation (REML) to avoid overfitting. Given the augmentation term $\mu(\boldsymbol{Z})$ and a fixed set of covariates $\boldsymbol{X}$ in the TBI, model (S1.1) is optimized with respect to the model likelihood (over the components $g, \boldsymbol{\beta}$ and $\tau_j$), subject to the constraint $E[g(\boldsymbol{\beta}^T\boldsymbol{X}, A) \,|\boldsymbol{X}] = 0$ on $g$. The procedure iterates between the optimization of $(g, \tau_j)$ and that of $\boldsymbol{\beta}$, until convergence of the model components (see[5] for details of the estimation procedure). Upon convergence, we rescale $g$ and $\boldsymbol{\beta}$ in such a way that the observed range of the TBI, $\boldsymbol{\beta}^T\boldsymbol{X}$, in model (S1.1) is restricted to the interval $[0, 1]$, to facilitate interpretability of the TBI.

Under the cumulative logit POM (S1.1), the cumulative odds ratio function, denoted as $f(.)$, evaluating the efficacy of CCP ($A = 1$) vs. control ($A = 0$) over the TBI $\boldsymbol{\beta}^T\boldsymbol{X}$, is

$$f(\boldsymbol{\beta}^T\boldsymbol{X}) = \exp\{g(\boldsymbol{\beta}^T\boldsymbol{X}, A = 1) - g(\boldsymbol{\beta}^T\boldsymbol{X}, A = 0)\}. \qquad (S1.3)$$

Specifically, a cumulative odds ratio (OR) less than 1, i.e., $f(\boldsymbol{\beta}^T\boldsymbol{X}) < 1$, indicates that a patient with a particular TBI value $\boldsymbol{\beta}^T\boldsymbol{X}$ is expected to benefit from CCP, whereas the cumulative OR greater than or equal to 1, i.e., $f(\boldsymbol{\beta}^T\boldsymbol{X}) \geq 1$, indicates that such a patient is not expected to benefit from CCP.

In general, if the function $f(\cdot)$ is not monotone with respect to $\boldsymbol{\beta}^T\boldsymbol{X}$, then the TBI can be more generally defined as: $U = g(\boldsymbol{\beta}^T\boldsymbol{X}, A = 0)$. Due to the constraint $E[g(\boldsymbol{\beta}^T\boldsymbol{X}, A) \,|\boldsymbol{X}] = 0$ that we imposed on the data-driven function $g$ of the model (1), we have $g(\boldsymbol{\beta}^T\boldsymbol{X}, A = 1)P(A = 1) + g(\boldsymbol{\beta}^T\boldsymbol{X}, A = 0)P(A = 0) = 0$, implying $g(\boldsymbol{\beta}^T\boldsymbol{X}, A = 1) = -U\frac{P(A=0)}{P(A=1)}$, where $P(A = 0)$ and $P(A = 1)$ are known randomization probabilities in the context of RCTs. Thus, the function $f(\cdot)$ in (S1.3) can be reparametrized to: $f(U) = \exp\{-U(\frac{P(A=0)}{P(A=1)} + 1)\}$, which is a monotone function of the TBI $U$, based on which a continuous gradation of the expected benefit from CCP can be provided. However, in this TBI analysis, since the data-driven function $f(\cdot)$ was already monotone with respect to $\boldsymbol{\beta}^T\boldsymbol{X}$, we simply employed $\boldsymbol{\beta}^T\boldsymbol{X}$ as the CCP-TBI.

[5] Park H, Petkova E, Tarpey T, Ogden RT. A single-index model with a surface-link for optimizing individualized dose rules. *Journal of Computational and Graphical Statistics.* 2021:1-30

© 2022 Park H et al. *JAMA Network Open.*

## Abbreviations

| | |
|---|---|
| AIC | Akaike information criteria |
| CCP | COVID-19 convalescent plasma |
| COMPILE | Continuous monitoring of pooled international trials of convalescent plasma for COVID-19 hospitalized patients |
| CV | Cross-validation |
| HTE | Heterogeneous treatment effect |
| IPD | Individual patient data |
| OR | Odds ratio |
| PATH | Predictive approaches to treatment effect heterogeneity |
| POM | Proportional odds model |
| RCT | Randomized clinical trial |
| SAP | Statistical analysis plan |
| SOC | Standard of care |
| TBI | Treatment benefit index |
| TDR | Treatment decision rule |
| WHO | World Health Organization |

© 2022 Park H et al. *JAMA Network Open.*

## eAppendix 2. Workflow and Internal Cross-validation

The patient pretreatment characteristics $\boldsymbol{X}$ that enter into the TBI $\boldsymbol{\beta}^T\boldsymbol{X}$ in model (S1.1 in eAppendix 1) are selected based on internal cross-validation (CV) for the following criteria.

1. The ratio of the CCP efficacy ORs between the group predicted to benefit (B) and the group predicted to not benefit (NB). By using the efficacy criterion of the cumulative OR=1 for the model-based cumulative OR (S1.3), the patient population can be stratified into the subpopulation B and the subpopulation NB. To quantify the differential CCP benefit between B and NB, we calculate the OR for the CCP efficacy for each subgroup (B and NB), denoted as OR(B) and OR(NB), respectively, and then compute the ratio of these two ORs, i.e., OR(B)/OR(NB). Specifically, a smaller ratio that satisfies OR(B)/OR(NB) < 1 indicates good TBI performance, in terms of separating B and NB, with respect to differential CCP efficacy. We use the ordinal WHO scale at day 14 (which was the co-primary outcome of the study) to train the TBI model, which is then evaluated on all outcomes on a validation set: the ordinal WHO scale, the indicator of WHO ≥ 7, and the indicator of WHO=10 (at day 14 and at day 28).

2. The "value"[6] of treatment classification rules derived from the respective TBIs. Specifically, for any given treatment classification rule, we compute the expected proportions (i.e., the incidences— the so-called "values") associated with the clinically undesirable binary outcomes— the indicator of WHO ≥ 7 and the indicator of WHO=10 at day 14 and at day 28— when the treatment is assigned according to the treatment classification rule. Smaller expected values indicate better treatment classification rules and, correspondingly, better TBIs. These expected values were not computed for ordinal WHO scores, because means (or medians) are not appropriate summaries for ordinal data.

A set of different TBI models (with different variables $\boldsymbol{X}$ entering into the corresponding TBIs) with comparable AIC values were first identified as a set of "candidate" TBI models (see eTable 5). Then further extensive CV was conducted to select the final model from the set of these candidates. The model development workflow through internal CVs focusing on the selection of the variables $\boldsymbol{X}$ is presented on eFigure 1. The respective CV procedures are described in eAppendix 3-5.

[6] Qian M, Murphy SA. Performance guarantees for individualized treatment rules. *Annals of Statistics.* 2011;39(2)

© 2022 Park H et al. *JAMA Network Open.*

## eFigure 1. Workflow Diagram for Developing the TBI

The focus was on identifying the robust features to optimize the TBI performance for treatment classification. The model performance was assessed with CV, evaluated with respect to the outcome of: (1) ordinal WHO 11-point scale at days 14 and 28; (2) binary indicator of WHO ≥ 7 at days 14 and 28; (3) binary indicator of WHO=10 (mortality) at days 14 and 28.

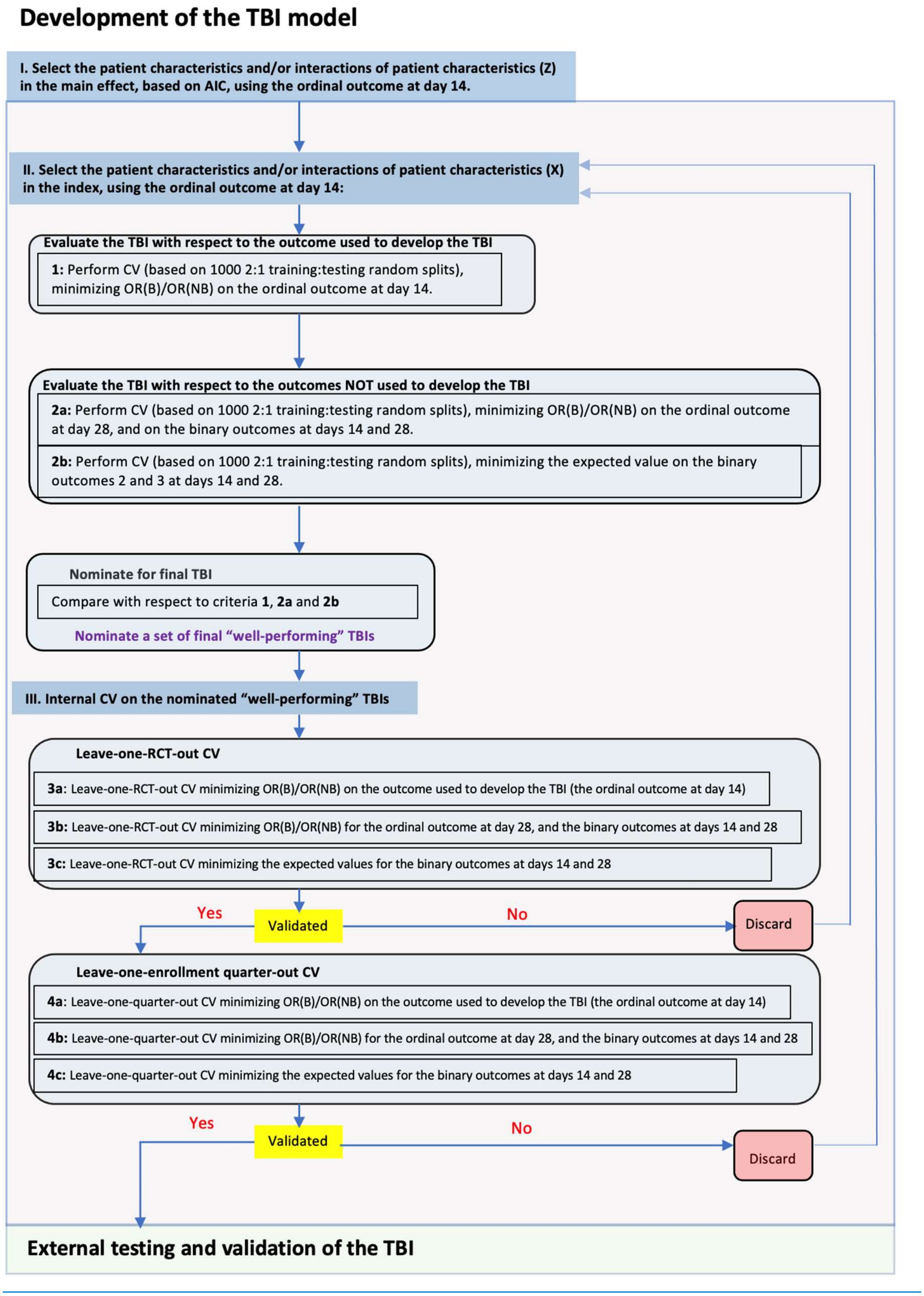


© 2022 Park H et al. *JAMA Network Open.*

## eAppendix 3. Split-Sample-Simulation Cross-validation, 1000 replications

The candidate models were compared with respect to how well they performed for separating the benefit groups, via an extensive CV. Specifically, we perform a split sample simulation, repeating 1000 times, to compare treatment classifiers derived from TBIs. Each time, we randomly partition the data, derivation of classifiers in one part (2/3$^{rds}$) of the data, with subsequent test of the classifier in the remaining part (1/3$^{rd}$) of the data. We first record each classifier's subgroup specific ORs and the ratio, OR(B)/OR(NB), computed in the validation sample. The median of these measures obtained from the 1000 replications was used to compare different TBI models. The TBI models were ranked on the basis of their CV performance, with respect to the outcome used to train the TBI – day 14 ordinal WHO score. This corresponds to Step 1 of part II from the workflow diagram in eFigure 1. This step resulted in sets of TBIs performing well and those with clearly poorer performance. All "well-performing" TBIs were then tested with respect to the remaining five outcomes: the ordinal WHO score at day 28, binary indicator of WHO ≥ 7 at days 14 and 28, and the binary indicator WHO=10 at days 14 and 28. This corresponds to Step 2a of part II of the workflow diagram in eFigure 1. In addition, we compared the "values" of the treatment classifiers, evaluated with respect to the binary outcomes (the indicators of WHO ≥ 7 and WHO=10, at days 14 and 28), where smaller "values" (i.e., the proportions of patients with the bad events) indicate a better performance. This internal CV with respect to the "values" of the treatment classifiers corresponds to Step 2b of part II of the workflow diagram in eFigure 1. Based on those analyses, a small final set of best TBIs was nominated.

© 2022 Park H et al. *JAMA Network Open.*

## eAppendix 4. Leave-One-RCT-Out Cross-validation

As another internal validation criterion for developing the TBIs, a leave-one-RCT-out CV was used as a proxy for an external validation for selecting the patient covariates used in the models. There were 4 large RCTs (RCT IDs: AA, CC, EE, KK) participating in COMPILE and the remaining 4 trials (RCT IDs: BB, DD, GG, RR) were pooled to form a larger combined RCT to be used in the leave-one-RCT-out CV, see eTable 1. We hold out each RCT (among AA, CC, EE, KK and "combined") and train the TBI models on the remaining 4 RCTs. Using the trained classifiers, patients in the held-out RCT were then classified into B and NB, respectively, and the corresponding CCP efficacy ORs, denoted as OR(B) and OR(NB) respectively, and their ratio, OR(B)/OR(NB), were computed with respect to each of the six outcomes. By repeating this step for all RCTs, the leave-one-RCT-out ORs were averaged across all five once-held-out RCTs (AA, CC, EE, KK and "combined"). A similar procedure was also performed to compare classifiers with respect to the "values". The TBI models with the smallest averaged ratio OR(B)/OR(NB) for all or most of the six outcomes and the smallest expected values for all or most of the four binary outcomes were elicited. This corresponds to Steps 3a, 3b and 3c of part III from the workflow diagram in eFigure 1.

© 2022 Park H et al. *JAMA Network Open.*

## eTable 1. RCTs Participating in COMPILE

| RCT ID | Control condition | Units of CCP | Control n | CCP n |
|---|---|---|---|---|
| AA (n=941) | Saline (n=941) | 1 | 473 | 468 |
| DD (n=34) | Non-convalescent plasma (n=34) | 1 | 18 | 16 |
| BB (n=80) | Standard of care (n=1394) | 2 | 39 | 41 |
| CC (n=350) | | 1 | 171 | 179 |
| EE (n=477) | | 4 | 163 | 314 |
| GG (n=34) | | 1 | 15 | 19 |
| KK (n=381) | | 2 | 224 | 157 |
| RR (n=72) | | 1 | 35 | 37 |
| Total | n=2369 | | 1138 | 1231 |

## eAppendix 5. Leave-One-Enrollment-Quarter-Out Cross-validation

Another CV used in developing of the TBIs was based on time of the patients' enrollment in the study. The main analysis of the COMPILE study identified differences in the efficacy of CCP during the 4 quarters from April 2020 to March 2021 when patients were enrolled in the RCTs. While the effects of quarter of enrollment on the outcomes in the control group were expected to change over time due to the changing standards for usual care for COVID-19 patients, we also observed some differences in the relative efficacy of CCP (compared to control) during different quarters with respect to the six outcomes of interest, see eFigure 2 below. One possible explanation for this shift in the efficacy is that the distributions of patient characteristics differed across the quarters. An alternative explanation could be that the efficacy of CCP (vs. control) was not the same in the different quarters even for patients with the same baseline profiles. To investigate this question, similar to the leave-one-RCT-out approach, we performed leave-one-enrollment-quarter-out CV. This step corresponds to 4a, 4b and 4c of part III from the workflow diagram in eFigure 1.

© 2022 Park H et al. *JAMA Network Open.*

## eFigure 2. Median ORs and 95% Credible Intervals From the Posterior Probability Distributions for the ORs, by Patient Enrollment Quarters

The posterior distributions are estimated from the Bayesian cumulative proportional odds models for the ordinal WHO scores and logistic regression, adjusted for age, sex, baseline WHO scores, duration of symptoms prior to treatment, diabetes, cardiovascular disease, pulmonary disease and quarter of enrollment.

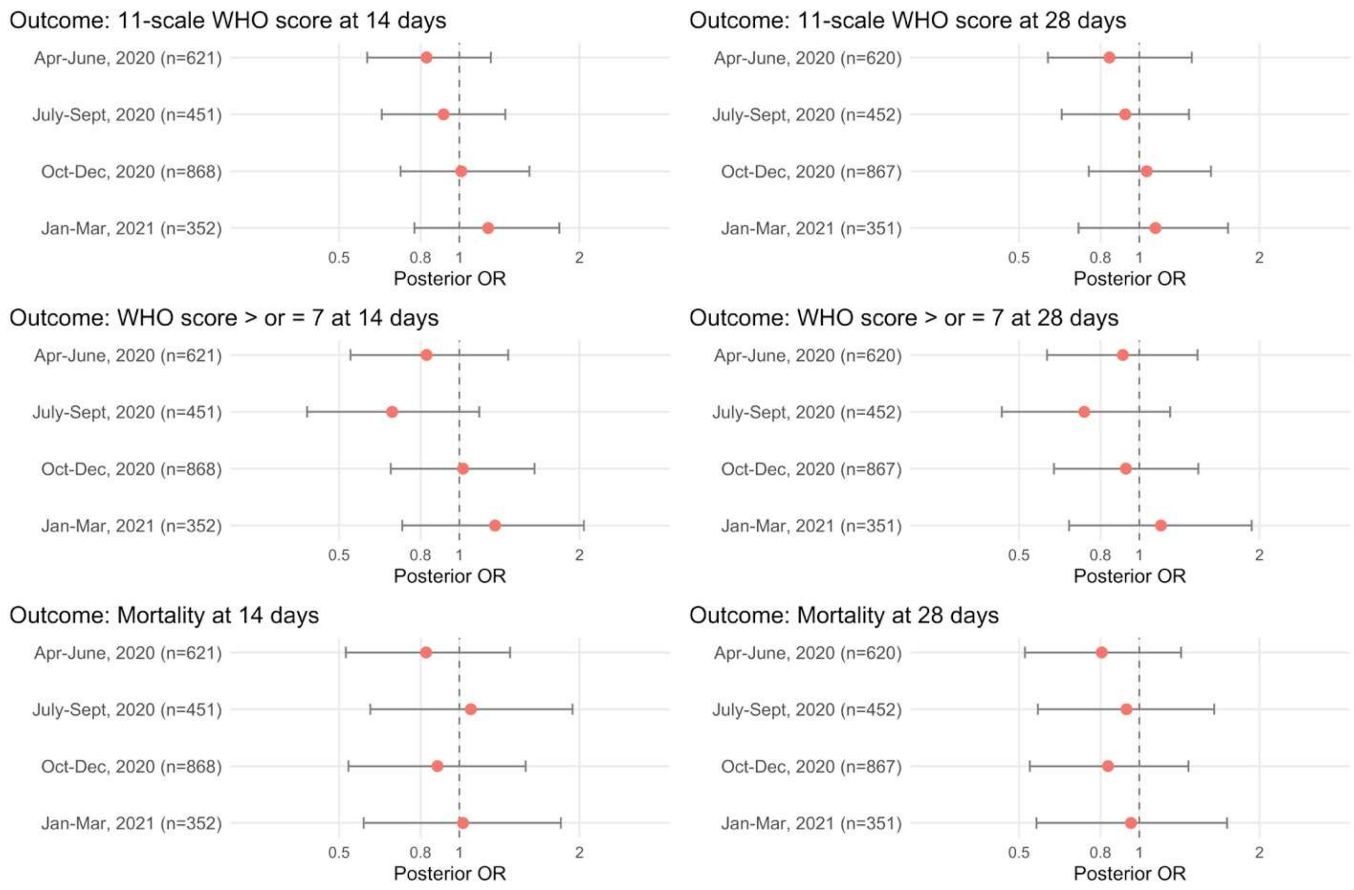


© 2022 Park H et al. *JAMA Network Open.*

## eAppendix 6. Determination of 3 Levels of Benefit

For clinical practice it might be convenient to create categories rather than use the TBI as a continuous measure. The expected benefit from CCP treatment might be usefully described as having three levels: (B1) large benefit, (B2) modest benefit, and (B3) potential harm expected. To determine the cut-off points for creating three levels of benefit, we employed a likelihood criterion as follows. For a given pair of cut-off points (a, b), we define from the TBI a grouping variable with 3 levels: TBI $< a$; $a \leq$ TBI $< b$; and TBI $\geq b$, that is, a 3-levels categorical TBI. Then, we use POM to regress the ordinal WHO scale at day 14 (i.e., the outcome used in the development of the TBI) on the 3-levels categorized TBI, the treatment indicator and the interaction between the two (as in the estimation of the POMs displayed in Figure 1 of the main manuscript, but we used the 3-level categorical TBI), and assess the model likelihood. This POM with a categorized TBI has a constant effect constraint of TBI within each of the three intervals defined by (a, b), making it effective in determining groups when there are points of inflection in the OR (as in Figure 1 of the main manuscript) with respect to TBI. A grid of pairs of cut-off points is considered and the pair of cut-off points that results in the highest likelihood is selected to define three levels of expected benefit from treatment with CCP.

© 2022 Park H et al. *JAMA Network Open.*

## eAppendix 7. Application of Other Methods for Treatment Classification Rules

An advantage of the single-index POM (S1.1 in eAppendix 1) approach used here is that, in addition to a binary treatment classifier (that identifies a subpopulation expected to benefit from CCP vs. not benefit from CCP), the index provides a continuous, easy-to-compute gradation of the benefit. Most other precision medicine methodologies in the statistical literature under the rubric of treatment classification rules do not provide such gradient of benefit. Still, we compared the results from several popular machine learning methods to our selected TBI index, in terms of OR(B), OR(NB) and the ratio of ORs OR(B)/OR(NB), as well as in terms of the "values."

The following alternative methods were used: Q-learning[7] trained with (1) the original outcome and (2) the residuals as outcome; Outcome Weighted Learning (3 and 4) trained with the original ordinal WHO score at day 14 as outcome (OWL[8]) and with Residuals as weights (RWL[9]) both with linear kernel and radial basis function (RBF) kernel; (5-12) Robust Index of Confidence Weighted Learning (RICWL) trained with the original outcome and with residuals, with both linear and RBF kernels, each with both cosine-based and fraction-based similarity measures[10]; and (13 and 14) boosting[11]. Our TBI index performed superiorly – the results are given in eTable 2 and eTable 3 for these alternative methods considered; the TBI results are given in eTable 7 and eTable 8.

---

[7] Qian M, Murphy SA. Performance guarantees for individualized treatment rules. *Annals of Statistics.* 2011;39(2).
[8] Zhao Y, Zeng D, Rush AJ, Kosorok MR. Estimating individualized treatment rules using outcome weighted learning. *Journal of the American Statistical Association.* 2012;107(499):1106-1118.
[9] Zhou X, Mayer-Hamblett N, Khan U, Kosorok M. Residual weighted learning for estimating individualized treatment rules. *Journal of the American Statistical Association*, 2017:112(517); 169–187
[10] Zhang J, Troxel AB, Petkova E. Robust index of confidence weighted learning for optimal individualized treatment rule estimation. *Stat*. 2021;10:e374
[11] Zhang P, Ma J, Chen X, Shentu Y. A nonparametric method for value function guided subgroup identification via gradient tree boosting for censored survival data. *Statistics in Medicine*. 2020;39(28): 4133-4146

© 2022 Park H et al. *JAMA Network Open.*

## eTable 2. Results in Terms of Odds Ratios From Cross-validation Based on 400 Splits Into Training Data Set and Validation Data Set

The treatment classifiers are developed using these alternative methods. Since these approaches do not currently accommodate ordinal outcomes, these methods are trained based either on the ordinal WHO scale at day 14 treated as a continuous outcome or on the indicator of WHO ≥ 7 at day 14 treated as a binary outcome. Based on a treatment classification rule developed on the training set, the validation set is stratified into a group that is expected to benefit (B) from CCP and a group that is not expected to benefit (NB) from CCP. For the ordinal WHO and binary WHO ≥ 7 (at days 14 and 28) outcomes, the ORs comparing CCP vs. control are evaluated within the subgroup B and the subgroup NB of the validation set, and their ratios OR(B)/OR(NB) are computed. The table entries show the median of ORs (in the columns OR(B) or OR(NB)) and of the ratios of these ORs (in the columns B/NB) (a smaller ratio indicates a better performance) across the 400 splits.

| Method | Outcomes | | | | | | Outcomes | | | | | |
|---|---|---|---|---|---|---|---|---|---|---|---|---|
| | Day 14 WHO ordinal | | | Day 28 WHO ordinal | | | Day 14 WHO ≥ 7 | | | Day 28 WHO ≥ 7 | | |
| | OR(B) | OR(NB) | B/NB | OR(B) | OR(NB) | B/NB | OR(B) | OR(NB) | B/NB | OR(B) | OR(NB) | B/NB |
| Q-Learning, with original outcomes | 0.867 | 0.967 | 0.882 | 0.870 | 1.031 | 0.833 | 0.733 | 1.071 | 0.664 | 0.723 | 0.999 | 0.702 |
| Q-Learning, using residuals | 0.868 | 0.961 | 0.904 | 0.750 | 1.073 | 0.679 | 0.881 | 1.018 | 0.853 | 0.748 | 1.000 | 0.741 |
| OWL- Linear kernel | 0.882 | 0.919 | 0.942 | 0.762 | 1.447 | 0.527 | 0.902 | 0.949 | 0.934 | 0.764 | 1.148 | 0.646 |
| RWL- Linear kernel | 0.881 | 0.908 | 1.026 | 0.825 | 0.976 | 0.756 | 0.889 | 0.924 | 0.948 | 0.802 | 0.874 | 0.859 |
| RICWL-Fraction, with original outcomes, RBF kernel | 0.891 | 0.863 | 0.993 | 0.812 | 0.892 | 0.879 | 0.916 | 0.921 | 0.963 | 0.794 | 0.866 | 0.884 |
| RICWL-Fraction, using residuals, RBF kernel | 0.902 | 0.889 | 1.009 | 0.820 | 0.978 | 0.828 | 0.926 | 0.939 | 0.981 | 0.805 | 0.900 | 0.857 |
| RICWL-Fraction, with original outcomes, linear kernel | 0.891 | 0.873 | 0.998 | 0.793 | 0.897 | 0.862 | 0.905 | 0.908 | 0.979 | 0.764 | 0.864 | 0.876 |
| RICWL-Fraction, using residuals, linear kernel | 0.895 | 0.872 | 1.013 | 0.778 | 0.996 | 0.735 | 0.909 | 0.914 | 0.965 | 0.762 | 0.909 | 0.816 |
| RICWL-Cosine, with original outcomes, RBF kernel | 0.885 | 0.803 | 1.078 | 0.847 | 0.812 | 1.050 | 0.919 | 0.886 | 0.988 | 0.816 | 0.769 | 1.043 |
| RICWL- Cosine, using residuals, RBF kernel | 0.872 | 0.868 | 0.990 | 0.795 | 0.943 | 0.816 | 0.901 | 0.919 | 0.948 | 0.775 | 0.871 | 0.869 |
| RICWL- Cosine, with original outcomes, linear kernel | 0.909 | 0.761 | 1.179 | 0.840 | 0.749 | 1.059 | 0.930 | 0.782 | 1.209 | 0.817 | 0.715 | 1.167 |
| RICWL- Cosine, using residuals, linear kernel | 0.867 | 0.831 | 1.028 | 0.763 | 0.963 | 0.732 | 0.895 | 0.861 | 1.045 | 0.765 | 0.842 | 0.854 |
| Boosting, using continuous outcomes | 0.895 | 0.895 | 0.958 | 0.824 | 0.902 | 0.863 | 0.916 | 0.907 | 0.974 | 0.812 | 0.848 | 0.899 |
| Boosting, using binary outcomes | 0.881 | 0.931 | 0.902 | 0.778 | 1.065 | 0.671 | 0.901 | 0.949 | 0.945 | 0.775 | 0.988 | 0.709 |

For these alternative methods, we also evaluate the “value” of the associated treatment decision rules, with respect to the four binary outcome measures (i.e., the indicator of WHO ≥ 7 and the indicator of death, WHO=10, at days 14 and 28). These values are compared with those of two “treatment policies” – to treat everyone with CCP (“All CCP”) and to treat no one with CCP (“No CCP”). The results are given in eTable 3. A smaller “value” indicates a good performance.

© 2022 Park H et al. *JAMA Network Open.*

## eTable 3. Results in Terms of Value From Cross-validation Based on 400 Splits Into Training Data Set and Validation Data Set

The treatment decision rules (TDR) are developed using these alternative methods for WHO scores at day 14, and the "value" of the associated treatment decision rules evaluated on validation sets.

| Method | Outcomes | | | | | | Outcomes | | | | | |
|---|---|---|---|---|---|---|---|---|---|---|---|---|
| | Day 14 WHO ≥ 7 | | | Day 28 WHO ≥ 7 | | | Day 14 mortality | | | Day 28 mortality | | |
| | TDR | All CCP | No CCP | TDR | All CCP | No CCP | TDR | All CCP | No CCP | TDR | All CCP | No CCP |
| Q-Learning, with original outcomes | 0.135 | 0.138 | 0.158 | 0.142 | 0.141 | 0.167 | 0.070 | 0.063 | 0.085 | 0.111 | 0.102 | 0.137 |
| Q-Learning, using residuals | 0.139 | 0.138 | 0.158 | 0.149 | 0.141 | 0.167 | 0.074 | 0.063 | 0.085 | 0.117 | 0.102 | 0.137 |
| OWL- Linear kernel | 0.131 | 0.138 | 0.158 | 0.140 | 0.141 | 0.167 | 0.068 | 0.063 | 0.085 | 0.108 | 0.102 | 0.137 |
| RWL- Linear kernel | 0.146 | 0.138 | 0.158 | 0.150 | 0.141 | 0.167 | 0.074 | 0.063 | 0.085 | 0.123 | 0.102 | 0.137 |
| RICWL-Fraction, with original outcomes, RBF kernel | 0.143 | 0.138 | 0.158 | 0.148 | 0.141 | 0.167 | 0.069 | 0.063 | 0.085 | 0.114 | 0.102 | 0.137 |
| RICWL-Fraction, using residuals, RBF kernel | 0.140 | 0.138 | 0.158 | 0.147 | 0.141 | 0.167 | 0.068 | 0.063 | 0.085 | 0.112 | 0.102 | 0.137 |
| RICWL-Fraction, with original outcomes, linear kernel | 0.142 | 0.138 | 0.158 | 0.148 | 0.141 | 0.167 | 0.072 | 0.063 | 0.085 | 0.116 | 0.102 | 0.137 |
| RICWL-Fraction, using residuals, linear kernel | 0.139 | 0.138 | 0.158 | 0.147 | 0.141 | 0.167 | 0.071 | 0.063 | 0.085 | 0.114 | 0.102 | 0.137 |
| RICWL-Cosine, with original outcomes, RBF kernel | 0.143 | 0.138 | 0.158 | 0.149 | 0.141 | 0.167 | 0.068 | 0.063 | 0.085 | 0.112 | 0.102 | 0.137 |
| RICWL- Cosine, using residuals, RBF kernel | 0.140 | 0.138 | 0.158 | 0.146 | 0.141 | 0.167 | 0.067 | 0.063 | 0.085 | 0.111 | 0.102 | 0.137 |
| RICWL- Cosine, with original outcomes, linear kernel | 0.145 | 0.138 | 0.158 | 0.152 | 0.141 | 0.167 | 0.073 | 0.063 | 0.085 | 0.118 | 0.102 | 0.137 |
| RICWL- Cosine, using residuals, linear kernel | 0.138 | 0.138 | 0.158 | 0.147 | 0.141 | 0.167 | 0.070 | 0.063 | 0.085 | 0.114 | 0.102 | 0.137 |
| Boosting, using continuous outcomes | 0.142 | 0.138 | 0.158 | 0.149 | 0.141 | 0.167 | 0.073 | 0.063 | 0.085 | 0.115 | 0.102 | 0.137 |
| Boosting, using binary outcomes | 0.137 | 0.138 | 0.158 | 0.145 | 0.141 | 0.167 | 0.070 | 0.063 | 0.085 | 0.113 | 0.102 | 0.137 |

Some of the RBF kernel-based methods were not able to be computed due to singularities in the estimation. The computation time for the support vector machine-based methods (i.e., RWL/OWL/RICWL) was about 3 hours for each run; this is in contrast to the computation time of a TBI model which is only a few seconds. The variables that were used in the above alternative methods were: age, sex, baseline WHO score, blood type, and the indicators of cardiovascular disease, diabetes, and pulmonary disease; this set of variables corresponds to the set of variables used in the index "P" specified in eTable 6. Thus, a direct comparison can be made between the results present in eTables 2 and 3 and the results in eTables 7 and 8 corresponding to the index "P". In eTables 2 and 3, the outcome-weighted learning (OWL) with linear kernel and boosting trained based on the binary outcome of WHO ≥ 7 performed superiorly among these alternative approaches considered. However, both of these approaches were clearly outperformed by the index "P" of the TBI models. This is expected because the TBI model (S1.1) efficiently utilized the ordinal structure of the response data, and given the noisy clinical data, the simplicity of the TBI model compared to the alternative approaches provided a superior prediction.

© 2022 Park H et al. *JAMA Network Open.*

## eAppendix 8. Baseline Characteristics of the Participants in the Study Used to Develop the TBIs

Altogether 2369 participants met trial eligibility in the main COMIPLE study[12]; 1138 were randomized to control and 1231 to CCP, and the participants' baseline characteristics are described in eTable 4.

[12] AB Troxel et al., Effect of COVID-19 Convalescent Plasma on Clinical Status in Hospitalized Patients: A Real-Time Individual Patient Data Meta-Analysis of Randomized Clinical Trials, *JAMA Network Open*; 2022;5(1):e2147331. doi:10.1001/jamanetworkopen.2021.47331

© 2022 Park H et al. *JAMA Network Open.*

eTable 4. Baseline Characteristics of the Participants in the COMPILE Study by Treatments

| Baseline characteristics | Control | CCP | Overall |
|---|---|---|---|
| | n=1138 | n=1231 | n=2369 |
| Age (median, IQR) | 60 [50, 72] | 61 [50, 71] | 60 [50, 72] |
| Sex | | | |
| Female (n, %) | 408 (35.9) | 437 (35.5) | 845 (35.7) |
| Male (n, %) | 730 (64.1) | 794 (64.5) | 1524 (64.3) |
| Baseline WHO severity (n, %) | | | |
| 4 - hospitalized/ no O2 | 235 (20.7) | 217 (17.6) | 452 (19.1) |
| 5 - hospitalized/ O2 by mask or nasal | 701 (61.6) | 800 (65.0) | 1501 (63.4) |
| 6 - hospitalized/ O2 by non-invasive ventilation | 202 (17.8) | 214 (17.4) | 416 (17.6) |
| Blood group (n, %) | | | |
| O | 518 (45.5) | 568 (46.1) | 1086 (45.8) |
| A | 374 (32.9) | 420 (34.1) | 794 (33.5) |
| B | 195 (17.1) | 181 (14.7) | 376 (15.9) |
| AB | 38 ( 3.3) | 54 ( 4.4) | 92 ( 3.9) |
| Not available | 13 ( 1.1) | 8 ( 0.6) | 21 ( 0.9) |
| Days since symptoms onset (n, %) | | | |
| 0-3 days | 142 (12.5) | 148 (12.0) | 290 (12.2) |
| 4-6 days | 394 (34.6) | 441 (35.8) | 835 (35.2) |
| 7-10 days | 402 (35.3) | 431 (35.0) | 833 (35.2) |
| 11-14 days | 136 (12.0) | 125 (10.2) | 261 (11.0) |
| >14 days | 58 ( 5.1) | 74 ( 6.0) | 132 ( 5.6) |
| NA | 6 ( 0.5) | 12 ( 1.0) | 18 ( 0.8) |
| Days since COVID-19 diagnosis at randomization (median, IQR) | 2 [1, 3] | 1 [1, 3] | 2 [1, 3] |
| History of diabetes (n, %) | | | |
| No | 770 (67.7) | 804 (65.3) | 1574 (66.4) |
| Yes | 368 (32.3) | 427 (34.7) | 795 (33.6) |
| NA | 0 (0.0) | 0 (0.0) | 0 (0.0) |
| History of pulmonary disease (n, %) | | | |
| No | 998 (87.7) | 1082 (87.9) | 2080 (87.8) |
| Yes | 136 (12.0) | 144 (11.7) | 280 (11.8) |
| NA | 4 ( 0.4) | 5 ( 0.4) | 9 ( 0.4) |
| History of cardiovascular disease (n, %) | | | |
| No | 660 (58.0) | 694 (56.4) | 1354 (57.2) |
| Yes | 474 (41.7) | 534 (43.4) | 1008 (42.5) |
| NA | 4 ( 0.4) | 3 ( 0.2) | 7 ( 0.3) |
| Enrollment quarter (n, %) | | | |
| Apr-June 2020 | 344 (30.2) | 297 (24.1) | 641 (27.1) |
| July-Sept 2020 | 215 (18.9) | 242 (19.7) | 457 (19.3) |
| Oct-Dec 2020 | 405 (35.6) | 504 (40.9) | 909 (38.4) |
| Jan-Mar 2021 | 174 (15.3) | 188 (15.3) | 362 (15.3) |

The TBI was developed on complete cases (n=2287). eFigure 3 below provides the marginal and joint distributions of the missingness (n=82) in the key baseline characteristics.

© 2022 Park H et al. *JAMA Network Open.*

## eFigure 3. Missingness for Each of the Key Baseline Characteristics and Outcomes and Each Combination of the Variables for the 82 Participants Dropped From the Complete Case Analysis

(Enrolledqr: Enrollment quarter; Blood: blood type; Diabetes: a history of diabetes; Pulm: a history of pulmonary disease; Cardio: a history of cardiovascular disease; SympDays: Days since symptoms onset; WHO.14: WHO-11 scale outcome at day 14; WHO.28: WHO-11 scale outcome at day 28.)

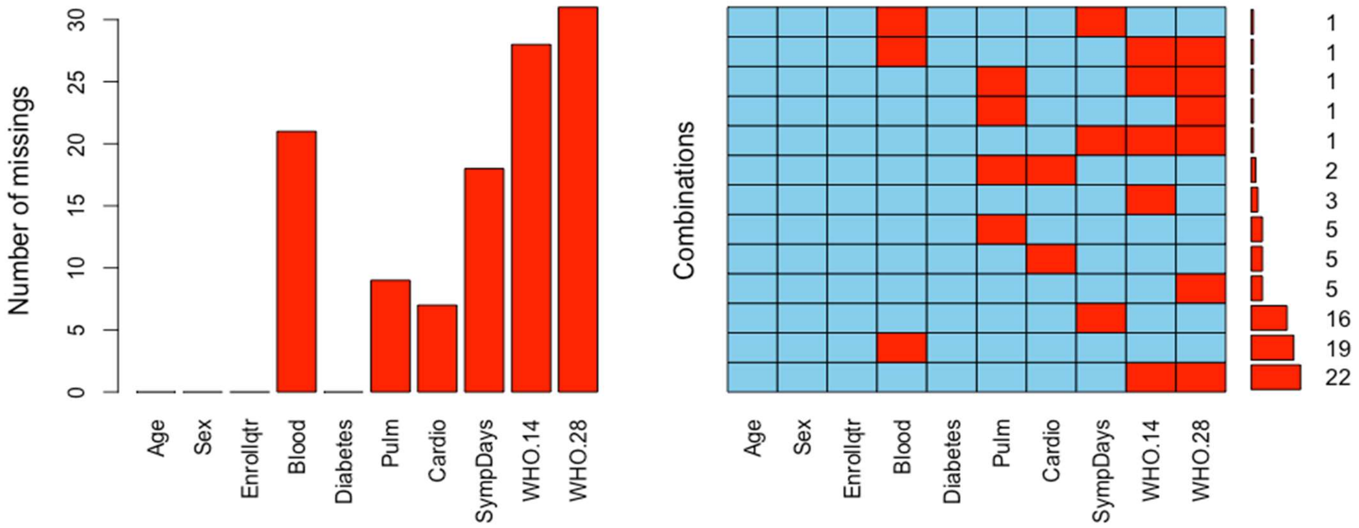


© 2022 Park H et al. *JAMA Network Open.*

## eAppendix 9. Main Effect Model

Selection of the covariates in the main effect $\mu(\boldsymbol{Z})$ of the POM (S1.1 in eAppendix 1) for day 14 WHO score was based on AIC, see eTable 5 for the selected covariates used for the development of all TBIs, elicited in Part I of the workflow diagram on eFigure 1. The model also contains RCT-specific random intercept effects, which were highly significant. The initial model used all baseline covariates existing in the COMPILE study, including (i) age, (ii) sex, (iii) baseline WHO score, (iv) duration of symptoms (in bins of 3 or 4 days), (v) number of days from diagnosis to randomization, (vi) days from randomization to first infusion, (vii) blood type, (viii) diabetes, (ix) cardiovascular disease, (x) pulmonary disease, (xi) quarter of enrollment.

## eTable 5. Main Effects Model

Model 1 is the initial model that contained the main effects of all available baseline predictors in the COMPILE study in their original form. The Selected main effect model contains transformations of the original variables and interactions between them.

| | Model 1 | | | | **Selected main effect model** | | | |
|---|---|---|---|---|---|---|---|---|
| Coefficient | Estimate | St. Err. | z value | Pr(>\|z\|) | Estimate | St. Err. | z value | Pr(>\|z\|) |
| Intercept | -1.64 | 0.40 | -4.054 | 5.19e-05 *** | -0.18 | 0.62 | -0.293 | 0.7697 |
| Age | 0.03 | 0.003 | 11.626 | < 2e-16 *** | -0.0108 | 0.0164 | -0.656 | 0.0027 ** |
| $Age^2$ | | | | | 0.0003 | 0.0001 | 1.992 | 0.0428 * |
| Sex Male | 0[1] | | | | 0 | | | |
| Sex Female | -0.09 | 0.08 | -1.273 | 0.2212 | -0.08 | 0.07 | -1.057 | 0.2906 |
| Baseline WHO = 4 | 0 | | | | 0 | | | |
| Baseline WHO = 5 | 0.92 | 0.14 | 6.779 | 1.26e-11 *** | 0.36 | 0.43 | 0.842 | 0.3995 |
| Baseline WHO = 6 | 2.46 | 0.17 | 14.689 | < 2e-16 *** | 0.42 | 0.55 | 0.773 | 0.4397 |
| Baseline WHO = 5 & age | | | | | 0.0099 | 0.0070 | 1.415 | 0.1571 |
| Baseline WHO = 6 & age | | | | | 0.0334 | 0.0086 | 3.848 | 0.0001 *** |
| Blood type O | 0 | | | | | | | |
| Blood type A | 0.14 | 0.08 | 1.686 | 0.0849 | | | | |
| Blood type B | -0.002 | 0.11 | 0.002 | 0.9877 | | | | |
| Blood type AB | 0.17 | 0.20 | 0.893 | 0.3728 | | | | |
| Blood type O or B | | | | | 0 | | | |
| Blood type A or AB | | | | | 0.14 | 0.08 | 1.786 | 0.0767 |
| History of DM | 0.10 | 0.08 | 1.317 | 0.2095 | | | | |
| History of CVD | 0.06 | 0.09 | 0.754 | 0.4654 | | | | |
| History of PD | 0.09 | 0.12 | 0.823 | 0.4298 | | | | |
| Comorbid DM&CVD | | | | | 0.16 | 0.10 | 1.640 | 0.1009 |
| Comorbid DM&PD | | | | | 0.25 | 0.19 | 1.347 | 0.1778 |
| Duration of symptoms 0-3 days | 0 | | | | | | | |
| Duration of symptoms 4-6 days | 0.03 | 0.12 | 0.244 | 0.8293 | | | | |
| Duration of symptoms 7-10 days | -0.14 | 0.13 | -1.152 | 0.2460 | | | | |
| Duration of symptoms 11-14 days | -0.16 | 0.16 | -1.060 | 0.2944 | | | | |
| Duration of symptoms >14 days | -0.01 | 0.20 | -0.087 | 0.9496 | | | | |
| Duration of symptoms ≥ 7 days | | | | | 0 | | | |
| Duration of symptoms 0-6 days | | | | | 0.14 | 0.08 | 1.855 | 0.0635 |
| Enrollment April – June 2020 | 0 | | | | | | | |
| Enrollment July – September 2020 | -0.37 | 0.12 | -3.094 | 0.0019 ** | -0.34 | 0.12 | -2.815 | 0.0048 ** |
| Enrollment Oct. – Dec. 2020 | -0.28 | 0.12 | -2.416 | 0.0156 * | -0.26 | 0.12 | -2.242 | 0.0249 * |
| Enrollment January – March 2021 | -0.38 | 0.14 | -2.754 | 0.0055 ** | -0.34 | 0.14 | 2.509 | 0.0120 * |
| AIC | 8806.056 | | | | 8776.527 | | | |

[1] A coefficient equal to 0 indicates that the level of the categorical outcome is the reference category

© 2022 Park H et al. *JAMA Network Open.*

## eAppendix 10. Patient Features Considered for Inclusion in the TBI

eTable 6 presents the set of covariates in the indices considered. The variables in the candidate TBI models included categorization of continuous features (e.g., age, with the dichotomizing cutoff 67, which was selected based on a CV), collapsing of multi-category variables into factors with fewer levels (e.g., duration of symptoms prior to randomization) and interactions between the variables.

© 2022 Park H et al. *JAMA Network Open.*

## eTable 6. Candidate Sets of Features Considered for Inclusion in the TBI

This is the result of process II in the "Development of the TBI model" in the workflow diagram in eFigure 1.

| | |
|---|---|
| Index A | Age, baseline WHO score, blood type[1], CVD[3], DM[4], PD[5] |
| Index B | Baseline WHO score, blood type[1], CVD, DM, PD |
| Index C | Age, baseline WHO score, blood type[2], CVD, DM, PD |
| Index D | Baseline WHO score, blood type[2], CVD, DM, PD |
| Index E | Age, baseline WHO score, blood type[2], CVD, DM |
| Index F | Baseline WHO score, blood type[2], CVD, DM |
| Index G | Baseline WHO score, CVD, DM, PD |
| Index H | Baseline WHO score, CVD, DM |
| Index I | Age, Baseline WHO score, CVD, DM |
| Index J | Baseline WHO score, blood type[2], CVD, DM&PD[6] |
| Index K | Baseline WHO score, blood type[2], CVD, DM&CVD[7], DM&PD |
| Index L | Baseline WHO score, blood type[2], CVD, DM, DM&CVD, DM&PD |
| Index M | Baseline WHO score, blood type[2], CVD, DM, DM&CVD |
| Index N | Baseline WHO score, CVD, DM&CVD, DM&PD |
| Index O | Baseline WHO score, blood type[2], CVD, DM, PD, DM&CVD, DM&PD |
| Index P | Age, Sex, Baseline WHO score, blood type[1], CVD, DM, PD |
| Index Q | Age, Sex, Baseline WHO score, blood type[2], CVD, DM, PD |
| Index R | Baseline WHO score, blood type[2], CVD |
| Index S | Age[8], Baseline WHO score, Baseline WHO score&Age[8], blood type[2], CVD, DM&PD |
| Index T | Age[8], Baseline WHO score, Baseline WHO score&Age[8], CVD, DM&PD |
| Index U | Age[8], Baseline WHO score, blood type[2], CVD |
| Index V | Age, Baseline WHO score, Baseline WHO score&Age, blood type[2], CVD, DM&PD |
| **Index "Basic"** | **Baseline WHO score, Baseline WHO score&Age[8], CVD, DM&PD** |
| **Index "Expanded"** | **Baseline WHO score, Baseline WHO score&Age[8], blood type[2], CVD, DM&PD** |

[1] Blood type has 4 levels – O, A, B, AB;
[2] Blood type has 2 levels – (A or AB) and (O or B)
[3] CVD=cardiovascular disease
[4] DM=Diabetes Mellitus
[5] PD=pulmonary disease
[6] DM&PD = comorbid DM and PD
[7] DM&CVD = comorbid DM and CVD
[8] Age= Age dichotomized (1 if Age ≥ 67, 0 otherwise)

© 2022 Park H et al. *JAMA Network Open.*

## eAppendix 11. Results From 1000 Split-Sample-Simulation Cross-validation

Evaluation of TBI with respect to the differential benefit in groups B and NB

CV, using random partitioning of the data, derivation of classifiers in one part ($2/3^{rds}$) of the data, with subsequent test of the classifier in the remaining part ($1/3^{rd}$) of the data, was conducted on all TBIs in eTable3.3, and the results are shown in eTable 7 (in the rows A-V, "Basic" and "Expanded"). The last row of the table corresponds to the results for the case where we select and estimate a TBI, rather than using an already specified model (e.g., Index A, Index B, Index C, …) to do the CV. In eTable 7 below, the entries are the medians over the 1000 random splits used for CV, where the performance is evaluated on the respective ($1/3^{rd}$) test sets. In the table, the columns labeled as "OR(B)" show the median of the CCP efficacy ORs in the group predicted to benefit (B) from CCP; columns labeled as "OR(NB)" show those in the group not predicted to benefit (NB) from CCP. Columns "B/NB" show the median of the ratios, OR(B)/OR(NB). Smaller values in columns "B" and "B/NB" correspond to better TBI performance. Looking at the results across all six outcomes, the TBIs "Expanded", J, S and V appear to have the best, or close to best, CV performance among all candidate TBIs. Also, considering that the patient's blood type information may not be readily available when clinicians make treatment decisions, we considered the TBIs that do not require the patient's blood type information (i.e., G, H, I, N, and "Basic"). Among these, the TBI "Basic" has the best performance.

In eTable 7, the last row reports the CV result for the case where we estimate a TBI including patient's feature selection, rather than using an already specified set of features (e.g., Index A, Index B, Index C, …) to do the CV. Specifically, given a training set (which corresponds to the $2/3^{rds}$ of the whole sample), we further split this $2/3^{rds}$ training set into: 1) the $2/3^{rds}$ "development" set (i.e., the $2/3^{rds} * 2/3^{rds}$ of the whole sample), and 2) the $1/3^{rd}$ "validation" set (i.e., the $2/3^{rds} * 1/3^{rd}$ of the whole sample). Then we conducted the CV within the training set (i.e., derivation of TBIs on the "development" set, and evaluation of their performance on the "validation" set), followed by ranking the TBIs according to the performance on the "validation set". Then, we selected the best-performing TBI (say, Index A), and re-estimated this selected TBI on the training set (which corresponds to the $2/3^{rds}$ of the whole sample, specified at the beginning). We then evaluated this trained TBI on the testing set (which corresponds to the $1/3^{rd}$ of the whole sample, set at the beginning). This CV procedure including the model selection procedure was repeated over 1000 times, and the median results are reported in the last row of eTable 7.

eFigure 4 below displays the distribution of the odds ratios, OR(B) and OR(NB), associated with the last row of eTable 7 from the 1000 split-sample simulation CV, with respect to each of the 6 outcomes considered. There is a reasonably clear separation in the CCP efficacy odds ratio between B and NB, especially with respect to the outcome of being on mechanical ventilator or worse (WHO $\geq$ 7) (the second column), suggesting that this development approach provides a reasonably well-performing TBI. (The OR distributions obtained from this 1000 split-sample simulation CV for the "Expanded" TBI are provided in Section eAppendix 30 of this document.)

Evaluation of TBI with respect to "value"

As described in eAppendix 2, we also compared candidate TBIs with respect to their "value". A "value" of a treatment classification rule is the expected value of the outcome if every patient is treated according to the given treatment classification rule. This expected "value" is computed with respect to the binary outcomes (i.e., the indicator of WHO$\geq$7 and the indicator of WHO=10 at days 14 and 28), and it indicates the incidence of those clinically undesirable outcomes when the given treatment classification rule is applied to the patient population. eTable 8 (in columns "TBI") reports the median of the values under the TBI-based treatment classification rules considered in eTable 6, obtained from the 1000 split-sample simulation CV. These values

are compared with the medians of the values of two one-size-fits-all treatment policies -- to treat everyone with CCP (in columns "All CCP") and to treat no one with CCP (in columns "No CCP").

As in eTable 7, the last row in eTable 8 reports the CV results for the case where the patient's feature selection procedure is incorporated: for each cross-validation run, the 2/3$^{rds}$ training set is further split into 2/3$^{rds}$ of the "development" and 1/3$^{rd}$ of the "validation" sets, i.e., 4/9 of the whole sample was used to train the model, and 2/9 of the whole sample was used to validate the model; then use the model with the smallest B/NB ratio (with respect to the ordinal outcome at day 14), then re-fit the selected model on the 2/3$^{rds}$ initial training set and evaluate the fitted model on the initial 1/3$^{rd}$ testing set.

Several observations can be made from eTable 8. First, while prevalence of WHO≥7 at day 14 under CCP treatment was 13.6% (see the 3$^{rd}$ column, "All CCP"), under control treatment it is 15.9%, for a difference of 2.3%. Every one of the TBIs had better (i.e., lower) "value" than, not only the policy to treat no one with CCP, but also the policy to treat everyone with CCP. The difference between the values of the "All CCP" policy and the values for all TBIs are small and varying, with the improvement from the TDR based on the "Expanded" TBI equaling 1.2% (comparing the 2$^{nd}$ and 3$^{rd}$ columns). Second, similar conclusions can be made with respect to the outcome WHO≥7 at day 28, where the rate of WHO≥7 under CCP treatment is 14.1%, under control treatment is 16.8% for a difference of 2.7%. In this case, the improvement with respect to value if the TDR based on the "Expanded" TBI is implemented instead of treating everyone with CCP, is (0.141-0.127=) 1.4%, i.e., half of the average effect of CCP vs. control, which is 2.7% WHO≥0 at day 28. This illustrates how, by targeted use of CCP, improvement can be obtained that is half as large in magnitude, as that of the improvement of treating everyone with CCP vs. not treating anyone with CCP. Third, with respect to mortality, both at days 14 and 28, none of the TDRs derived from the considered TBIs resulted in better value than the policy of treating everyone with CCP, or the improvement was miniscule (see mortality at day 28, for the "Expanded" TBI), where an improvement of only 0.1% is achieved, see the 2$^{nd}$ and 3$^{rd}$ columns from the right in the last row of eTable 8. These observations, together with results Figures 1 and 2 in the main manuscript, suggest that CCP treatment might be harmful for some patients in B3 with respect to the status of being on ventilation or worse (WHO≥7) at days 14 and 28, but with respect to mortality, CCP is safe with no evidence for harm.

The results in eTables 7 and 8 indicate that the "Expanded" TBI outperformed the rest of the candidate indices in the majority of the cases, in terms of its ability to separate the groups B and NB in a way that makes the outcomes in those groups most different, as well as with respect to value of the treatment classification rules. In terms of performance, the "Expanded" TBI is followed by other candidate indices (J, S and V in eTable 6). Among those not involving blood type information, the "Basic" TBI had superior performance. This process -- part II from the workflow diagram on eFigure 1 -- reduced the number of strong candidate TBIs to 5: indices J, S and V, "Basic" and "Expanded".

© 2022 Park H et al. *JAMA Network Open.*

# eTable 7. Results From 1000 Split-Sample Simulation Cross-validation

Based on a TBI developed on the training set (using the POM (S1.1 in eAppendix 1) for WHO scores at day 14), each hold-out set is stratified into a group expected to benefit (B) and a group not expected to benefit (NB) from CCP. For all six outcomes, the CCP efficacy ORs are computed within the subgroups B and NB of the holdout set, and the ratio of these, ratio of ORs, OR(B)/OR(NB), are computed; a smaller (and OR(B)/OR(NB)< 1) ratio indicates a good TBI model. The entries show the median of the ORs across the 1000 splits. Index refers to the indices in eTable 5.

| Index | Outcomes | | | | | | | | | | | | | | | | | |
|---|---|---|---|---|---|---|---|---|---|---|---|---|---|---|---|---|---|---|
| | Day 14 WHO ordinal | | | Day 28 WHO ordinal | | | Day 14 WHO ≥ 7 | | | Day 28 WHO ≥ 7 | | | Day 14 mortality | | | Day 28 mortality | | |
| | OR(B) | OR(NB) | B/NB | OR(B) | OR(NB) | B/NB | OR(B) | OR(NB) | B/NB | OR(B) | OR(NB) | B/NB | OR(B) | OR(NB) | B/NB | OR(B) | OR(NB) | B/NB |
| A | 0.825 | 0.982 | 0.836 | 0.832 | 1.071 | 0.771 | 0.668 | 1.176 | 0.560 | 0.678 | 1.098 | 0.611 | 0.649 | 0.812 | 0.762 | 0.634 | 0.883 | 0.710 |
| B | 0.822 | 0.991 | 0.817 | 0.832 | 1.077 | 0.769 | 0.659 | 1.182 | 0.541 | 0.665 | 1.095 | 0.604 | 0.638 | 0.821 | 0.751 | 0.622 | 0.887 | 0.696 |
| C | 0.833 | 0.972 | 0.850 | 0.837 | 1.053 | 0.783 | 0.681 | 1.149 | 0.585 | 0.678 | 1.059 | 0.63 | 0.643 | 0.823 | 0.748 | 0.636 | 0.868 | 0.701 |
| D | 0.830 | 0.985 | 0.825 | 0.842 | 1.062 | 0.783 | 0.673 | 1.159 | 0.572 | 0.674 | 1.083 | 0.608 | 0.644 | 0.825 | 0.741 | 0.628 | 0.895 | 0.680 |
| E | 0.833 | 0.964 | 0.854 | 0.833 | 1.051 | 0.780 | 0.645 | 1.172 | 0.536 | 0.648 | 1.118 | 0.564 | 0.607 | 0.884 | 0.675 | 0.601 | 0.925 | 0.635 |
| F | 0.835 | 0.973 | 0.841 | 0.841 | 1.055 | 0.778 | 0.639 | 1.196 | 0.527 | 0.643 | 1.140 | 0.543 | 0.599 | 0.909 | 0.639 | 0.589 | 0.940 | 0.615 |
| G | 0.850 | 0.933 | 0.898 | 0.857 | 0.991 | 0.829 | 0.706 | 1.014 | 0.673 | 0.694 | 0.978 | 0.692 | 0.627 | 0.845 | 0.698 | 0.626 | 0.849 | 0.718 |
| H | 0.846 | 0.939 | 0.878 | 0.854 | 1.004 | 0.815 | 0.691 | 1.045 | 0.639 | 0.680 | 1.016 | 0.642 | 0.616 | 0.880 | 0.671 | 0.614 | 0.876 | 0.658 |
| I | 0.852 | 0.927 | 0.892 | 0.853 | 0.995 | 0.829 | 0.689 | 1.023 | 0.648 | 0.688 | 0.991 | 0.666 | 0.624 | 0.836 | 0.714 | 0.623 | 0.867 | 0.694 |
| J | 0.800 | 1.036 | 0.753 | 0.837 | 1.085 | 0.762 | 0.646 | 1.215 | 0.509 | 0.624 | 1.232 | 0.487 | 0.564 | 1.006 | 0.530 | 0.568 | 1.032 | 0.524 |
| K | 0.804 | 1.006 | 0.784 | 0.825 | 1.064 | 0.776 | 0.589 | 1.233 | 0.459 | 0.589 | 1.178 | 0.487 | 0.560 | 0.915 | 0.599 | 0.542 | 0.985 | 0.540 |
| L | 0.813 | 0.986 | 0.806 | 0.828 | 1.051 | 0.784 | 0.590 | 1.227 | 0.478 | 0.597 | 1.169 | 0.504 | 0.565 | 0.908 | 0.615 | 0.557 | 0.975 | 0.560 |
| M | 0.815 | 0.987 | 0.814 | 0.833 | 1.048 | 0.787 | 0.601 | 1.216 | 0.487 | 0.605 | 1.151 | 0.515 | 0.580 | 0.890 | 0.637 | 0.566 | 0.960 | 0.572 |
| N | 0.808 | 0.958 | 0.835 | 0.832 | 1.000 | 0.812 | 0.593 | 1.075 | 0.540 | 0.591 | 1.052 | 0.552 | 0.530 | 0.891 | 0.569 | 0.532 | 0.939 | 0.564 |
| O | 0.805 | 1.017 | 0.786 | 0.826 | 1.070 | 0.769 | 0.617 | 1.210 | 0.499 | 0.626 | 1.126 | 0.541 | 0.596 | 0.870 | 0.666 | 0.576 | 0.948 | 0.602 |
| P | 0.834 | 0.972 | 0.848 | 0.841 | 1.060 | 0.782 | 0.668 | 1.160 | 0.564 | 0.680 | 1.087 | 0.610 | 0.647 | 0.824 | 0.746 | 0.635 | 0.884 | 0.692 |
| Q | 0.839 | 0.963 | 0.863 | 0.840 | 1.050 | 0.788 | 0.679 | 1.143 | 0.590 | 0.684 | 1.067 | 0.643 | 0.643 | 0.818 | 0.777 | 0.637 | 0.870 | 0.714 |
| R | 0.797 | 1.069 | 0.736 | 0.836 | 1.104 | 0.743 | 0.657 | 1.220 | 0.520 | 0.645 | 1.200 | 0.506 | 0.591 | 0.957 | 0.590 | 0.597 | 1.002 | 0.569 |
| S | 0.805 | 1.020 | 0.766 | 0.831 | 1.069 | 0.757 | 0.601 | 1.215 | 0.484 | 0.581 | 1.205 | 0.459 | 0.556 | 0.934 | 0.575 | 0.550 | 0.978 | 0.534 |
| T | 0.815 | 0.993 | 0.814 | 0.835 | 1.041 | 0.792 | 0.674 | 1.065 | 0.616 | 0.633 | 1.100 | 0.550 | 0.566 | 0.924 | 0.586 | 0.568 | 0.944 | 0.579 |
| U | 0.792 | 1.059 | 0.730 | 0.832 | 1.101 | 0.747 | 0.629 | 1.200 | 0.508 | 0.625 | 1.171 | 0.500 | 0.589 | 0.908 | 0.629 | 0.588 | 0.949 | 0.603 |
| V | 0.799 | 1.018 | 0.764 | 0.840 | 1.063 | 0.775 | 0.625 | 1.177 | 0.506 | 0.591 | 1.196 | 0.465 | 0.552 | 0.932 | 0.566 | 0.549 | 1.003 | 0.516 |
| “Basic” | 0.802 | 1.019 | 0.775 | 0.825 | 1.067 | 0.772 | 0.664 | 1.076 | 0.597 | 0.626 | 1.135 | 0.527 | 0.560 | 0.942 | 0.568 | 0.561 | 0.969 | 0.562 |
| “Expanded” | 0.787 | 1.062 | 0.724 | 0.820 | 1.103 | 0.726 | 0.597 | 1.231 | 0.473 | 0.575 | 1.240 | 0.448 | 0.546 | 0.959 | 0.568 | 0.541 | 1.014 | 0.522 |
| | 0.825 | 0.992 | 0.812 | 0.842 | 1.060 | 0.780 | 0.655 | 1.147 | 0.542 | 0.649 | 1.126 | 0.554 | 0.609 | 0.882 | 0.663 | 0.606 | 0.932 | 0.630 |

© 2022 Park H et al. *JAMA Network Open.*

# eFigure 4. Subgroup-Specific OR Distributions Obtained From a 1000 Split-Sample Simulation Cross-validation

Evaluated on the respective test sets, corresponding the last row of eTable 7 (i.e., for the case where we included feature selection in estimating a TBI).

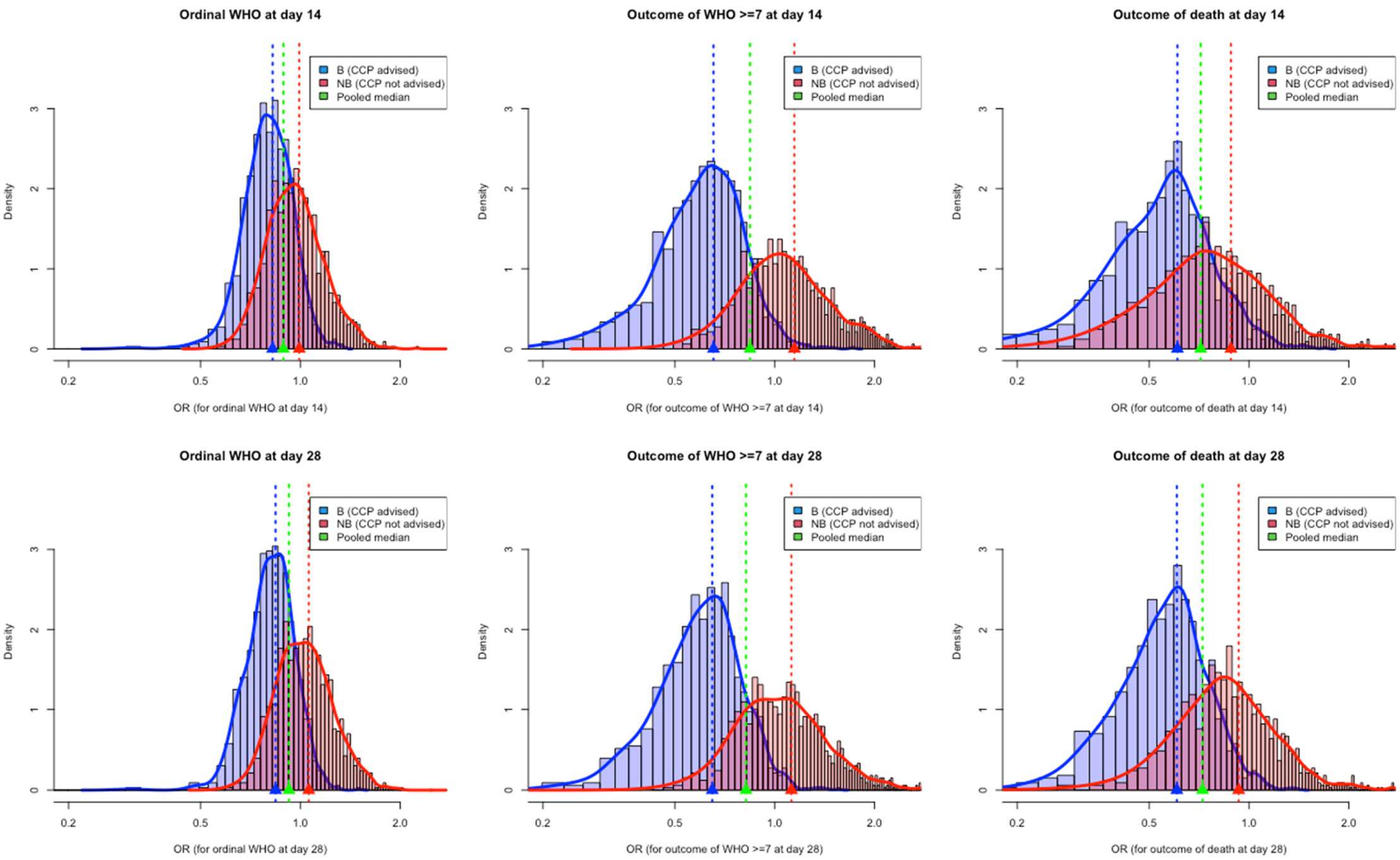


© 2022 Park H et al. *JAMA Network Open.*

## eTable 8. Value Results From the 1000 Split-Sample Simulation Cross-validation

Based on a TBI developed on the training set (using the POM (S1.1 in eAppendix 1) for WHO scores at day 14), the expected values of the four binary outcomes under the TBI-based treatment classification rules (in column "TBI") and those under two "treatment policies" – to treat everyone with CCP (in column "All CCP") and to treat no one with CCP (in column "No CCP")— are evaluated on each testing set; small values are desired. The entries (in columns TBI, All CCP or No CCP) show the median of the estimated expected values across the 1000 splits. Index refers to the models in eTable 5.

| Index | Outcomes | | | | | | | | | | | |
|---|---|---|---|---|---|---|---|---|---|---|---|---|
| | Day 14 WHO ≥ 7 | | | Day 28 WHO ≥ 7 | | | Day 14 mortality | | | Day 28 mortality | | |
| | TBI | All CCP | No CCP | TBI | All CCP | No CCP | TBI | All CCP | No CCP | TBI | All CCP | No CCP |
| A | 0.129 | 0.136 | 0.159 | 0.137 | 0.141 | 0.168 | 0.067 | 0.062 | 0.086 | 0.108 | 0.102 | 0.137 |
| B | 0.128 | 0.136 | 0.159 | 0.137 | 0.141 | 0.168 | 0.067 | 0.062 | 0.086 | 0.107 | 0.102 | 0.137 |
| C | 0.130 | 0.136 | 0.159 | 0.138 | 0.141 | 0.168 | 0.067 | 0.062 | 0.086 | 0.108 | 0.102 | 0.137 |
| D | 0.129 | 0.136 | 0.159 | 0.137 | 0.141 | 0.168 | 0.067 | 0.062 | 0.086 | 0.107 | 0.102 | 0.137 |
| E | 0.129 | 0.136 | 0.159 | 0.135 | 0.141 | 0.168 | 0.066 | 0.062 | 0.086 | 0.106 | 0.102 | 0.137 |
| F | 0.128 | 0.136 | 0.159 | 0.134 | 0.141 | 0.168 | 0.065 | 0.062 | 0.086 | 0.104 | 0.102 | 0.137 |
| G | 0.136 | 0.136 | 0.159 | 0.142 | 0.141 | 0.168 | 0.068 | 0.062 | 0.086 | 0.110 | 0.102 | 0.137 |
| H | 0.134 | 0.136 | 0.159 | 0.141 | 0.141 | 0.168 | 0.067 | 0.062 | 0.086 | 0.108 | 0.102 | 0.137 |
| I | 0.136 | 0.136 | 0.159 | 0.142 | 0.141 | 0.168 | 0.068 | 0.062 | 0.086 | 0.110 | 0.102 | 0.137 |
| J | 0.126 | 0.136 | 0.159 | 0.131 | 0.141 | 0.168 | 0.062 | 0.062 | 0.086 | 0.101 | 0.102 | 0.137 |
| K | 0.123 | 0.136 | 0.159 | 0.131 | 0.141 | 0.168 | 0.065 | 0.062 | 0.086 | 0.103 | 0.102 | 0.137 |
| L | 0.125 | 0.136 | 0.159 | 0.132 | 0.141 | 0.168 | 0.065 | 0.062 | 0.086 | 0.104 | 0.102 | 0.137 |
| M | 0.125 | 0.136 | 0.159 | 0.133 | 0.141 | 0.168 | 0.066 | 0.062 | 0.086 | 0.104 | 0.102 | 0.137 |
| N | 0.131 | 0.136 | 0.159 | 0.137 | 0.141 | 0.168 | 0.066 | 0.062 | 0.086 | 0.106 | 0.102 | 0.137 |
| O | 0.126 | 0.136 | 0.159 | 0.134 | 0.141 | 0.168 | 0.066 | 0.062 | 0.086 | 0.104 | 0.102 | 0.137 |
| P | 0.129 | 0.136 | 0.159 | 0.138 | 0.141 | 0.168 | 0.068 | 0.062 | 0.086 | 0.108 | 0.102 | 0.137 |
| Q | 0.130 | 0.136 | 0.159 | 0.138 | 0.141 | 0.168 | 0.068 | 0.062 | 0.086 | 0.108 | 0.102 | 0.137 |
| R | 0.127 | 0.136 | 0.159 | 0.132 | 0.141 | 0.168 | 0.063 | 0.062 | 0.086 | 0.102 | 0.102 | 0.137 |
| S | 0.125 | 0.136 | 0.159 | 0.128 | 0.141 | 0.168 | 0.064 | 0.062 | 0.086 | 0.102 | 0.102 | 0.137 |
| T | 0.132 | 0.136 | 0.159 | 0.134 | 0.141 | 0.168 | 0.064 | 0.062 | 0.086 | 0.103 | 0.102 | 0.137 |
| U | 0.126 | 0.136 | 0.159 | 0.131 | 0.141 | 0.168 | 0.065 | 0.062 | 0.086 | 0.103 | 0.102 | 0.137 |
| V | 0.127 | 0.136 | 0.159 | 0.130 | 0.141 | 0.168 | 0.064 | 0.062 | 0.086 | 0.102 | 0.102 | 0.137 |
| "Basic" | 0.131 | 0.136 | 0.159 | 0.133 | 0.141 | 0.168 | 0.064 | 0.062 | 0.086 | 0.103 | 0.102 | 0.137 |
| "Expanded" | 0.124 | 0.136 | 0.159 | 0.127 | 0.141 | 0.168 | 0.063 | 0.062 | 0.086 | 0.101 | 0.102 | 0.137 |
| | 0.128 | 0.136 | 0.159 | 0.135 | 0.141 | 0.168 | 0.066 | 0.062 | 0.086 | 0.106 | 0.102 | 0.137 |

© 2022 Park H et al. *JAMA Network Open.*

## eAppendix 12. Results From Leave-One-RCT-Out Cross-validation

The leave-one-RCT-out CV was performed, as described in eAppendix 4, where we use each of the 4 largest trials (AA with 940 subjects, EE with 477 subjects, KK with 381 subjects and CC with 350 subjects) and a combined trial of the remaining 4 small RCTs as a testing set, forming 5 non-overlapping testing sets. Given each testing RCT, the remaining RCTs were used as a training set. Based on a TBI developed on the training set, the CCP efficacy ORs (and their ratios) for all six outcomes and the expected "values" with respect to the four binary outcomes were evaluated on the testing RCT. We did this for all 5 non-overlapping testing sets, and the results were averaged across these 5 testing sets. In eTable 9, the differential CCP efficacy, as judged by OR(B)/OR(NB) < 1, for all of the identified TBIs from the part II from the workflow diagram (i.e., "Expanded", "Basic", J, S and V) increased the confidence of these TBIs. The results shown in eTable 10 for the "value" of the treatment classifiers are also consistent with those of eTable 8 obtained from the 1000 split-sample simulation CV.

As in eTable 7 and 8, the last row of eTables 9 and 10 report the CV results for the case where we estimated a TBI including feature selection (from the 24 index sets considered). To be specific, in the leave-one-RCT-out CV, based on each training sample consist of four RCTs, we derived classifiers in three RCTs, with subsequent test of the classifier in the remaining one RCT. We did this for each RCT in the training sample. We then averaged the results, and ranked the TBIs and select a TBI. The selected TBI was then re-estimated on the training sample and then evaluated on the RCT initially held-out; we did this CV for all 5 RCTs one by one, and the CV results were averaged across the 5 held-out RCTs (reported in the last row of eTable 9 and 10). The results in the last rows indicate that the performance of TBIs is satisfactory.

© 2022 Park H et al. *JAMA Network Open.*

# eTable 9. Results From a Leave-One-RCT-Out Cross-validation

Each holdout set is stratified into the groups B and NB, based on a TBI developed on the training set (using the POM (S1.1 in eAppendix 1) for WHO scores at day 14). For all six outcomes, the CCP efficacy ORs are computed within B and NB of the (S1.1 in eAppendix 1) holdout set, and the ratio, OR(B)/OR(NB), are computed. The entries show the mean of the ORs (in columns "OR(B)" or "OR(NB)") or of the ratio of the ORs (in columns "B/NB") across the 5 holdout RCTs.

| Index | Outcomes | | | | | | | | | | | | | | | | | |
|---|---|---|---|---|---|---|---|---|---|---|---|---|---|---|---|---|---|---|
| | Day 14 WHO ordinal | | | Day 28 WHO ordinal | | | Day 14 WHO ≥ 7 | | | Day 28 WHO ≥ 7 | | | Day 14 mortality | | | Day 28 mortality | | |
| | OR(B) | OR(NB) | B/NB | OR(B) | OR(NB) | B/NB | OR(B) | OR(NB) | B/NB | OR(B) | OR(NB) | B/NB | OR(B) | OR(NB) | B/NB | OR(B) | OR(NB) | B/NB |
| J | 0.813 | 1.457 | 0.666 | 0.782 | 1.447 | 0.635 | 0.666 | 1.190 | 0.579 | 0.647 | 1.748 | 0.487 | 0.007 | 0.851 | 0.539 | 0.612 | 1.151 | 0.548 |
| S | 0.801 | 1.128 | 0.720 | 0.782 | 1.158 | 0.714 | 0.607 | 2.217 | 0.574 | 0.603 | 2.025 | 0.496 | 0.659 | 1.353 | 0.604 | 0.557 | 1.596 | 0.387 |
| V | 0.845 | 0.952 | 0.925 | 0.822 | 0.995 | 0.896 | 0.639 | 1.425 | 0.698 | 0.635 | 1.318 | 0.618 | 0.644 | 1.016 | 0.644 | 0.592 | 1.181 | 0.531 |
| **"Basic"** | **0.773** | **1.289** | **0.663** | **0.738** | **1.247** | **0.654** | **0.595** | **2.016** | **0.595** | **0.573** | **1.495** | **0.470** | **0.610** | **1.599** | **0.422** | **0.514** | **1.558** | **0.308** |
| **"Expanded"** | **0.791** | **1.387** | **0.663** | **0.730** | **1.468** | **0.571** | **0.546** | **1.903** | **0.328** | **0.580** | **1.622** | **0.422** | **0.625** | **0.818** | **1.180** | **0.584** | **1.076** | **0.366** |
| | **0.820** | **1.055** | **0.792** | **0.717** | **1.144** | **0.640** | **0.555** | **1.907** | **0.404** | **0.567** | **1.850** | **0.456** | **0.682** | **1.460** | **1.231** | **0.606** | **1.592** | **1.595** |

© 2022 Park H et al. *JAMA Network Open.*

# eTable 10. Value Results a Leave-One-RCT-Out Cross-validation

The holdout set is stratified into the groups B and NB, based on a TBI developed on the training set (using the POM (S1.1 in eAppendix 1) for WHO scores at day 14). For the four binary outcomes, the expected values of outcomes under the TBI-based decision rules and those under two "treatment policies" – to treat everyone with CCP ("All CCP") and to treat no one with CCP ("No CCP") -- are estimated based on the holdout testing set; small value are desired. The entries show the mean estimated expected values (in columns TBI, All CCP or No CCP) of the binary outcomes across the 5 holdout RCTs.

| Index | Outcomes | | | | | | | | | | | |
|---|---|---|---|---|---|---|---|---|---|---|---|---|
| | Day 14 WHO ≥ 7 | | | Day 28 WHO ≥ 7 | | | Day 14 mortality | | | Day 28 mortality | | |
| | TBI | All CCP | No CCP | TBI | All CCP | No CCP | TBI | All CCP | No CCP | TBI | All CCP | No CCP |
| J | 0.116 | 0.129 | 0.151 | 0.119 | 0.130 | 0.157 | 0.059 | 0.063 | 0.078 | 0.092 | 0.097 | 0.129 |
| S | 0.115 | 0.129 | 0.151 | 0.118 | 0.130 | 0.157 | 0.059 | 0.063 | 0.078 | 0.092 | 0.097 | 0.129 |
| V | 0.122 | 0.129 | 0.151 | 0.125 | 0.130 | 0.157 | 0.061 | 0.063 | 0.078 | 0.096 | 0.097 | 0.129 |
| **"Basic"** | **0.115** | **0.129** | **0.151** | **0.117** | **0.130** | **0.157** | **0.057** | **0.063** | **0.078** | **0.088** | **0.097** | **0.129** |
| **"Expanded"** | **0.111** | **0.129** | **0.151** | **0.118** | **0.130** | **0.157** | **0.058** | **0.063** | **0.078** | **0.094** | **0.097** | **0.129** |
| | **0.112** | **0.129** | **0.151** | **0.118** | **0.130** | **0.157** | **0.060** | **0.063** | **0.078** | **0.095** | **0.097** | **0.129** |

© 2022 Park H et al. *JAMA Network Open.*

## eAppendix 13. Results From Leave-One-Enrollment-Quarter-Out Cross-validation

The observed heterogeneity of the effect of CCP over time (see eFigure 2), which is non-patient related heterogeneity, may affect the performance of the TBIs. To assess whether the TBI-based treatment classification rules demonstrated stability over time through CV, we used the 4 annual quarters covering the period from the beginning of April 2020 to the end of March 2021 (as described in Supplement eAppendix 5). The number of subjects in the 4 enrollment quarters was as follows: 641 in April-June 2020 (Q2); 457 in July-September 2020 (Q3); 909 in October-December 2021 (Q4); 362 in January-March 2021 (Q5). As in the previous subsection, we performed a leave-one-enrollment-quarter-out CV for the selected indices J, S, V, “Basic” and “Expanded”. The results with respect to the CCP efficacy ORs are shown in eTable 11.

For all of the indices and for all outcomes, we have OR(B)/OR(NB) < 1 (in the columns “B/NB”). This provides some strong evidence of internal validity with respect to time of treatment during the pandemic, and increases the confidence of the proposed TBIs. In eTable 12 below, we further analyze the performance reported in eTable 11, for the Basic and Expanded TBIs, which are the final selected TBIs (see the next subsection). In eTable 12, we display each quarter-specific CCP efficacy ORs associated with the two subgroups (B and NB) and their ratios before they were averaged across the four enrollment quarters.

The results in eTable 12 indicate that, except for one entry associated with the basic TBI’s ratio of ORs (OR(B)/OR(NB)) with respect to the outcome of WHO ≥7 at day 14 in the quarter Q5 (January – March 2021), which was slightly greater than 1 (1.037), the performance of both the Basic and Expanded TBIs remains good (OR(B)/OR(NB)<1) over each passing quarter. This provides internal support of validity of the TBIs with respect to time of treatment during the pandemic.

In eTable 13 below, we report the leave-one-enrollment-quarter-out CV results for the “values” of the treatment classification rules.

The results shown in eTable 13 for the “value” of the treatment classification rules are also consistent with those of eTable 8 obtained from the 1000 split-sample simulation CV. As in the previous subsection, the last rows of eTable 11 and eTable 13 report the CV results for the case where we estimate a TBI including model selection (from the 24 index sets considered; see eTable 6). To be specific, in the leave-one-enrollment-quarter-out CV, based on each training sample consist of three enrollment quarters, we derived classifiers in two enrollment quarters, with subsequent test of the classifier in the remaining one enrollment quarter. We did this for each quarter in the training sample. We then averaged the results, and ranked the TBIs and selected a TBI. The selected TBI was then re-estimated on the training sample and then evaluated on the enrollment quarter initially held-out; we did this CV for all 4 enrollment quarters one by one, and the CV results were averaged across the 4 held-out enrollment quarters (reported in the last rows of eTable 11 and eTable 13). The results in the last rows in the both eTable 11 and eTable 13 indicate that the performance of TBIs is satisfactory.

© 2022 Park H et al. *JAMA Network Open.*

# eTable 11. Results From a Leave-One-Enrollment-Quarter-Out Cross-validation

The holdout set is stratified into a group expected to benefit (B) and a group not expected to benefit (NB) from CCP, based on a TBI developed on the training set (using the POM (eAppendix 1) for WHO scores at day 14). For all six outcomes, the CCP efficacy ORs are computed within B and NB of the holdout set, and the ratio, OR(B)/OR(NB), are computed. The entries show the mean of the ORs (in columns “OR(B)” or “OR(NB)”) or of the ratio of the ORs (in columns “B/NB”) across the 4 holdout enrollment quarters.

| Index | Outcomes | | | | | | | | | | | | | | | | | |
|---|---|---|---|---|---|---|---|---|---|---|---|---|---|---|---|---|---|---|
| | Day 14 WHO ordinal | | | Day 28 WHO ordinal | | | Day 14 WHO ≥ 7 | | | Day 28 WHO ≥ 7 | | | Day 14 mortality | | | Day 28 mortality | | |
| | OR(B) | OR(NB) | B/NB | OR(B) | OR(NB) | B/NB | OR(B) | OR(NB) | B/NB | OR(B) | OR(NB) | B/NB | OR(B) | OR(NB) | B/NB | OR(B) | OR(NB) | B/NB |
| J | 0.806 | 1.308 | 0.672 | 0.828 | 1.280 | 0.718 | 0.796 | 1.393 | 0.552 | 0.759 | 1.459 | 0.510 | 0.778 | 1.654 | 0.570 | 0.702 | 1.628 | 0.563 |
| S | 0.760 | 1.289 | 0.628 | 0.798 | 1.254 | 0.700 | 0.671 | 1.442 | 0.442 | 0.608 | 1.628 | 0.372 | 0.626 | 1.589 | 0.486 | 0.586 | 1.769 | 0.467 |
| V | 0.774 | 1.401 | 0.574 | 0.802 | 1.381 | 0.642 | 0.68 | 1.684 | 0.387 | 0.608 | 1.931 | 0.320 | 0.648 | 1.146 | 0.493 | 0.590 | 1.189 | 0.417 |
| **“Basic”** | **0.798** | **1.180** | **0.697** | **0.811** | **1.169** | **0.716** | **0.734** | **1.180** | **0.590** | **0.637** | **1.296** | **0.480** | **0.500** | **1.728** | **0.424** | **0.542** | **1.403** | **0.473** |
| **“Expanded”** | **0.767** | **1.266** | **0.620** | **0.776** | **1.267** | **0.631** | **0.555** | **1.642** | **0.334** | **0.550** | **1.596** | **0.359** | **0.567** | **1.308** | **0.450** | **0.566** | **1.399** | **0.464** |
| | **0.814** | **1.441** | **0.604** | **0.819** | **1.484** | **0.613** | **0.778** | **1.744** | **0.427** | **0.745** | **1.791** | **0.414** | **0.781** | **>10** | **0.552** | **0.691** | **2.164** | **0.452** |

© 2022 Park H et al. *JAMA Network Open.*

## eTable 12. Results From a Leave-One-Enrollment-Quarter-Out Cross-validation, Before Averaging Across the 4 Enrollment Quarters (Q2-Q5) for the Basic and Expanded TBIs

Each holdout set is stratified into the groups B and NB, based on a TBI developed on the training set (using the POM (S1.1 in eAppendix 1) for WHO scores at day 14). For all six outcomes, the ORs comparing CCP vs. control are computed within the subgroups B and NB of the holdout set, and the ratio, OR(B)/OR(NB), are computed; a small ratio indicates a good performance.

| TBI | Q | Outcomes | | | | | | | | | | | | | | | | | |
|---|---|---|---|---|---|---|---|---|---|---|---|---|---|---|---|---|---|---|---|
| | | Day 14 WHO score | | | Day 28 WHO score | | | Day 14 WHO ≥ 7 | | | Day 28 WHO ≥ 7 | | | Day 14 mortality | | | Day 28 mortality | | |
| | | OR(B) | OR(NB) | B/NB | OR(B) | OR(NB) | B/NB | OR(B) | OR(NB) | B/NB | OR(B) | OR(NB) | B/NB | OR(B) | OR(NB) | B/NB | OR(B) | OR(NB) | B/NB |
| "Basic" | Q2 | 0.704 | 1.087 | 0.647 | 0.668 | 1.061 | 0.630 | 0.394 | 1.207 | 0.327 | 0.442 | 1.388 | 0.319 | 0.460 | 0.865 | 0.531 | 0.454 | 0.920 | 0.493 |
| | Q3 | 0.721 | 1.157 | 0.623 | 0.931 | 0.955 | 0.975 | 0.383 | 0.913 | 0.420 | 0.355 | 1.208 | 0.294 | 0.389 | 3.682 | 0.106 | 0.370 | 2.585 | 0.143 |
| | Q4 | 0.846 | 0.914 | 0.926 | 0.759 | 1.089 | 0.697 | 0.680 | 1.176 | 0.578 | 0.537 | 1.109 | 0.484 | 0.530 | 0.801 | 0.662 | 0.463 | 0.978 | 0.473 |
| | Q5 | 0.923 | 1.563 | 0.591 | 0.887 | 1.573 | 0.564 | 1.479 | 1.426 | 1.037 | 1.215 | 1.478 | 0.822 | 0.621 | 1.566 | 0.397 | 0.881 | 1.127 | 0.782 |
| "Expanded" | Q2 | 0.633 | 1.297 | 0.488 | 0.629 | 1.235 | 0.509 | 0.384 | 1.255 | 0.306 | 0.372 | 1.584 | 0.235 | 0.438 | 0.868 | 0.504 | 0.358 | 1.077 | 0.333 |
| | Q3 | 0.696 | 1.334 | 0.522 | 0.867 | 1.108 | 0.783 | 0.333 | 1.429 | 0.233 | 0.317 | 1.733 | 0.183 | 0.537 | 5.241 | 0.102 | 0.503 | 2.392 | 0.210 |
| | Q4 | 0.821 | 0.975 | 0.842 | 0.804 | 1.084 | 0.742 | 0.621 | 1.395 | 0.445 | 0.628 | 1.097 | 0.572 | 0.659 | 0.711 | 0.926 | 0.581 | 0.896 | 0.648 |
| | Q5 | 0.919 | 1.458 | 0.630 | 0.803 | 1.642 | 0.489 | 0.881 | 2.489 | 0.354 | 0.881 | 1.971 | 0.447 | 0.633 | 2.344 | 0.270 | 0.821 | 1.232 | 0.666 |

© 2022 Park H et al. *JAMA Network Open.*

# eTable 13. Value Results From a Leave-One-Enrollment-Quarter-Out Cross-validation

The holdout set is stratified into a group that is expected to benefit (B) and a group that is not expected to benefit (NB) from CCP treatment, based on a TBI developed on the training set (using the POM (S1.1 in eAppendix 1) for WHO scores at day 14). For the four binary outcomes, the expected values of outcomes under the TBI-based treatment decisions ("TBI") and those under two "treatment policies" – to treat everyone with CCP ("All CCP") and to treat no one with CCP ("No CCP")— are estimated based on the holdout set; small value are desired. The entries show the mean estimated expected values (in columns TBI, All CCP or No CCP) of outcomes across the 4 holdout enrollment quarters.

| Index | Outcomes | | | | | | | | | | | |
|---|---|---|---|---|---|---|---|---|---|---|---|---|
| | Day 14 WHO ≥ 7 | | | Day 28 WHO ≥ 7 | | | Day 14 mortality | | | Day 28 mortality | | |
| | TBI | All CCP | No CCP | TBI | All CCP | No CCP | TBI | All CCP | No CCP | TBI | All CCP | No CCP |
| J | 0.129 | 0.142 | 0.155 | 0.134 | 0.146 | 0.163 | 0.066 | 0.068 | 0.081 | 0.103 | 0.107 | 0.131 |
| S | 0.121 | 0.142 | 0.155 | 0.121 | 0.146 | 0.163 | 0.061 | 0.068 | 0.081 | 0.096 | 0.107 | 0.131 |
| V | 0.120 | 0.142 | 0.155 | 0.119 | 0.146 | 0.163 | 0.062 | 0.068 | 0.081 | 0.095 | 0.107 | 0.131 |
| **"Basic"** | **0.140** | **0.142** | **0.155** | **0.144** | **0.146** | **0.163** | **0.066** | **0.068** | **0.081** | **0.108** | **0.107** | **0.131** |
| **"Expanded"** | **0.114** | **0.142** | **0.155** | **0.119** | **0.146** | **0.163** | **0.06** | **0.068** | **0.081** | **0.098** | **0.107** | **0.131** |
| | **0.126** | **0.142** | **0.155** | **0.132** | **0.146** | **0.163** | **0.066** | **0.068** | **0.081** | **0.101** | **0.107** | **0.131** |

© 2022 Park H et al. *JAMA Network Open.*

## eAppendix 14. Comparisons With a Naive Treatment Decision Approach

In this subsection, for the Expanded TBI, we further examine the results presented in eTable 7. We display the distributions of the odds ratios for CCP vs. control, with respect to the six outcomes. Specifically, these distributions were obtained from the leave-one-third-out CV (1000 random splits of the data into 2/3^rds^ training and 1/3^rd^ testing), as described in eAppendix 3. The patient subgroup predicted to benefit (B; or "CCP-advised") and the patient subgroup predicted to not benefit (NB; or "CCP-not-advised") were identified by the TBI trained on each training set, and the subgroup-specific CCP efficacy odds ratios (OR(B) and OR(NB)) were evaluated based on the corresponding testing set; this process was repeated 1000 times.

This gave 1000 OR(B) and OR(NB), evaluated on 1000 test sets, corresponding to each of the six outcomes: a) the ordinal WHO score at day 14 (displayed in eFigure 5); b) the indicator of WHO ≥ 7 at day 14 (displayed in eFigure 7); c) the indicator of WHO=10 (death) at day 14 (displayed in eFigure 9); d) the ordinal WHO score at day 28 (displayed in eFigure 6); e) the indicator for WHO ≥ 7 at day 28 (displayed in eFigure 8); f) the indicatory of WHO=10 (death) at day 28 (displayed in eFigure 10).

For comparison, we also display the subgroup-specific (i.e., "CCP-advised" and "CCP-not-advised") CCP efficacy odds ratios for a "naïve" treatment decision rule (Naïve TDR), which is defined as follows: give a patient the CCP treatment if he/she satisfies one of the following conditions:

1. The number of days since COVID-19 symptoms onset is ≤ 6
2. The patient's baseline (11-point) WHO score = 4 (i.e., the baseline WHO score is not 5 or 6)
3. The patient has at least one of the following 2 medical conditions: 1) history of diabetes; 2) history of cardiovascular disease.

For each test set, the patients satisfying this criterion were considered to be in the "CCP-advised" (i.e., B) subgroup according to this "naïve" TDR, and the corresponding CCP efficacy OR was computed. Those who were not in the "CCP-advised" subgroup were considered to be in the "CCP-not-advised" (i.e., NB) subgroup, and the corresponding CCP efficacy OR was computed.

The distributions of the subgroup-specific ORs obtained from the 1000 testing sets, for each of the 6 outcomes, are displayed in eFigures 5-10. The top panel of each figure corresponds to the results of the TBI-based stratification and the bottom panel corresponds to those of the Naïve TDR. Within each panel, for the "CCP-advised" (displayed in blue) and the "CCP-not-advised" (displayed in red), the histograms and density estimates are displayed. In each panel, the location of the blue or red triangle on the horizontal-axis represents the median of each distribution. In addition, as a reference point, the median of the ORs evaluated on non-stratified testing samples ("Pooled") was denoted as a green triangle, which represents the overall CCP efficacy OR.

Overall, those who were identified as "CCP-advised" by the TBI clearly have stronger CCP efficacies (i.e., smaller ORs) compared to those identified by the Naïve TDR, with respect to all 6 outcomes. For example, for the important outcome of death at day 28 (see eFigure 10), in the bottom panel (corresponding to the naïve stratification approach), the locations of the blue and green triangles are almost the same, implying that the naïve TDR approach is not effective in identifying patients who are expected to benefit from the CCP treatment. Furthermore, in eFigures 5-10, the subgroup-specific (i.e., CCP-advised and CCP-not-advised) distributions have a much more extensive overlap with each other for the naïve TDR (bottom panels), compared to those from the TBI (top panels). This indicates that the naïve rule is not effective in identifying individual patients who benefit or not benefit from the CCP treatment compared to the TBI approach, with respect to all outcomes. In addition to this performance advantage, the TBI can quantify the magnitude of the expected relative benefit of taking CCP over control, as described in eFigure 28, which can help guide clinicians' treatment decisions.

© 2022 Park H et al. *JAMA Network Open.*

## eFigure 5. Distributions of Subgroup-Specific ORs Obtained From 1000 Randomly Split Testing Sets for the Ordinal Outcome WHO Score at Day 14

The subgroups (CCP-advised, displayed in blue; and CCP-not-advised, in red) are identified by the Expanded TBI (in the top panel) and the Naïve TDR (in the bottom panel).

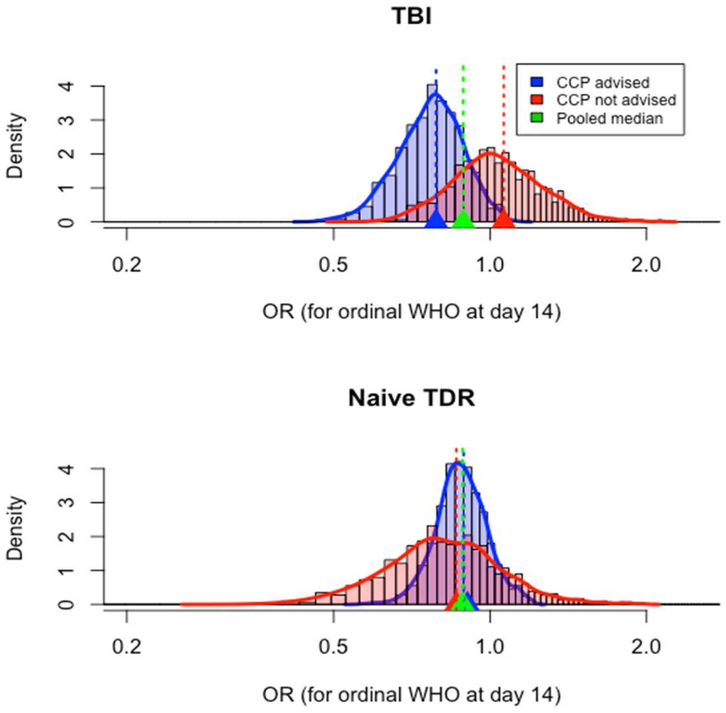


© 2022 Park H et al. *JAMA Network Open.*

## eFigure 6. Distributions of Subgroup-Specific ORs Obtained From 1000 Randomly Split Testing Sets for the Ordinal Outcome WHO Score at Day 28

Subgroups (CCP-advised, displayed in blue; and CCP-not-advised, in red) are identified by the Expanded TBI (in the top panel) and the Naïve TDR (in the bottom panel).

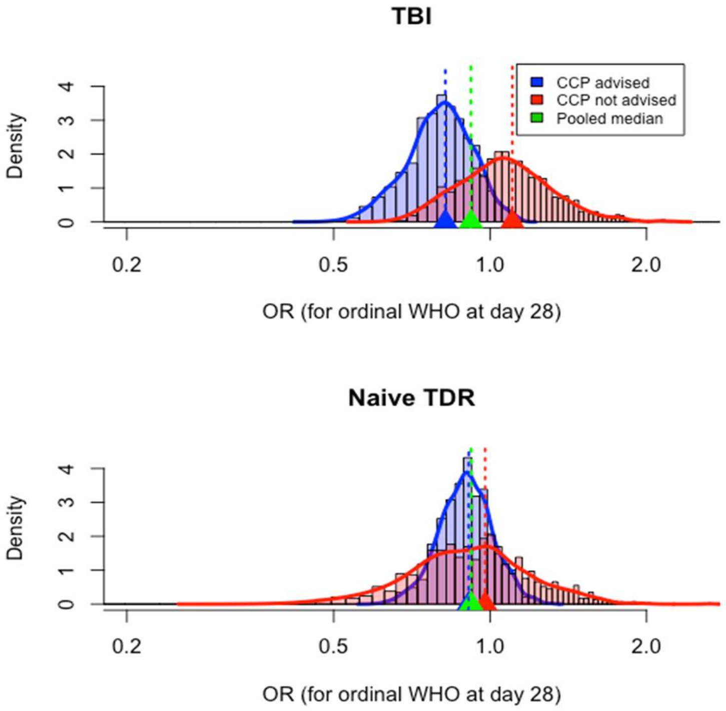


© 2022 Park H et al. *JAMA Network Open.*

## eFigure 7. Distributions of Subgroup-Specific ORs Obtained From 1000 Randomly Split Testing Sets for Binary Outcome of WHO Score of at Least 7 at Day 14

Subgroups (CCP-advised, displayed in blue; and CCP-not-advised, in red) are identified by the Expanded TBI (in the top panel) and the Naïve TDR (in the bottom panel).

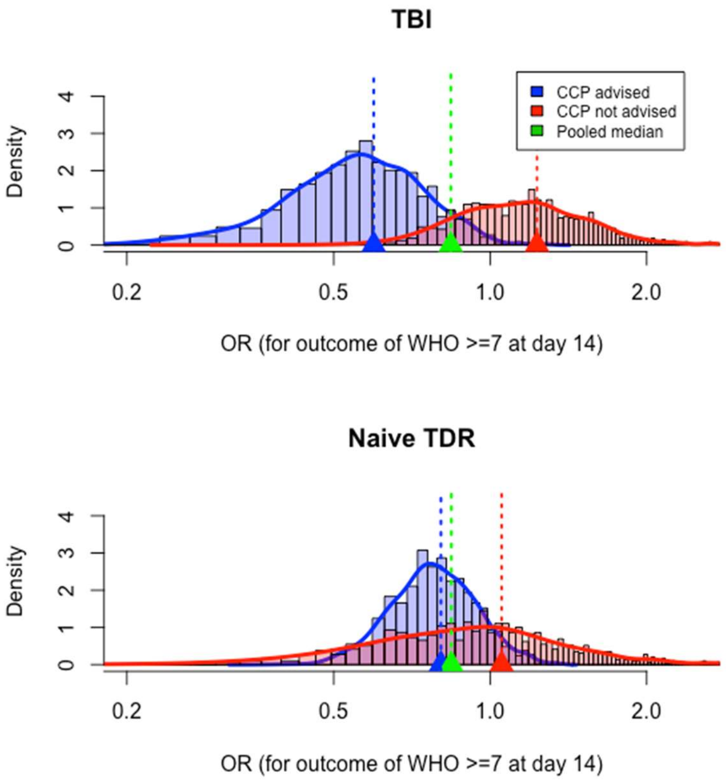


© 2022 Park H et al. *JAMA Network Open.*

## eFigure 8. Distributions of Subgroup-Specific ORs Obtained From 1000 Randomly Split Testing Sets for Binary Outcome of WHO Score of at Least 7 at Day 28

Subgroups (CCP-advised, displayed in blue; and CCP-not-advised, in red) are identified by the Expanded TBI (in the top panel) and the Naïve TDR (in the bottom panel).

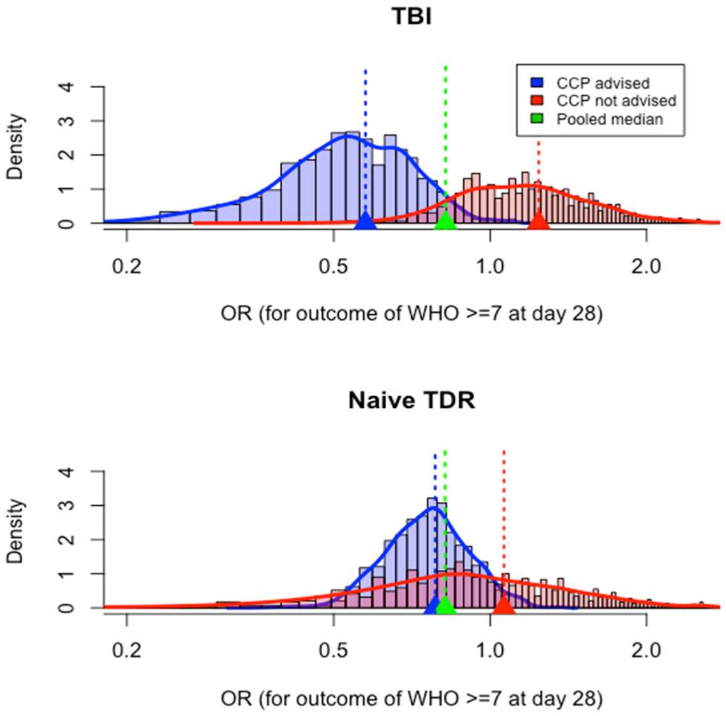


© 2022 Park H et al. *JAMA Network Open.*

## eFigure 9. Distributions of Subgroup-Specific ORs Obtained From 1000 Randomly Split Testing Sets for Death at Day 14

Subgroups (CCP-advised, displayed in blue; CCP-not-advised, displayed in red) are identified by the Expanded TBI (in the top panel) and by the Naïve TDR (in the bottom panel).

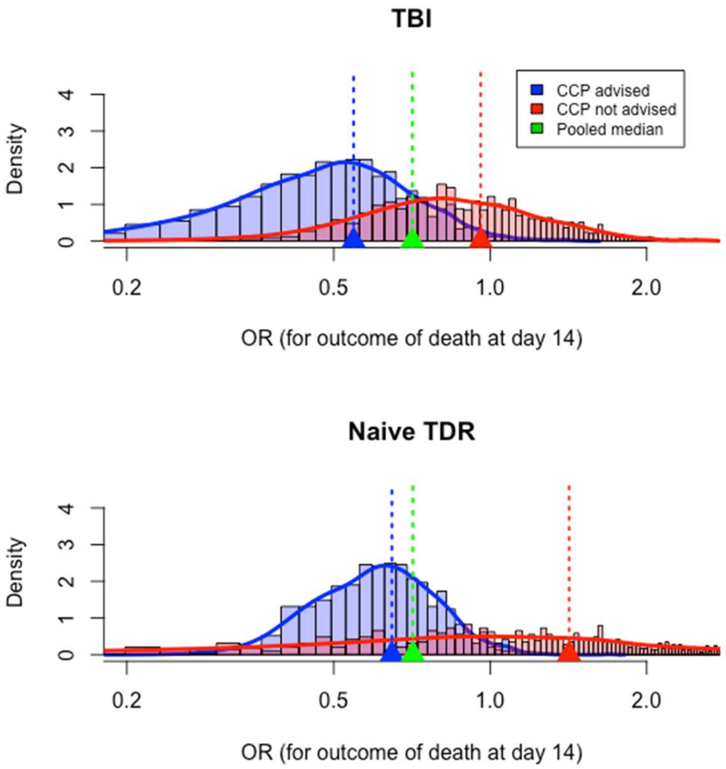


© 2022 Park H et al. *JAMA Network Open.*

## eFigure 10. Distributions of Subgroup-Specific ORs Obtained From 1000 Randomly Split Testing Sets for Death at Day 28

Subgroups (CCP-advised, displayed in blue; CCP-not-advised, displayed in red) are identified by the Expanded TBI (in the top panel) and by the Naïve TDR (in the bottom panel).

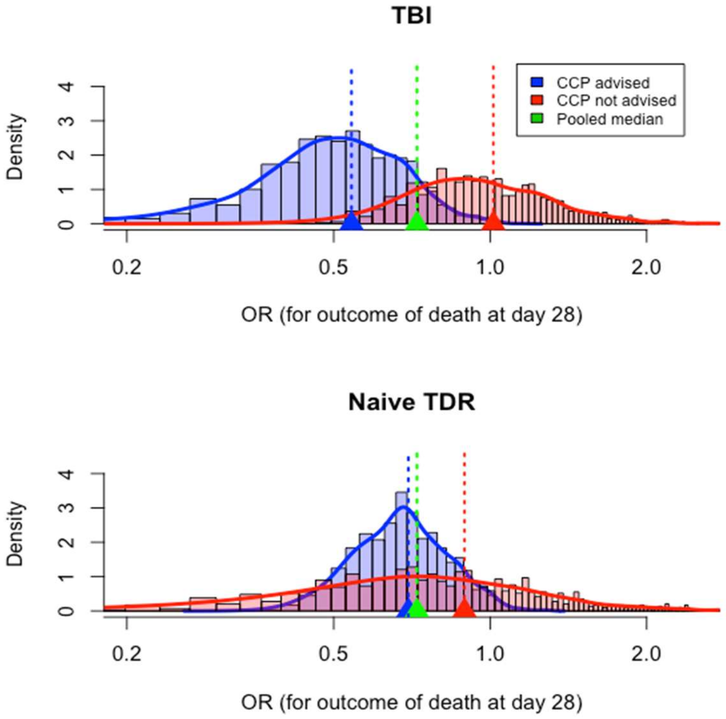


© 2022 Park H et al. *JAMA Network Open.*

## eAppendix 15. Specification of the Basic and Expanded TBIs

Overall, in the 1000 split-sample simulation CV (see eTable 7 and 8), the index "Expanded" outperformed the indices S, V and J, with respect to most of the outcomes. The index "Expanded" also consistently outperformed or exhibited a comparable performance as these indices, both in the leave-one-enrollment-quarter-out CV and leave-one-RCT-out CV (see eTable 9-13). In addition to its superior CV performance, the index "Expanded" is simpler and more interpretable than the indices S and V, while generally outperforming the index J. Therefore, we nominated the index "Expanded" as the expanded TBI for the CCP treatment. On the other hand, among those indices not requiring the patient's blood type information, the index "Basic" clearly exhibited the best performance in all the CVs considered. Therefore, we nominated the index "Basic" as the basic TBI, which requires less information than the expanded TBI. When the patient's blood type information is not available, the basic TBI can be used instead of the expanded TBI.

The variables and the coefficients ($\boldsymbol{\beta}$) of linear combination (along with 95% bootstrap confidence intervals; the confidence intervals that do not include 0 are marked with *) under model (S1.1 in eAppendix 1) for the final selected TBIs, i.e., the "Basic" and the "Expanded" TBIs, are given in eTable 14 below. Note that the significance (*) in the table is not used for keeping or eliminating terms in the TBI; significance is indicated only as a guidance. The retention of terms in the indices is selected via the CV process outlined above.

© 2022 Park H et al. *JAMA Network Open.*

# eTable 14. Coefficients of the Linear Combination for Baseline Patient Characteristics in the Basic and Expanded TBIs

The TBIs are continuous measures between 0 and 1. In addition to specifying the cut-point (0.25 and 0.27 for the Basic and Expanded TBIs, respectively; see eAppendix 2 for how the cut-point was determined) for the group predicted to benefit (B) and the group predicted to not benefit (NB), the last row of the Table gives the cut-points (see eAppendix 6 for how these cut-points were determined) on the TBIs that define the three benefit-levels which conceptualize the continuous TBI for clinical utility: B1 with expected large benefit; B2 with expected modest benefit; and B3 with expected potential harm.

| Baseline characteristics | Coefficients (95% bootstrap CIs) | |
|---|---|---|
| | **Basic TBI** | **Expanded TBI** |
| Intercept | 0.41 (0.09, 0.73)* | 0.36 (0.08, 0.65)* |
| Indicator for baseline WHO=5 | -0.26 (-0.52, -0.01)* | -0.24 (-0.45, -0.04)* |
| Indicator for baseline WHO=6 | -0.19 (-0.55, 0.16) | -0.17 (-0.48, 0.13) |
| Indicator for baseline WHO=5 & age ≥67 | -0.05 (-0.30, 0.21) | -0.04 (-0.26, 0.17) |
| Indicator for baseline WHO=6 & age ≥67 | -0.22 (-0.72, 0.29) | -0.19 (-0.60, 0.22) |
| Indicator for blood type A or AB | -- | 0.14 (-0.07, 0.36) |
| Indicator for presence of CVD | 0.23 (0.01, 0.45)* | 0.19 (0.00, 0.38)* |
| Indicator for comorbid DM & PD | 0.36 (-0.06, 0.78) | 0.30 (-0.12, 0.72) |
| Defining expected benefit (B) and no expected benefit (NB) groups | | |
| Cut-points for 2 benefit-level groups | 0.25 | 0.27 |
| Defining high benefit (B1), modest benefit (B2) and no expected benefit (B3) groups | | |
| Cut-points for 3 benefit-level groups | 0.2 and 0.4 | 0.2 and 0.37 |

CVD=Cardiovascular Disease
DM=Diabetes Mellitus
PD=Pulmonary Disease

© 2022 Park H et al. *JAMA Network Open.*

## eAppendix 16. Cross-Stratification Table Comparing the Basic and Expanded TBIs

We follow the risk stratification table approach[13] to cross-tabulate treatment benefit predictions with (i.e., using the Expanded TBI) and without blood type information (i.e., using the Basic TBI). eTable 15 analyzes the extent to which the benefits calculated from the models reflect the actual benefit in the population (model fidelity), and compares the two TBIs (i.e., the indices with and without blood type information), in terms of agreement between the treatment benefit stratifications.

The benefit index has a capacity to stratify the population into different benefit levels. We use stratification specified in the last row of eTable 14, i.e., the three benefit groups (B1, B2, B3) stratification. The stratification cut-points were determined as described in eAppendix 6. As indicated in eAppendix 18, these TBIs' cut-points (i.e., 0.2 and 0.4 for the Basic TBI, and 0.2 and 0.37 for the Expanded TBI) roughly corresponded to the model-based CCP efficacy ORs of 1.10 and 0.80, for the outcome of the ordinal WHO score at day 14, under the TBI model (S1.3).

eTable 15 analyzes the models with and without blood type information (the Expanded and Basic TBIs, respectively), in terms of model fidelity. Model fidelity means that the model-predicted treatment efficacy OR for a person with specified predictor values is close to the actual treatment efficacy OR of persons in the population with those same predictor values. Since there are often very few patients with the same predictor values, for evaluation, the patients are aggregated into pre-specified categories as in eTable 15. The fidelity of the benefit prediction models can be assessed by comparing the ORs in the margins of eTable 15 with the corresponding row and column labels.

For example, for the **Expanded TBI**, the observed benefits within each benefit category (B1, B2, B3) are in the bottom "Total" row, and they generally agree with the column labels. Similarly, for the **Basic TBI**, the observed benefits within each benefit category are in the far-right "Total" column, and they generally agree with the row labels. Thus, both models seem to be well calibrated (under the proportional odds assumption).

As indicated in eTable 14, the blood type information enters the CCP benefit calculation, through the indicator of the blood types A or AB, with the associated coefficient 0.14 (> 0). In eTable 15, the ORs within each row (see, e.g., the row associated with B2, specified by the Basic TBI) has an increasing trend, as we move from left to right (i.e., moving from B1 to B2, and to B3, determined from the Expanded TBI). This indicates that, by incorporating blood type information through the Expanded TBI, the patients sorted by the Basic TBI are further sorted according to their benefit level in a more refined manner. That is, the Expanded TBI provides a more refined gradation of the treatment benefit.

In eTable 15, we note that there was no patient classified to the benefit group B1 (or B3) by one TBI while being classified to the benefit group B3 (or B1) by the other TBI. That is, there was no large disagreement between the two stratifications. The two TBI's Pearson correlation is 0.90, when they are treated as continuous variables.

[13] Janes, H., Pepe, M., Gu, W. Assessing the value of risk predictions by using risk stratification tables. *Annals of Internal Medicine.* 2008;149(10):751-760.

© 2022 Park H et al. *JAMA Network Open.*

## eTable 15. Benefit Stratification Table

Cross-tabulated by the three benefit groups (B1, B2, B3) of the Basic and Expanded TBIs. The ORs in the inner cells of the table are computed under POM without adjusting for any covariates, using the outcome of ordinal WHO at day 14.

| **Basic TBI** ($TBI_B$, without blood type information) | **Expanded TBI** ($TBI_E$, with blood type information) | | | Total |
|---|---|---|---|---|
| | **B1**: ($TBI_E > 0.37$) OR < 0.8 | **B2**: ($0.2 \leq TBI_E < 0.37$) $0.8 \leq$ OR <1.1 | **B3**: ($TBI_E < 0.2$) OR > 1.1 | |
| **B1**: ($TBI_B > 0.4$) OR < 0.8 | n= 364 OR= 0.63 | n= 233 OR= 0.85 | n= 0 OR= na | n= 597 OR= 0.74 |
| **B2**: ($0.2 \leq TBI_B < 0.4$) $0.8 \leq$ OR < 1.1 | n= 265 OR= 0.72 | n= 454 OR= 0.75 | n= 160 OR= 1.49 | n= 879 OR= 0.86 |
| **B3**: ($TBI_B < 0.2$) OR > 1.1 | n= 0 OR= na | n= 266 OR= 0.81 | n= 545 OR= 1.21 | n= 811 OR= 1.07 |
| Total | n= 629 OR= 0.67 | n= 953 OR= 0.81 | n= 705 OR= 1.29 | n= 2287 OR= 0.89 |

© 2022 Park H et al. *JAMA Network Open.*

## eAppendix 17. Proportional Odds Assumption Test

POM (S1.1 in eAppendix 1) is a parsimonious special case of the cumulative logit model. The model assumes that the effect of covariates-by-treatment interactions is identical for all (11-1=)10 cumulative logits. If this model fits well, it requires only a single component ($g(\boldsymbol{\beta}^T \boldsymbol{X}, A)$) to describe the heterogeneous treatment effect, allowing a simple treatment classification rule based on the odds ratio (S1.3) common to all cumulative odds. On the other hand, a more general cumulative logit model permits the heterogeneous treatment effect to vary across the 10 cumulative logits.

In both the Basic and Expanded TBIs, we first note that the functions $g(.)$ associated with heterogeneous treatment effect (HTE) in POM (S1.1 in eAppendix 1) were estimated to be essentially linear. Thus, we restrict our attention to a linear cumulative logit model for testing the proportional odds assumption associated with HTE, for the sake of simplicity. Given the TBI $U = \boldsymbol{\beta}^T \boldsymbol{X}$ (either Basic or Expanded TBI), we consider a model

$$logit\big(P(Y \geq y)\big) = \tau_y + \mu(\boldsymbol{Z}) + A\alpha_y + AU\gamma_y, \qquad y = 1,2,\dots,10, \qquad (S3.1)$$

for cumulative logits, $logit\big(P(Y \geq y)\big),\ y = 1,2,\dots,10$, where HTE is allowed to vary across the cumulative logits. In particular, HTE is described by $A\alpha_y + AU\gamma_y$. In (S3.1), we use a centered treatment variable ($A = -0.5$ for control and $A = 0.5$ for CCP) to make the HTE term orthogonal to the other term $\tau_y + \mu(\boldsymbol{Z})$, making it robust against the misspecification of the TBI and covariates $\boldsymbol{Z}$'s main effect[14] (note, $\mu(\boldsymbol{Z})$ is restricted to be linear in $\boldsymbol{Z}$). In eTable 16 below, we display the estimated coefficients $\alpha_y$ and $\gamma_y$, where the coefficients are estimated from separate logistic regression for each $y = 1,2,\dots,10$.

In eTable 16, except for those associated with the cumulative logits corresponding to lower WHO-scales (y=1, 2, 3), the estimates of the coefficients $\alpha_y$ and $\gamma_y$ appear to vary relatively little across the cumulative logits of y=4, 5, …, 10; for the Basic TBI, $\alpha_y$ range from 0.53 to 0.81, and $\gamma_y$ range from -2.05 to -3.20; for the Expanded TBI, $\alpha_y$ range from 0.73 to 1.05, and $\gamma_y$ range from -2.51 to -3.55, suggesting that the assumption of proportional odds is practically reasonable.
In the following, we report results from a Brant test[15] for ordinal logit models (S3.1), investigating whether the parallel regression assumption significantly deviates from the observed data. The null hypothesis ($H_0$) of the test is given by: $\alpha_y = \alpha$ and $\gamma_y = \gamma$ for all $y = 1,2,\dots,10$, for some $\alpha \in \mathbb{R}$ and $\gamma \in \mathbb{R}$.

In eTable 17, that the resulting p-values are all greater than 0.1 indicates that the observed deviation from the proportional odds assumption associated with HTE is not statistically significant at a significance level $\alpha$=0.1. The analysis results in eTable 16 and 17 suggest that using POM is likely to be more robust and effective compared to a more general cumulative logit model for derivation of the TBIs.

[14] Tian L, Alizadeh A, Gentles A, Tibshirani R. A simple method for estimating interactions between a treatment and a large number of covariates. *Journal of the American Statistical Association*. 2014;109:508, 1517-1532.

[15] Brant, R. Assessing proportionality in the proportional odds model for ordinal logistic regression. *Biometrics.* 1990;46:1171-1178.

© 2022 Park H et al. *JAMA Network Open.*

## eTable 16. Estimates of Coefficients Associated With HTE

Coefficients (and 95% CIs) are estimated from separate logistic regression for each $y = 1,2,\ldots,10$.

| $logit(P(Y \geq y))$ | Basic TBI | | Expanded TBI | |
|---|---|---|---|---|
| | $\alpha_y$ | $\gamma_y$ | $\alpha_y$ | $\gamma_y$ |
| y=1 | 0.19 (-0.38, 0.76) | -1.17 (-2.81, 0.47) | 0.10 (-0.47, 0.68) | -0.88 (-2.55, 0.78) |
| 2 | 0.35 (-0.07, 0.78) | -1.47 (-2.70, -0.23) | 0.37 (-0.06, 0.80) | -1.50 (-2.76, -0.25) |
| 3 | 0.50 (0.12, 0.88) | -1.77 (-2.90, -0.64) | 0.59 (0.20, 0.98) | -2.04 (-3.19, -0.89) |
| 4 | 0.58 (0.17, 0.99) | -2.05 (-3.27, -0.83) | 0.73 (0.31, 1.16) | -2.51 (-3.76, -1.27) |
| 5 | 0.66 (0.23, 1.09) | -2.28 (-3.56, -1.01) | 0.78 (0.33, 1.22) | -2.61 (-3.92, -1.31) |
| 6 | 0.81 (0.35, 1.26) | -2.69 (-4.06, -1.32) | 1.05 (0.56, 1.53) | -3.42 (-4.84, -2.00) |
| 7 | 0.67 (0.18, 1.16) | -2.80 (-4.26, -1.33) | 0.92 (0.40, 1.44) | -3.55 (-5.07, -2.02) |
| 8 | 0.60 (0.08, 1.12) | -2.82 (-4.37, -1.26) | 0.78 (0.23, 1.33) | -3.34 (-4.96, -1.72) |
| 9 | 0.53 (-0.04, 1.09) | -3.18 (-4.87, -1.49) | 0.61 (0.01, 1.21) | -3.35 (-5.12, -1.59) |
| 10 | 0.60 (-0.03, 1.23) | -3.20 (-5.09, -1.32) | 0.67 (0.00, 1.34) | -3.33 (-5.31, -1.35) |

© 2022 Park H et al. *JAMA Network Open.*

# eTable 17. Brant Test Results

For testing $H_0$: Parallel regression assumption holds for (S3.1).

| | **Basic TBI** | | | **Expanded TBI** | | |
|---|---|---|---|---|---|---|
| | $\chi^2$ | d.f. | p-value | $\chi^2$ | d.f. | p-value |
| Omnibus | 20.01 | 18 | 0.33 | 23.54 | 18 | 0.17 |
| $\alpha_y$ | 5.74 | 9 | 0.77 | 11.1 | 9 | 0.27 |
| $\gamma_y$ | 4.92 | 9 | 0.84 | 8.51 | 9 | 0.48 |

© 2022 Park H et al. *JAMA Network Open.*

## eAppendix 18. Three Levels of Benefit for the Basic and Expanded TBIs

An advantage of the TBI approach to developing treatment classification rules is the continuous nature of the index, which allows capitalizing on the gradation of the expected benefit. Our investigations suggested that there was a sub-group that was expected to have a substantial benefit among patients expected to benefit from CCP and a subgroup with potential harm from CCP. With an ability to capture the different degrees of the CCP treatment benefit, the TBI allows providing more refined guidelines regarding the expected benefit. For instance, the expected benefit from CCP can be categorized into the three levels: large benefit (B1), modest benefit (B2) and potential harm (B3) from CCP treatment.

In eFigure 11 and eFigure 12 below, the model-based cumulative OR functions $f(\cdot)$ in (S1.3 in eAppendix 17) are displayed as functions of the Basic (in eFigure 11) and Expanded (in eFigure 12) TBIs (i.e., $\boldsymbol{\beta}^T\boldsymbol{X}$), respectively. The two benefit levels (i.e., B and NB), determined by the model-based CCP efficacy OR of 1, are displayed in the left panel (the corresponding cut-point was 0.25 for the Basic TBI, and 0.27 for the Expanded TBI), and the three benefit levels (B1, B2 and B3) are displayed in the right panel. The cut-points (a, b) defining the 3 benefit groups (TBI $< a$; $a \leq$ TBI $< b$; and TBI $\geq b$) were chosen to optimize the 3-category model fit, as described in eAppendix 6. These cut-points are (a, b) = (0.2, 0.4) for the Basic TBI, and (a, b) = (0.2, 0.37) for the Expanded TBI, as specified in eTable 14. For both TBIs, these cut-points roughly corresponded to the model-based CCP efficacy cumulative ORs of 1.10 and 0.80, respectively, for the outcome of the ordinal WHO score at day 14, under the TBI model (S1.3).

© 2022 Park H et al. *JAMA Network Open.*

## eFigure 11. Basic TBI Model-Based Cumulative Odds Ratio for the Ordinal WHO Score at Day 14, as a Function of the Basic TBI

Basic TBI specified in eTable 14 Two-groups stratification (B and NB) is displayed in the left (panel a) and three-groups stratification (B1, B2 and B3) is displayed in the right (panel b). The within group CCP efficacy odds ratios (for the outcome of the ordinal WHO at day 14, unadjusted for any covariates) are provided in the legends.

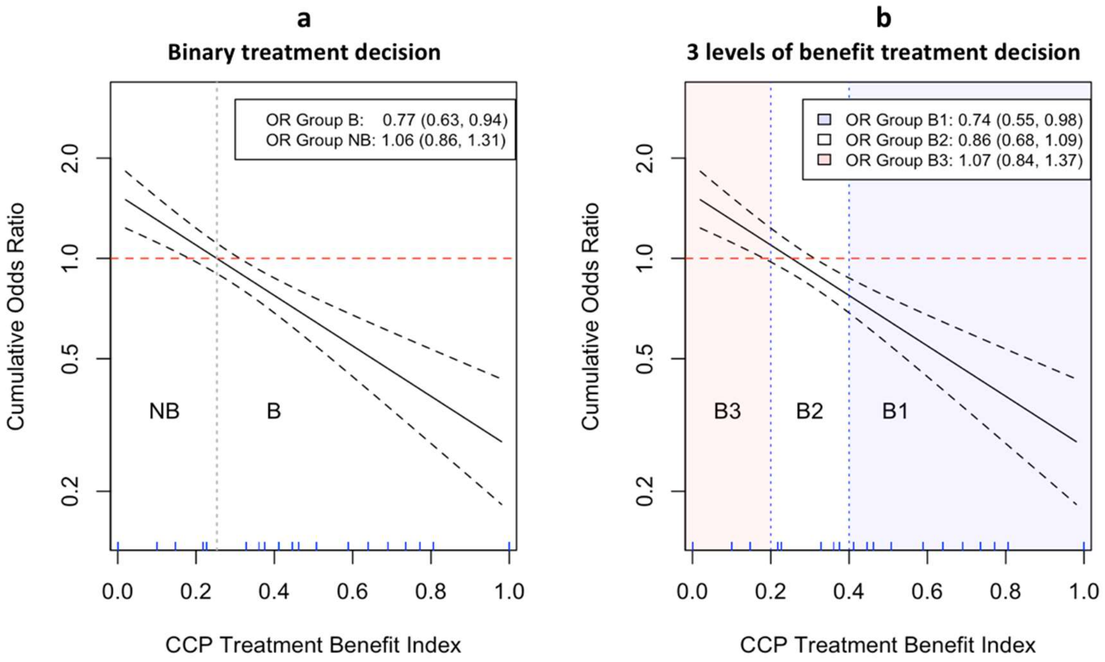


© 2022 Park H et al. *JAMA Network Open.*

## eFigure 12. Expanded TBI Model-Based Cumulative Odds Ratio for the Ordinal WHO Score at Day 14, as a Function of the Expanded TBI

*Expanded TBI* specified in eTable 14. The two-groups stratification (B and NB) is given in the left (panel a) and the three-groups stratification (B1, B2 and B3) is given in the right (panel b). The within group CCP efficacy odds ratios (for the outcome of the ordinal WHO at day 14, unadjusted for any covariates) are provided in the legends.

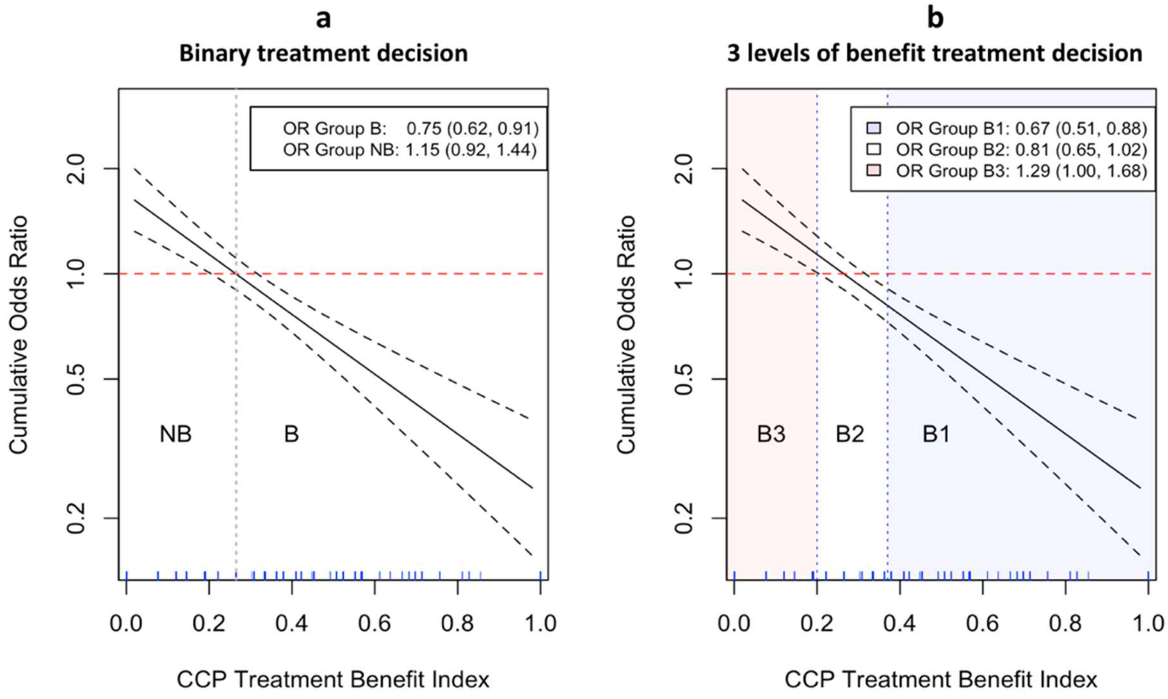


The subgroup-specific CCP efficacy cumulative ORs indicated in the legends on eFigure 11 and eFigure 12 above were estimated based on frequentist analyses. (Bayesian estimates are provided in eFigure 23 for the Basic TBI, and in eFigure 14 for the Expanded TBI.) In eFigure 20, we display unadjusted CCP efficacy ORs as functions of the Basic TBI for all six outcomes. In Figure 1 of the main manuscript, we display unadjusted CCP efficacy ORs as functions of the Expanded TBI for all six outcomes.

eTable 18 and eTable 19 report the results from CV when the expected benefit from CCP is categorized into the three benefit levels (i.e., B1, B2 and B3).

In eTable 18 and eTable 19, although the relationships of the odds ratios OR(B1) < OR(B2) < OR(B3) and of the expected incidences E(B1) < E(B2) < E(B3) for the Leave-one-RCT-out CV and the Leave-one-enrollment-quarter-out CV were not as clear (especially for some benefit levels in the Basic TBI) in comparison to the more extensive split-sample-simulation CV (1000 random splits) given in the first section of each of the tables, there is strong support for the generalizability of the TBIs in distinguishing groups with different benefit levels.

© 2022 Park H et al. *JAMA Network Open.*

# eTable 18. Odds Ratios Associated with All 6 Outcomes, Under 3 Levels of Categorization From the Basic and Expanded TBIs

The TBIs are developed using the POM (S1.1 in eAppendix 1) for WHO scores at day 14. Based on a TBI developed on the training set, the holdout set is stratified into 3 groups: large benefit (B1), modest benefit (B2), and potential harm (B3) from CCP. The ORs for all six outcomes are computed within each subgroup (B1, B2 and B3) of the holdout set. The entries show the median (for the 1000 split-sample-simulation) or the mean (for the leave-one-RCT-out and the leave-one-enrollment-quarter-out CVs) of the ORs computed across the holdout sets.

| Index | Outcomes | | | | | | | | | | | | | | | | | |
|---|---|---|---|---|---|---|---|---|---|---|---|---|---|---|---|---|---|---|
| | Day 14 WHO ordinal | | | Day 28 WHO ordinal | | | Day 14 WHO ≥ 7 | | | Day 28 WHO ≥ 7 | | | Day 14 mortality | | | Day 28 mortality | | |
| | OR(B1) | OR(B2) | OR(B3) | OR(B1) | OR(B2) | OR(B3) | OR(B1) | OR(B2) | OR(B3) | OR(B1) | OR(B2) | OR(B3) | OR(B1) | OR(B2) | OR(B3) | OR(B1) | OR(B2) | OR(B3) |
| **1) Split-sample-simulation CV (1000 random splits)** | | | | | | | | | | | | | | | | | | |
| "Basic" | 0.760 | 0.895 | 1.011 | 0.776 | 0.927 | 1.053 | 0.563 | 0.858 | 1.089 | 0.507 | 0.857 | 1.124 | 0.428 | 0.780 | 0.891 | 0.450 | 0.780 | 0.968 |
| "Expanded" | 0.764 | 0.875 | 1.098 | 0.798 | 0.913 | 1.126 | 0.541 | 0.860 | 1.289 | 0.498 | 0.874 | 1.308 | 0.468 | 0.788 | 0.909 | 0.476 | 0.771 | 1.039 |
| **2) Leave-one-RCT-out CV** | | | | | | | | | | | | | | | | | | |
| "Basic" | 0.700 | 0.802 | 1.211 | 0.594 | 0.770 | 1.192 | 0.662 | 0.703 | 1.758 | 0.564 | 0.884 | 1.903 | 0.421 | 0.772 | 1.341 | 0.818 | 0.719 | 1.954 |
| "Expanded" | 0.827 | 0.749 | 1.494 | 0.736 | 0.710 | 1.598 | 0.659 | 0.698 | 1.973 | 0.624 | 0.891 | 1.549 | 0.586 | 0.655 | 0.845 | 1.011 | 1.135 | 1.153 |
| **3) Leave-one-enrollment-quarter-out CV** | | | | | | | | | | | | | | | | | | |
| "Basic" | 0.654 | 0.907 | 1.177 | 0.778 | 0.891 | 1.134 | 0.531 | 1.047 | 1.295 | 0.513 | 0.918 | 1.422 | 0.351 | 1.454 | 1.164 | 0.401 | 0.932 | 1.279 |
| "Expanded" | 0.657 | 0.865 | 1.408 | 0.714 | 0.865 | 1.430 | 0.486 | 0.855 | 1.745 | 0.422 | 0.798 | 1.954 | 0.365 | 1.022 | 2.094 | 0.398 | 0.768 | 2.194 |

© 2022 Park H et al. *JAMA Network Open.*

## eTable 19. Results Regarding the Expected Incidence (Value) of the 4 Clinically Undesirable Binary Outcomes When CCP is Administered to the 3 Patient Subgroups Identified by the Basic and Expanded TBIs

These within-group expected incidences are estimated via CV, based on: **1**) 1000 random splits into training data set (2/3[rds]) and testing data set (1/3[rd]) (i.e., split-sample-simulation) CV; **2**) leave-one-RCT-out CV; and **3**) leave-one-enrollment-quarter-out CV. Based on a TBI developed on the training set, the testing set is stratified into B1, B2 and B3, and the corresponding incidences were estimated. The entries show the median (for the 1000 split-sample-simulation CV) or the mean (for the leave-one-RCT-out and the leave-one-enrollment-quarter-out CVs) of the estimated incidences, across the holdout samples.

| Index | Outcomes | | | | | | | | | | | |
|---|---|---|---|---|---|---|---|---|---|---|---|---|
| | Day 14 WHO ≥ 7 | | | Day 28 WHO ≥ 7 | | | Day 14 mortality | | | Day 28 mortality | | |
| | B1 | B2 | B3 | B1 | B2 | B3 | B1 | B2 | B3 | B1 | B2 | B3 |
| 1) Split-sample-simulation CV (1000 random splits) | | | | | | | | | | | | |
| “Basic” | 0.099 | 0.137 | 0.171 | 0.101 | 0.146 | 0.172 | 0.045 | 0.065 | 0.074 | 0.075 | 0.106 | 0.119 |
| “Expanded” | 0.100 | 0.131 | 0.191 | 0.101 | 0.138 | 0.194 | 0.049 | 0.062 | 0.081 | 0.078 | 0.099 | 0.137 |
| 2) Leave-one-RCT-out CV | | | | | | | | | | | | |
| “Basic” | 0.079 | 0.144 | 0.191 | 0.084 | 0.162 | 0.185 | 0.028 | 0.069 | 0.112 | 0.068 | 0.113 | 0.152 |
| “Expanded” | 0.123 | 0.118 | 0.215 | 0.124 | 0.130 | 0.212 | 0.054 | 0.078 | 0.132 | 0.089 | 0.101 | 0.180 |
| 3) Leave-one-enrollment-quarter-out CV | | | | | | | | | | | | |
| “Basic” | 0.120 | 0.180 | 0.150 | 0.120 | 0.190 | 0.150 | 0.040 | 0.100 | 0.070 | 0.080 | 0.150 | 0.100 |
| “Expanded” | 0.100 | 0.110 | 0.220 | 0.100 | 0.120 | 0.230 | 0.040 | 0.060 | 0.100 | 0.080 | 0.090 | 0.170 |

© 2022 Park H et al. *JAMA Network Open.*

# eAppendix 19. Baseline Patient Characteristics by Benefit Levels

eTable 20 describes the baseline characteristics of patients in the three benefit-level groups (B1, B2 and B3) determined from the Expanded TBI.

In eTable 20, in the rows associated with the enrollment quarters, we only reported the distribution of the patient population across the enrollment quarters within each benefit group (i.e., each column sums to 1). On the other hand, in eFigure 13 below, we display the distribution of the patient population across the different benefit groups (B1, B2 and B3) by each quarter, to investigate whether the patient population has shifted over time and its possible relationship with the observed shifted CCP efficacy presented in eFigure 2.

In eFigure 13 below, a notable observation is that the proportion of patients in B3 increases over time, with B3 (the group predicted to be potentially harmed by CCP) having the largest prevalence of patients enrolled between January and March 2021, that had the lowest CCP efficacy. This partly explains the observed CCP efficacy that decreased over time in the COMPILE study.

Additionally, we provide below the binary subgroup characteristics where the subgroups were identified based on the CCP efficacy OR of 1 (ORε1 or OR<1) (see eAppendix 2), under the TBI model (S1.3 in eAppendix 1), i.e., the subgroup of the patients who were predicted to benefit (B) and the subgroup of the patients who were not predicted to benefit (NB) from the CCP treatment, predicted by the Expanded TBI.

© 2022 Park H et al. *JAMA Network Open.*

## eTable 20. Baseline Characteristics of COMPILE Patients in the 3 Benefit-Level Groups Defined From the Expanded TBI

For categorical variables each column sums to 100%).

| Patient Characteristics | Overall mean (SD) or proportion (n=2287) | Subgroup mean (SD) or proportion in % | | | Test for difference in means (or proportions) | |
|---|---|---|---|---|---|---|
| | | Group B1 (n=629) | Group B2 (n=953) | Group B3 (n=705) | F-statistic | P-value ∗(signf.) |
| Age in years | 60.31(15.2) | 63.77(13.8) | 59.35(15.5) | 58.51(15.5) | 23.69 | <10-2 * |
| Sex (proportion) | | | | | | |
| Female | 36 % | 34 % | 35 % | 38 % | 1.78 | 0.17 |
| Male | 64 % | 66 % | 65 % | 62 % | 1.78 | 0.17 |
| Baseline WHO (proportion) | | | | | | |
| 4 | 20 % | 34 % | 24 % | 0 % | 766.43 | <10-2 * |
| 5 | 63 % | 51 % | 66 % | 69 % | 262.31 | <10-2 * |
| 6 | 18 % | 15 % | 10 % | 31 % | 65.57 | <10-2 * |
| Blood type (proportion) | | | | | | |
| O | 46 % | 19 % | 46 % | 72 % | 224.64 | <10-2 * |
| A | 33 % | 68 % | 34 % | 3 % | 437.94 | <10-2 * |
| B | 16 % | 6 % | 16 % | 25 % | 50.16 | <10-2 * |
| AB | 4 % | 8 % | 4 % | 0 % | 26.40 | <10-2 * |
| Diabetes (proportion) | | | | | | |
| Yes | 34 % | 49 % | 32 % | 22 % | 60.25 | <10-2 * |
| Pulmonary disease (proportion) | | | | | | |
| Yes | 12 % | 23 % | 7 % | 8 % | 52.97 | <10-2 * |
| Cardiovascular (proportion) | | | | | | |
| Yes | 42 % | 79 % | 43 % | 8 % | 489.83 | <10-2 * |
| Enrollment quarter (proportion) | | | | | | |
| April – June 2020 | 27 % | 28 % | 31 % | 22 % | 8.50 | <10-2 * |
| July – September 2020 | 20 % | 20 % | 19 % | 20 % | 0.16 | 0.85 |
| October – December 2020 | 38 % | 38 % | 37 % | 39 % | 0.34 | 0.71 |
| January – March 2021 | 15 % | 14 % | 13 % | 19 % | 6.92 | <10-2 * |
| Days since symptoms (proportion) | | | | | | |
| 0 to 3 | 12 % | 14 % | 11 % | 13 % | 1.70 | 0.18 |
| 4 to 6 | 36 % | 36 % | 35 % | 36 % | 0.18 | 0.84 |
| 7 to 10 | 36 % | 36 % | 36 % | 35 % | 0.18 | 0.84 |
| 11 to 14 | 11 % | 10 % | 12 % | 10 % | 0.85 | 0.43 |
| More than 14 | 6 % | 4 % | 6 % | 7 % | 2.61 | 0.07 |

© 2022 Park H et al. *JAMA Network Open.*

## eFigure 13. Proportions of the 3 Benefit-Level Groups Identified From the Expanded TBI, by Quarter

A notable observation is that the proportion of patients in B3 increased over time.

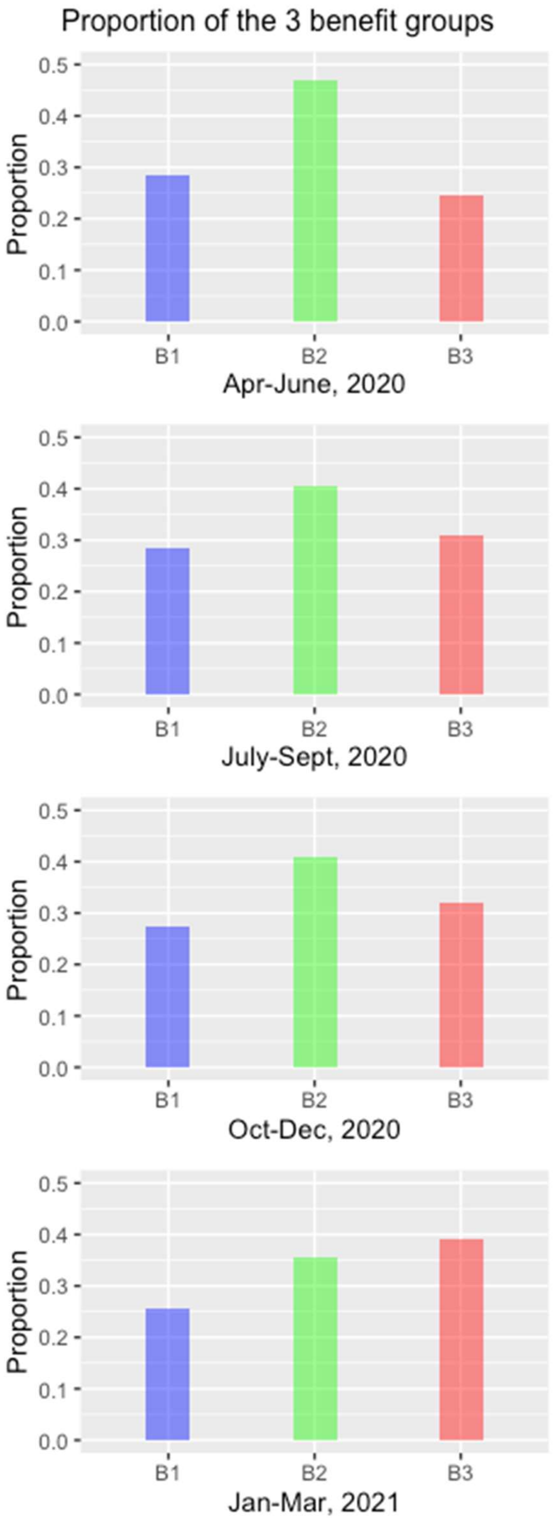


© 2022 Park H et al. *JAMA Network Open.*

eTable 21. Baseline Patient Characteristics for the Subgroup of Patients Who Would and Would Not Be Advised to Receive CCP, Determined by the Expanded TBI

For categorical variables each column sums to 100%

| Patient Characteristics | Subgroup mean (SD) or proportion in % | | Difference in mean divided by pooled-SD | Test for difference in means (proportions) | |
|---|---|---|---|---|---|
| | CCP advised (B) (n=1316) | CCP not advised (NB) (n=971) | | T-statistic | P-value ∗(significant) |
| Age in years | 62.24(14.8) | 57.69(15.3) | 0.30 | 7.17 | <10-2 * |
| Sex (proportion) | | | | | |
| Female | 33 % | 39 % | -0.11 | -2.56 | 0.01 * |
| Male | 67 % | 61 % | 0.11 | 2.56 | 0.01 * |
| Baseline WHO (proportion) | | | | | |
| 4 | 34 % | 0 % | 0.95 | 766.43 | <10-2 * |
| 5 | 52 % | 77 % | -0.55 | 262.31 | <10-2 * |
| 6 | 14 % | 23 % | -0.22 | 65.57 | <10-2 * |
| Blood type (proportion) | | | | | |
| O | 42 % | 52 % | -0.20 | -4.77 | <10-2 * |
| A | 39 % | 26 % | 0.26 | 6.26 | <10-2 * |
| B | 15 % | 18 % | -0.10 | -2.47 | 0.01 * |
| AB | 5 % | 3 % | 0.08 | 1.79 | 0.07 |
| Diabetes (proportion) | | | | | |
| Yes | 43 % | 20 % | 0.51 | 12.01 | <10-2 * |
| Pulmonary disease (proportion) | | | | | |
| Yes | 15 % | 7 % | 0.25 | 5.84 | <10-2 * |
| Cardiovascular (proportion) | | | | | |
| Yes | 69 % | 6 % | 1.63 | 38.6 | <10-2 * |
| Enrollment quarter (proportion) | | | | | |
| April – June 2020 | 32 % | 21 % | 0.24 | 5.73 | <10-2 * |
| July – September 2020 | 20 % | 19 % | 0.04 | 1.01 | 0.31 |
| October – December 2020 | 35 % | 42 % | -0.15 | -3.57 | <10-2 * |
| January – March 2021 | 13 % | 18 % | -0.14 | -3.34 | <10-2 * |
| Days since symptoms (proportion) | | | | | |
| 0 to 3 | 13 % | 12 % | 0.03 | 0.71 | 0.48 |
| 4 to 6 | 35 % | 36 % | -0.03 | -0.64 | 0.52 |
| 7 to 10 | 35 % | 36 % | -0.02 | -0.58 | 0.56 |
| 11 to 14 | 12 % | 10 % | 0.08 | 1.81 | 0.07 |
| More than 14 | 5 % | 6 % | -0.04 | -0.94 | 0.35 |

© 2022 Park H et al. *JAMA Network Open.*

## eAppendix 20. CCP Efficacy in the 3 Benefit Groups With Respect to All 6 Outcomes

After identifying the TBI subgroups associated with three levels of benefit – B1, B2 and B3 – we evaluated the efficacy of CCP with respect to all six outcomes separately within each group. Specifically, identical Bayesian analyses were performed as those used for evaluating the efficacy of CCP in the entire sample, see the main COMPILE results[16]. Within each of the 3 benefit-level groups defined by the Expanded TBI, eTable 22 describes the posterior distributions of the CCP efficacy odds ratios (ORs) using the median, the $2.5^{th}$ percentile and the $97.5^{th}$ percentile (i.e., the 95% credible interval) and the posterior probabilities that the CCP efficacy OR is less than 1 (P(OR<1)) and less than 0.8 (P(OR<0.8)) (see the next page for an explanation of these posterior probabilities).

Here we give an explanation about how evidence from the Bayesian posterior distributions of the odds ratios should be interpreted and how this interpretation contrasts with the more common frequentist reporting of evidence in terms of p-values.

A probability distribution of an OR gives the ability to estimate the likelihood that the OR lies in any given interval. The posterior distribution characterizes the OR after the data are observed. The posterior probability of the OR allows us to compute, for example, that the OR is less than 1 [P(OR<1]. An OR<1 indicates that there is a desirable effect from the treatment with CCP. The posterior probability for OR <1 [P(OR<1)] is the likelihood that CCP has any desirable effect; for example, P(OR<1) > 90% provides strong evidence for CCP efficacy. However, with a large sample size, this posterior probability can increase considerably, even if the median of the OR is very close to 1; for example, median OR=0.93. To ensure that beyond the presence of any effect, i.e., that the effect is large enough to be clinically meaningful, a useful characteristic of the posterior probability distribution is the quantity P(OR<0.8). An OR=0.8 means that the odds for undesirable outcome under the control treatment are reduced by 20% with CCP. Such an effect can be considered more than a minimal effect. A P(OR<0.8) ε50% indicates that it is more likely than not that the CCP desirable effect is more than minimal. Combined, the conditions P(OR<1) ε 90% and P(OR<0.8) ε 50% constitute strong evidence for more than a minimal effect of CCP.

Bayesian credible intervals (CrIs) convey different information than frequentist confidence intervals (CIs). A frequentist confidence interval refers to confidence in the analytic procedure (see below). The Bayesian credible interval refers to the likelihood that the effect of interest (here the OR for CCP vs. control) lies within a particular interval. Therefore, the forest plots (displayed in eFigure 14) should not be interpreted in the same way as frequentist CIs.

Next, we provide more discussion on the difference between Bayesian credible intervals (CrIs) and frequentist confidence intervals (CIs). For illustration we focus on 95% intervals. A frequentist 95% CI either includes or excludes the true but unknown effect (e.g., OR for CCP vs. control), and the probability that it includes the true but unknown OR is 95%. The confidence in the procedure means that if we repeated the procedure many times (i.e., perform the same experiment, collect data, fit the models we used, and estimate the OR and the 95% frequentist CIs), 95% of those CI will contain the true OR. For any given experiment, we cannot know whether our particular 95% CI contains the true OR or not, but we are confident in the procedure that we used, because under repeated experimentation, 95% of the CIs would cover the true OR. On the other hand, a 95% Bayesian credible interval tells us that, given the data we observed, the likelihood that the OR is in the interval (a, b) is 95%: this statement incorporates the uncertainty about the distribution of the OR of interest, given the prior information and data.

---

[16] AB Troxel et al., Effect of COVID-19 Convalescent Plasma on Clinical Status in Hospitalized Patients: A Real-Time Individual Patient Data Meta-Analysis of Randomized Clinical Trials, *JAMA* (under review)

© 2022 Park H et al. *JAMA Network Open.*

Another contrast between the Bayesian and frequentist frameworks for making inferences and statements about uncertainty is the following. P-values indicate the likelihood of the data given the (null) hypothesis, i.e., how likely it is for what we observed to happen, if the null hypothesis (OR=1) were true? In other words, the p-value is a conditional **probability of the data, given a hypothesis**. A Bayesian posterior probability, on the other hand, assesses the **probability of a hypothesis given the data**. For example, P(OR<1) is the conditional probability that CCP has an effect (i.e., OR<1) given the observed data. Combined with the discussion above, having both P(OR < 1) ε 90% and P(OR < 0.8) ε 50% constitutes strong evidence for more than minimal efficacy of CCP against control.

The results in eTable 22 indicate that, with respect to all six outcomes, the benefit group B1 (identified from the Expanded TBI) has both P(OR < 1) ε 90% and P(OR < 0.8) ε 50%. This suggests strong likelihood for the group B1 for more than minimal efficacy of CCP against control with respect to all six outcomes. Patients in B2 are also expected to benefit, but the posterior probability of the CCP efficacy is not as strong as that of B1. Finally, the patients in B3, with P(OR < 1) < 30% for all six outcomes (see eTable 22), are unlikely to benefit from the CCP treatment.

eFigure 14 below displays the medians and the 95% CrIs for the ORs provided in eTable 22. Note that the 2.5% and the 97.5% of the posterior distributions reported in Table 4.3 are the lower and the upper boundaries of the 95% CrIs for the ORs, respectively.

© 2022 Park H et al. *JAMA Network Open.*

# eTable 22. Posterior Probability Distributions of the ORs of CCP vs Control for All Outcomes, by Benefit Group Determined From the Expanded TBI

The 2.5th percentile and the 97.5th percentile are the lower and upper boundaries of the 95% credible intervals. Bayesian cumulative proportional odds models are used for the ordinal WHO scores and Bayesian logistic regression models are used for the binary outcomes (WHO ≥7 and WHO =10), adjusted for age, sex, baseline WHO scores, duration of symptoms prior to treatment, diabetes, cardiovascular disease, pulmonary disease and quarter of enrollment.

| Outcome | TBI | 2.5% | **Median** | 97.5% | P(OR<1) | P(OR<0.8) |
|---|---|---|---|---|---|---|
| WHO score at day 14 (ordinal) | Group B1 (n=629) | 0.486 | **0.694** | 1.061 | 0.957 | 0.766 |
| | Group B2 (n=953) | 0.611 | **0.823** | 1.119 | 0.901 | 0.425 |
| | Group B3 (n=705) | 1.140 | **1.582** | 2.173 | 0.005 | 0.000 |
| | Overall (n=2292) | 0.731 | **0.936** | 1.194 | 0.718 | 0.093 |
| WHO ≥ 7 at day 14 (binary) | Group B1 (n=629) | 0.352 | **0.562** | 1.022 | 0.971 | 0.900 |
| | Group B2 (n=953) | 0.534 | **0.815** | 1.274 | 0.816 | 0.463 |
| | Group B3 (n=705) | 0.896 | **1.611** | 2.616 | 0.051 | 0.012 |
| | Overall (n=2292) | 0.682 | **0.926** | 1.288 | 0.688 | 0.175 |
| WHO = 10 at day 14 (binary) | Group B1 (n=629) | 0.367 | **0.628** | 1.103 | 0.950 | 0.810 |
| | Group B2 (n=953) | 0.584 | **0.945** | 1.557 | 0.588 | 0.247 |
| | Group B3 (n=705) | 0.690 | **1.170** | 1.944 | 0.276 | 0.071 |
| | Overall (n=2292) | 0.606 | **0.877** | 1.264 | 0.768 | 0.307 |
| WHO score at day 28 (ordinal) | Group B1 (n=629) | 0.513 | **0.729** | 1.134 | 0.929 | 0.680 |
| | Group B2 (n=953) | 0.595 | **0.801** | 1.123 | 0.918 | 0.494 |
| | Group B3 (n=705) | 1.106 | **1.564** | 2.199 | 0.008 | 0.000 |
| | Overall (n=2290) | 0.744 | **0.936** | 1.187 | 0.722 | 0.086 |
| WHO ≥ 7 at day 28 (binary) | Group B1 (n=629) | 0.324 | **0.517** | 0.952 | 0.981 | 0.933 |
| | Group B2 (n=953) | 0.553 | **0.829** | 1.268 | 0.813 | 0.431 |
| | Group B3 (n=705) | 0.979 | **1.677** | 2.624 | 0.029 | 0.005 |
| | Overall (n=2290) | 0.670 | **0.908** | 1.238 | 0.736 | 0.202 |
| WHO = 10 at day 28 (binary) | Group B1 (n=629) | 0.359 | **0.575** | 0.983 | 0.978 | 0.901 |
| | Group B2 (n=953) | 0.515 | **0.801** | 1.244 | 0.839 | 0.499 |
| | Group B3 (n=705) | 0.835 | **1.406** | 2.273 | 0.093 | 0.016 |
| | Overall (n=2290) | 0.615 | **0.847** | 1.184 | 0.843 | 0.360 |

## eFigure 14. Forest Plots of Median ORs and 95% Credible Intervals From the Posterior Probability Distributions for the ORs by Benefit Groups Determined From the Expanded TBI for All 6 Outcomes

The posterior distributions are estimated from the Bayesian cumulative proportional odds models for the ordinal WHO scores and logistic regression, adjusted for age, sex, baseline WHO scores, duration of symptoms prior to treatment, diabetes, cardiovascular disease, pulmonary disease and quarter of enrollment.

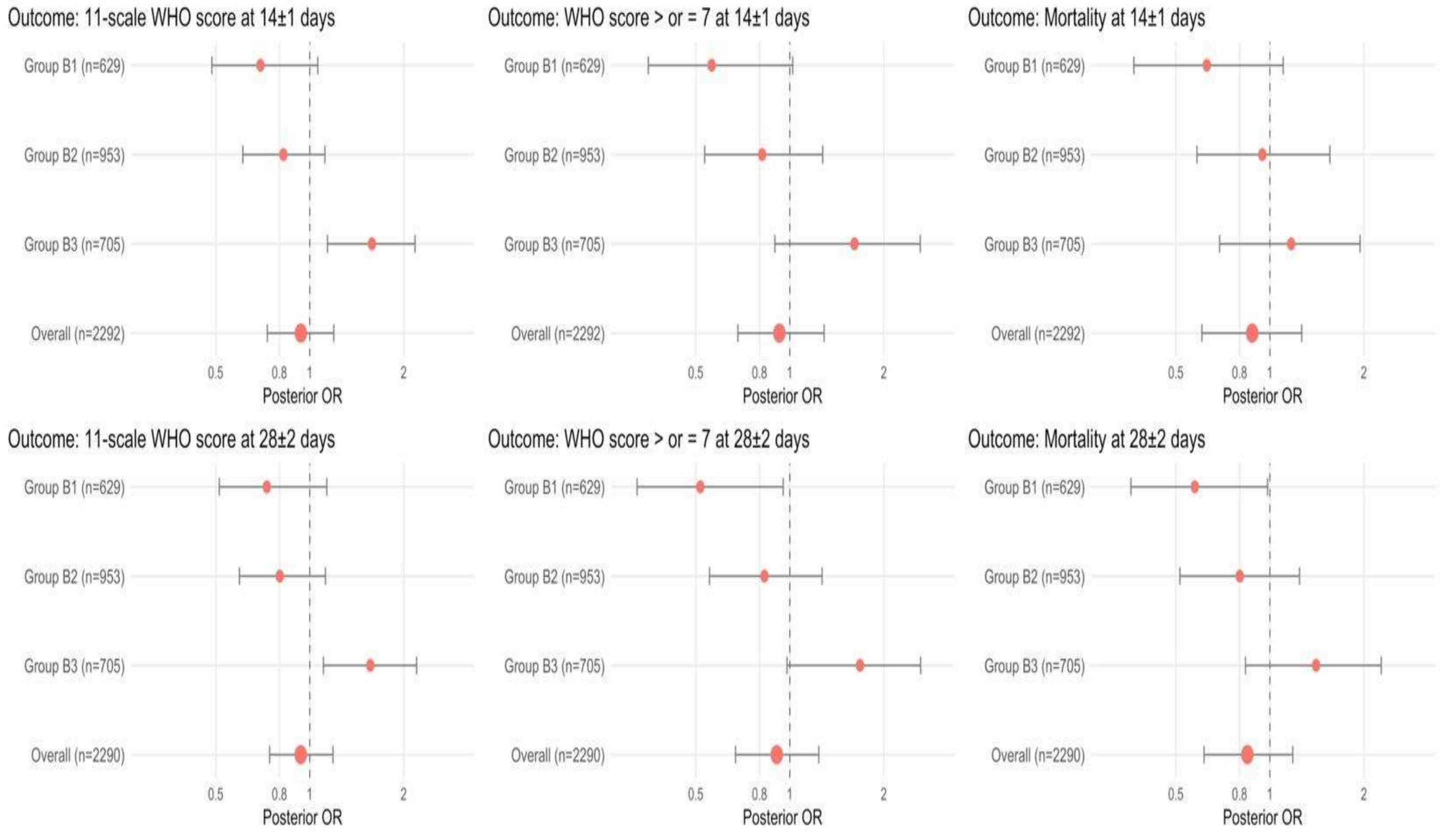


© 2022 Park H et al. *JAMA Network Open.*

## eAppendix 21. Differential Treatment Effect by Benefit Levels, in Comparison With Differential Treatment Effect by Single Patient Characteristics

eFigures 15-20 below contrast the moderating effect of the Expanded TBI to that of the baseline variables prespecified in the COMPILE protocol. We conducted a set of subgroup analyses for CCP effect moderations by levels of the TBI. The results show strong CCP effect moderations by levels of the TBI, in terms of the magnitude contrast (i.e., the CCP efficacy OR difference) between the subgroup expected to most benefit (B1) and that expected to not benefit (B3), compared to the contrast based on the other individual baseline characteristics. This strong moderation exhibited by the TBI suggests the advantage of using the TBI for guiding clinical decisions over the baseline patient characteristics individually. Although the results were displayed in terms of only the 3 discrete benefit levels (i.e., B1, B2 and B3), the TBI, as it continuously ranges from 0-1, provides a generally continuous gradation of the expected benefit from CCP, which can be useful in clinical practice in quantifying the treatment benefit from CCP for an individual subject.

© 2022 Park H et al. *JAMA Network Open.*

eFigure 15. Odds Ratios and 95% Credible Intervals for Subgroup-Specific Odds for Individual Baseline Covariates and 3 Benefit Groups for WHO Score on Day 14

Under the expanded covariates-adjusted Bayesian model employed in the main COMPILE analysis.

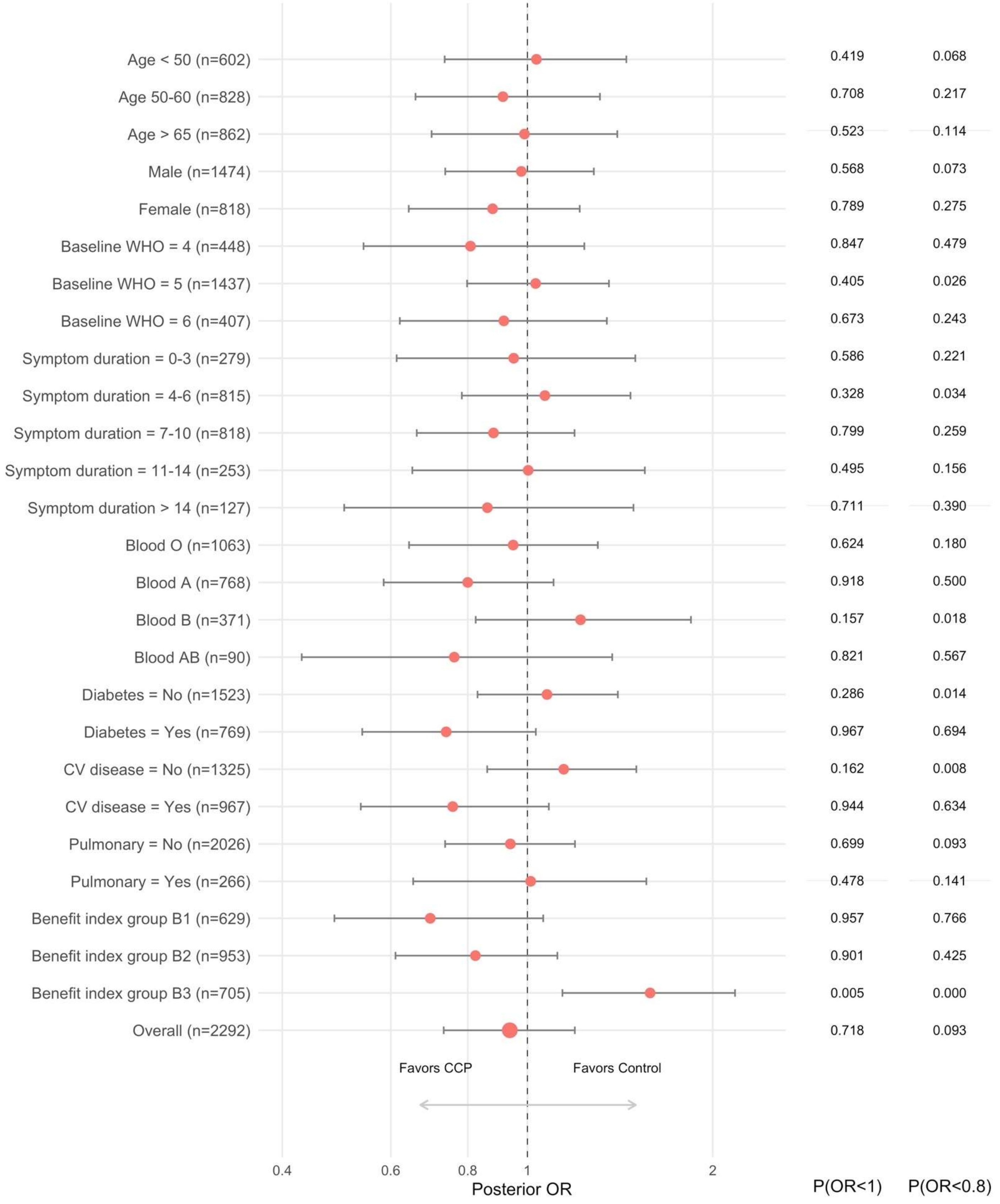


© 2022 Park H et al. *JAMA Network Open.*

## eFigure 16. Odds Ratios and 95% Credible Intervals for Subgroup-Specific Odds for Individual Baseline Covariates and 3 Benefit Groups for WHO Score 7 to 10 on Day 14

Under the expanded covariates-adjusted Bayesian model employed in the main COMPILE analysis.

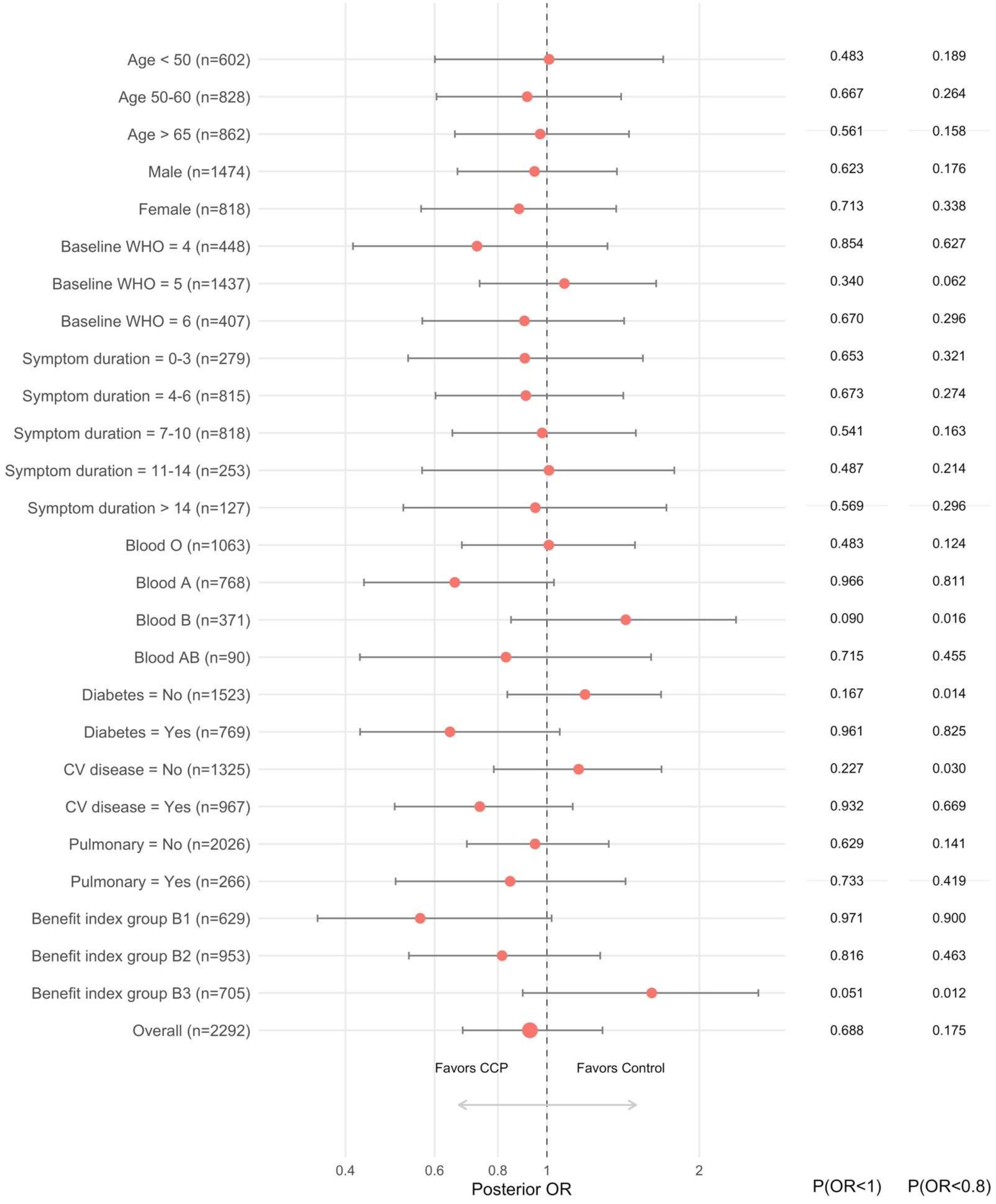


© 2022 Park H et al. *JAMA Network Open.*

eFigure 17. Odds Ratios and 95% Credible Intervals for Subgroup-Specific Odds for Individual Baseline Covariates and 3 Benefit Groups for WHO Score on Day 28

Under the expanded covariates-adjusted Bayesian model employed in the main COMPILE analysis.

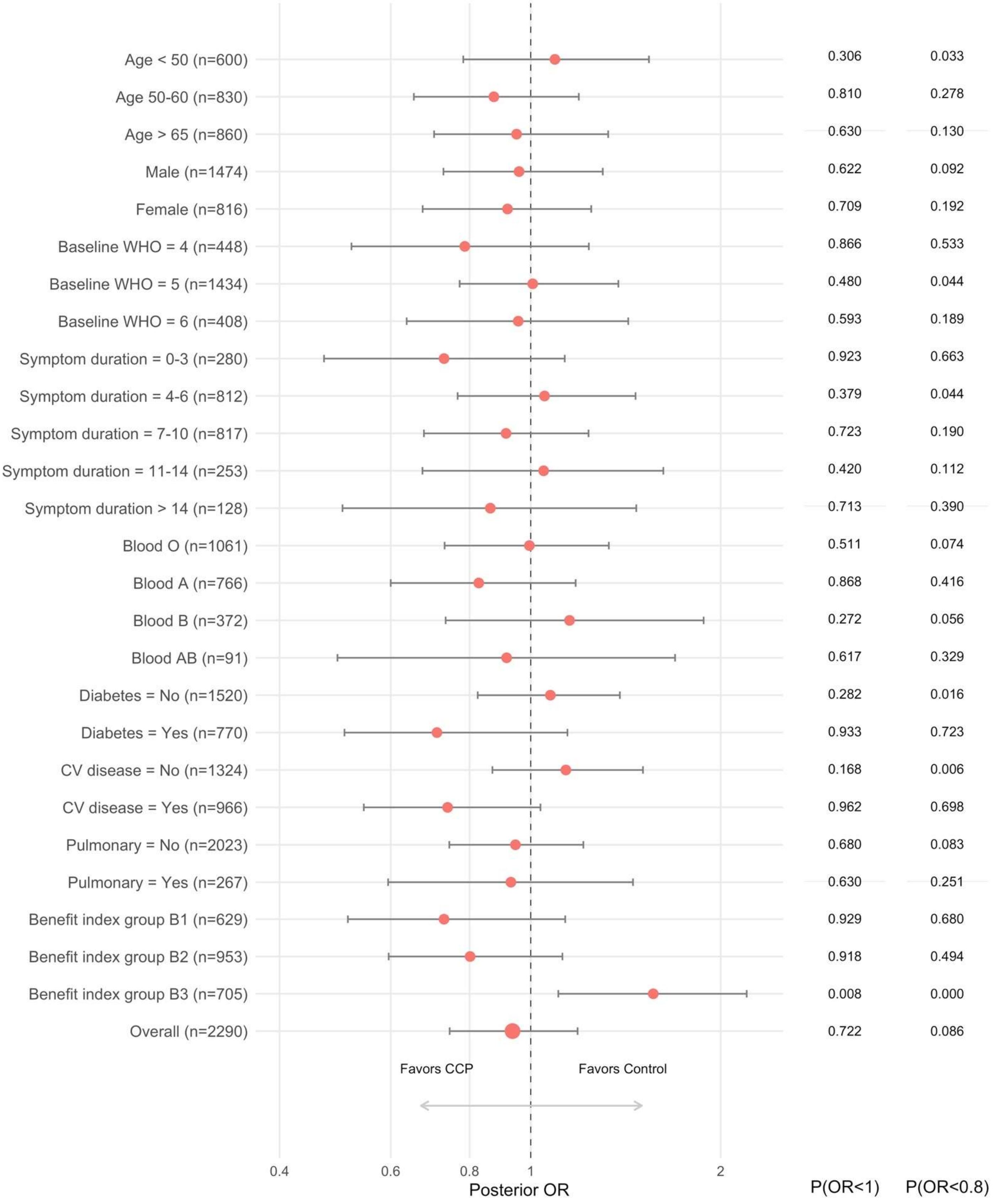


© 2022 Park H et al. *JAMA Network Open.*

## eFigure 18. Odds Ratios and 95% Credible Intervals for Subgroup-Specific Odds for Individual Baseline Covariates and 3 Benefit Groups for WHO Score 7 to 10 on Day 28

Under the expanded covariates-adjusted Bayesian model employed in the main COMPILE analysis.

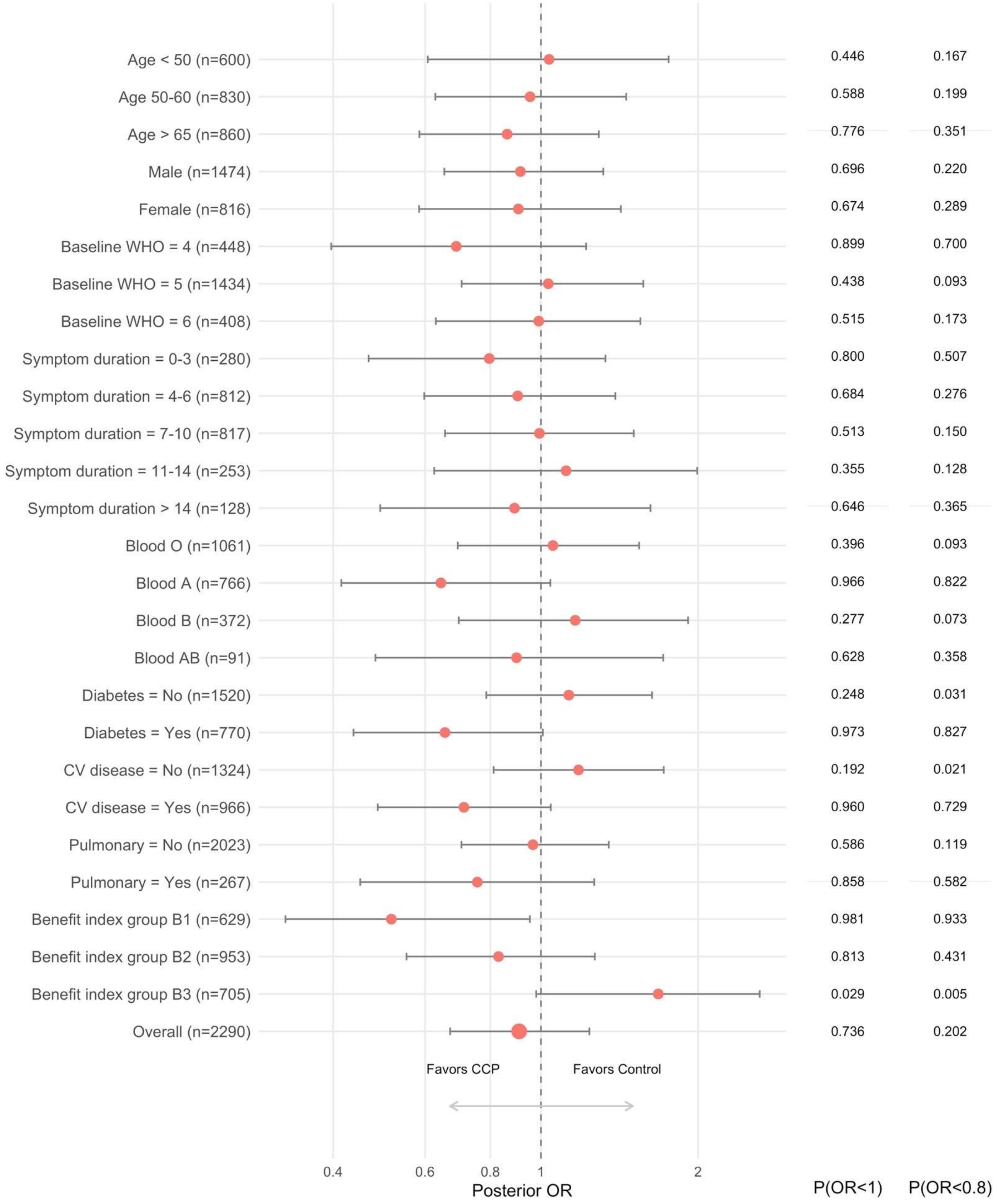


© 2022 Park H et al. *JAMA Network Open.*

eFigure 19. Odds Ratios and 95% Credible Intervals for Subgroup-Specific Odds for Individual Baseline Covariates and 3 Benefit Groups for Mortality on Day 14

Under the expanded covariates-adjusted Bayesian model employed in the main COMPILE analysis.

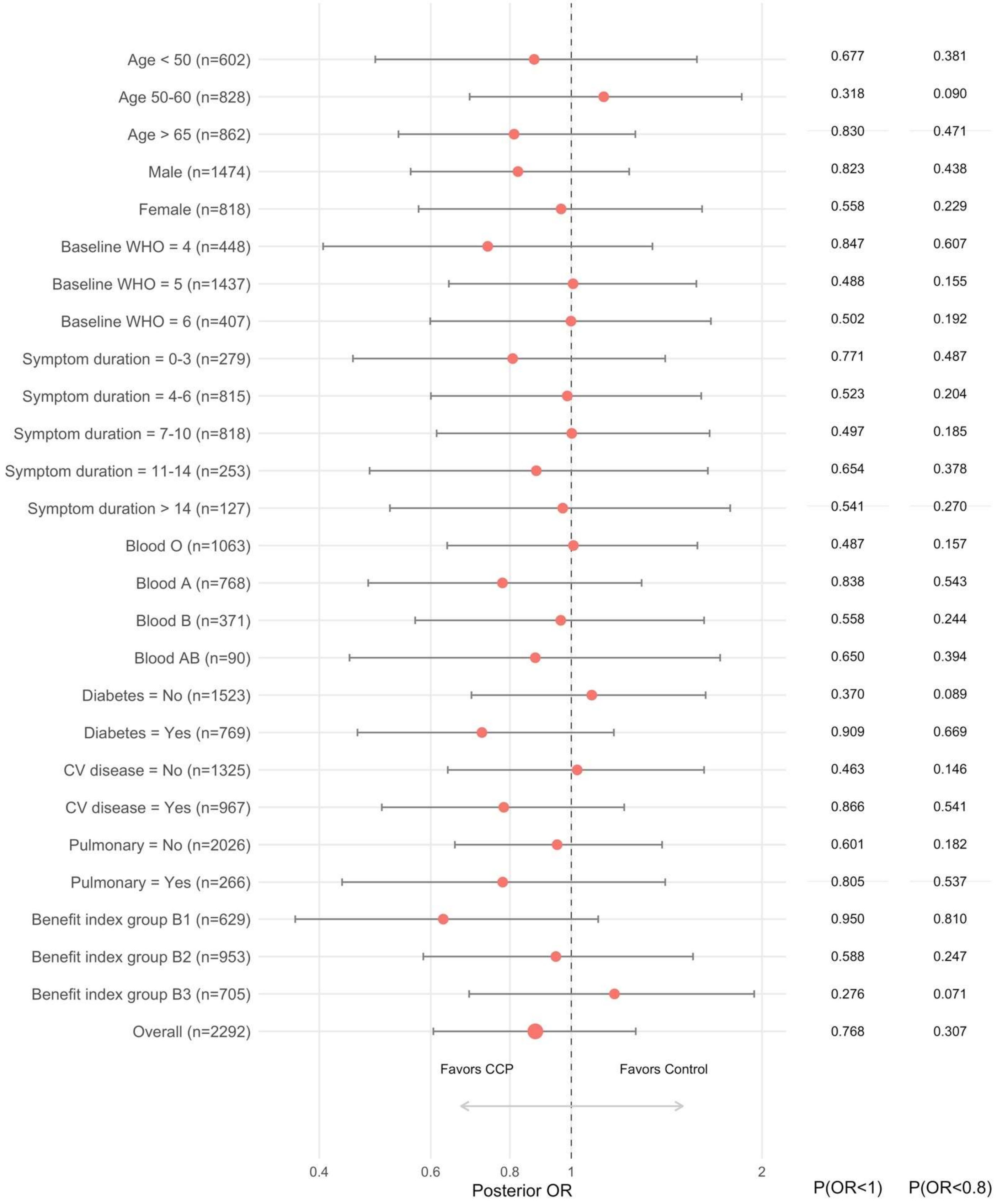


© 2022 Park H et al. *JAMA Network Open.*

## eFigure 20. Odds Ratios and 95% Credible Intervals for Subgroup-Specific Odds for Individual Baseline Covariates and 3 Benefit Groups for Mortality on Day 28

Under the expanded covariates-adjusted Bayesian model employed in the main COMPILE analysis.

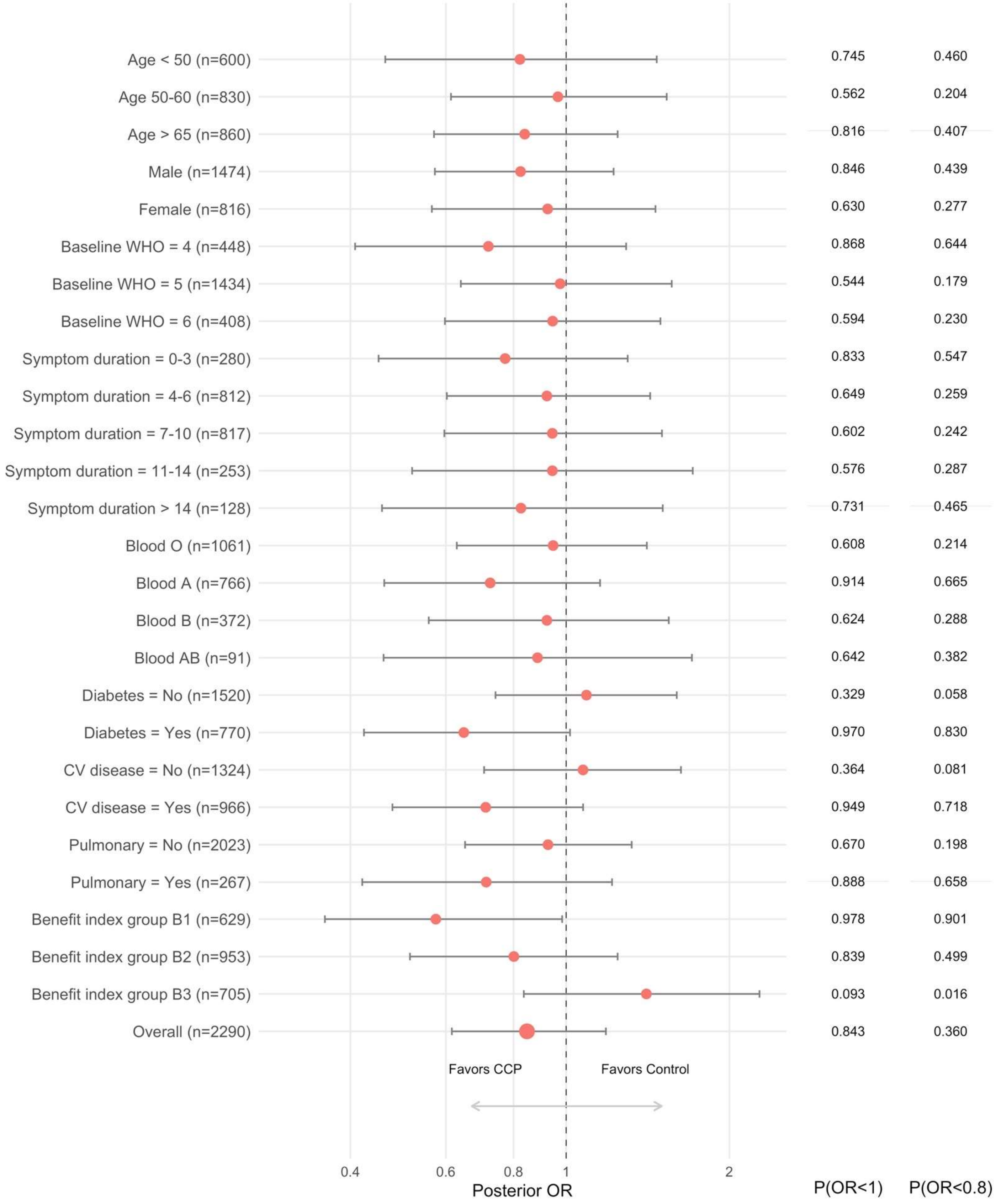


© 2022 Park H et al. *JAMA Network Open.*

## **eAppendix 22.** CCP Benefit as a Function of the Basic TBI

In eFigure 21 below, unadjusted CCP efficacy odds ratios for all six outcomes as functions of the Basic TBI are displayed. All six odd ratios show a monotonically decreasing trend with the basic TBI. Thus, larger values of TBI correspond to larger benefit from CCP across all outcomes (as in the expanded TBI). For the Basic TBI, the proportion of patients falling in the benefit groups B1, B2 and B3 were 26%, 38% and 35%, respectively. The same cut-points (0.2, 0.4) for groupings (B1, B2 and B3) are used for all panels of Figure 22 and in all analyses that follow.

# eFigure 21. CCP Benefit as a Function of the Basic TBI

Efficacy is assessed with respect to all six outcomes and it is measured in terms of odds ratios (ORs). The ORs for the ordinal WHO outcomes are estimated from cumulative proportional odds models. The ORs for the remaining binary outcomes are estimated logistic regressions. ORs less than 1 indicate superior outcome with CCP compared to control. The TBI is developed on the ordinal WHO outcome at day 14. The cut-points (a, b) for groups B1, B2, and B3 are (0.2, 0.4), and are the same for all panels.

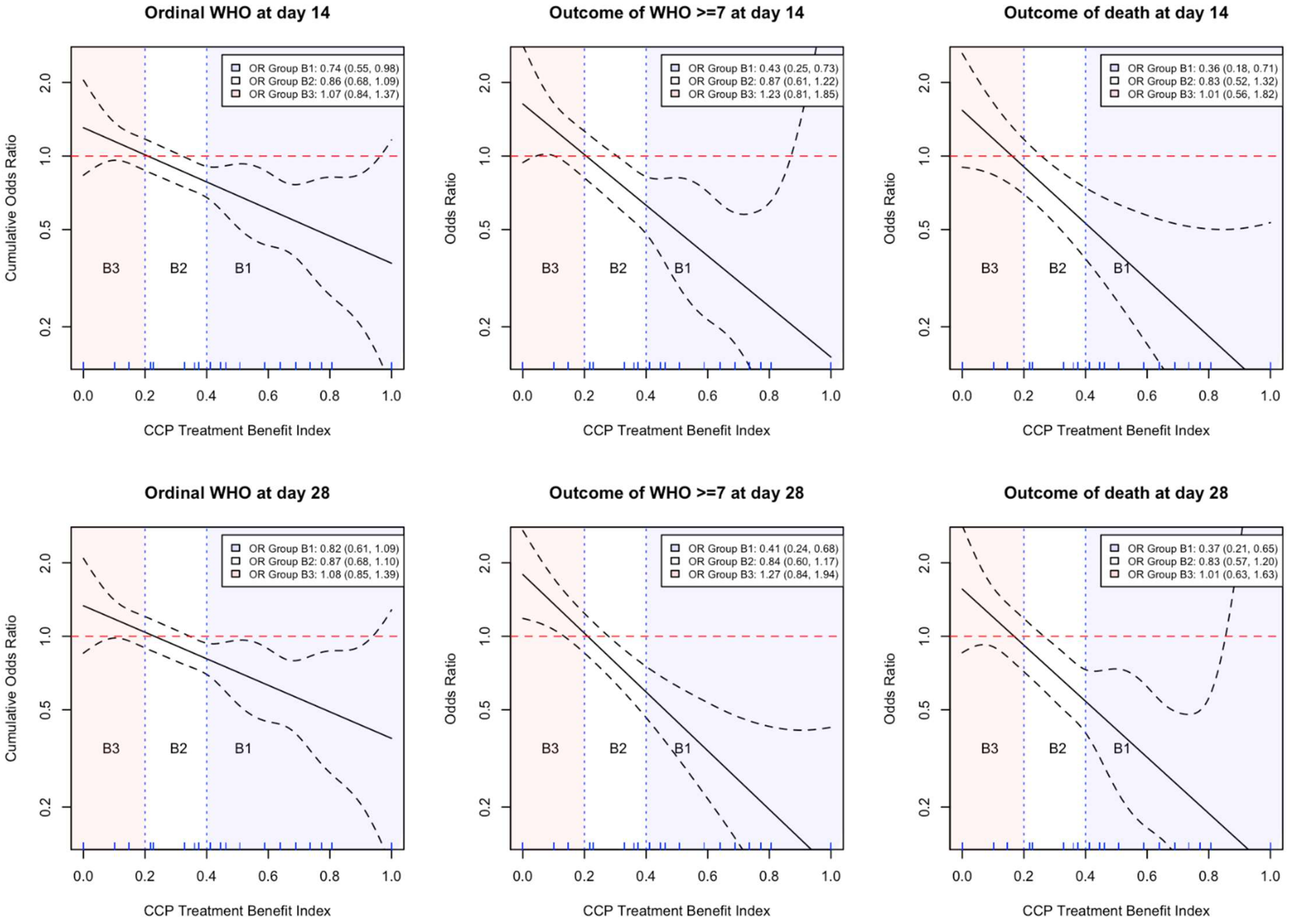


Although there are clear monotone relationships, the relationships between the Basic TBI and the CCP efficacy odds ratios appear to be less robust compared to those of the Expanded TBI (presented in Figure 1 of the main manuscript), as suggested by wider confidence intervals.

© 2022 Park H et al. *JAMA Network Open.*

## eAppendix 23. Baseline Patient Characteristics by Benefit Levels Determined From the Basic TBI

eTable 23 describes the baseline characteristics of patients in the three benefit-level groups (B1, B2 and B3) defined from the Basic TBI. For each benefit group, frequentist's unadjusted OR estimates (not adjusted for any covariates) and the associated 95% confidence intervals are also provided in the bottom of the table.

In eTable 23, in the rows associated with the enrollment quarters, we only reported the distribution of the patient population across the enrollment quarters within each benefit-level group (i.e., each column sums to 1). In eFigure 22 below, we display the distribution of the patient population across the different benefit groups (B1, B2 and B3) identified by the Basic TBI, by each quarter. We do this to investigate whether the patient population has shifted over time and its possible relationship with the observed shift in CCP efficacy presented in eFigure 2. In eFigure 22, a notable observation is that the proportion of patients in B1 decreases over time, with B1, the group predicted to have the most benefit from CCP, having the largest prevalence of patients enrolled the early period of the pandemic, i.e., between April and June 2021. This partly explains the observed CCP efficacy that decreased over time in the COMPILE study.

Additionally, we provide the binary subgroup characteristics where the subgroups were identified based on the CCP efficacy OR of 1 (OR≥1 or OR<1), under the TBI model (S1.3), i.e., the subgroup of the patients who were predicted to benefit (B) and the subgroup of the patients who were not predicted to benefit (NB) from the CCP treatment, predicted by the Basic TBI.

© 2022 Park H et al. *JAMA Network Open.*

## eTable 23. Baseline Characteristics of COMPILE Patients in the 3 Benefit-Level Groups Defined by the Basic TBI

For categorical variables each column sums to 100%). In the bottom of the table, we also provide the frequentist subgroup-specific CCP efficacy OR estimates and the associated 95% confidence intervals, for all six outcomes.

| Patient Characteristics | Overall mean (SD) or proportion (n=2287) | Subgroup mean (SD) or proportion | | | Test for difference in means (proportions) | |
|---|---|---|---|---|---|---|
| | | Group B1 (n=597) | Group B2 (n=879) | Group B3 (n=811) | F-statistic | P-value ∗(significant) |
| Age in years | 60.31(15.2) | 56.61(14.4) | 65.57(14.4) | 57.33(15.0) | 93.07 | <10-2 * |
| Sex (proportion) | | | | | | |
| Female | 36 % | 31 % | 35 % | 39 % | 5.63 | <10-2 * |
| Male | 64 % | 69 % | 65 % | 61 % | 5.63 | <10-2 * |
| Baseline WHO (proportion) | | | | | | |
| 4 | 20 % | 75 % | 0 % | 0 % | 2514.8 | <10-2 * |
| 5 | 63 % | 10 % | 71 % | 93 % | 976.04 | <10-2 * |
| 6 | 18 % | 15 % | 29 % | 7 % | 74.91 | <10-2 * |
| Blood type (proportion) | | | | | | |
| O | 46 % | 42 % | 48 % | 47 % | 3.06 | 0.05 |
| A | 33 % | 31 % | 37 % | 32 % | 4.48 | 0.01 * |
| B | 16 % | 23 % | 11 % | 17 % | 18.66 | <10-2 * |
| AB | 4 % | 5 % | 4 % | 4 % | 0.61 | 0.54 |
| Diabetes (proportion) | | | | | | |
| Yes | 34 % | 47 % | 38 % | 19 % | 72.65 | <10-2 * |
| Pulmonary disease (proportion) | | | | | | |
| Yes | 12 % | 21 % | 10 % | 7 % | 36.2 | <10-2 * |
| Cardiovascular (proportion) | | | | | | |
| Yes | 42 % | 39 % | 83 % | 0 % | 1250.7 | <10-2 * |
| Enrollment quarter (proportion) | | | | | | |
| April – June 2020 | 27 % | 48 % | 18 % | 22 % | 95.01 | <10-2 * |
| July – September 2020 | 20 % | 23 % | 20 % | 17 % | 3.58 | 0.03 * |
| October – December 2020 | 38 % | 22 % | 44 % | 43 % | 45.64 | <10-2 * |
| January – March 2021 | 15 % | 7 % | 18 % | 18 % | 19.96 | <10-2 * |
| Days since symptoms (proportion) | | | | | | |
| 0 to 3 | 12 % | 10 % | 15 % | 11 % | 3.85 | 0.02 * |
| 4 to 6 | 36 % | 31 % | 38 % | 36 % | 4.44 | 0.01 * |
| 7 to 10 | 36 % | 37 % | 33 % | 38 % | 1.99 | 0.14 |
| 11 to 14 | 11 % | 16 % | 9 % | 10 % | 9.94 | <10-2 * |
| More than 14 | 6 % | 7 % | 5 % | 5 % | 0.81 | 0.45 |
| CCP efficacy OR (95% CI) | Overall | B1 | B2 | B3 | | |
| OR for day 14 WHO ordinal | 0.89(0.77,1.03) | 0.74(0.55,0.98) | 0.86(0.68,1.09) | 1.07(0.84,1.37) | | |
| OR for day 14 WHO ≥ 7 | 0.85(0.67,1.07) | 0.43(0.25,0.73) | 0.87(0.61,1.22) | 1.23(0.81,1.85) | | |
| OR for day 14 WHO = 10 | 0.72(0.52,0.98) | 0.36(0.18,0.71) | 0.83(0.52,1.32) | 1.01(0.56,1.82) | | |
| OR for day 28 WHO ordinal | 0.92(0.80,1.07) | 0.82(0.61,1.09) | 0.87(0.68,1.10) | 1.08(0.85,1.39) | | |
| OR for day 28 WHO ≥ 7 | 0.83(0.66,1.04) | 0.41(0.24,0.68) | 0.84(0.60,1.17) | 1.27(0.84,1.94) | | |
| OR for day 28 WHO = 10 | 0.73(0.57,0.95) | 0.37(0.21,0.65) | 0.83(0.57,1.20) | 1.01(0.63,1.63) | | |

© 2022 Park H et al. *JAMA Network Open.*

eFigure 22. Proportions of the 3 Benefit Groups (B1, B2 and B3) Identified From the Basic TBI, by Quarter

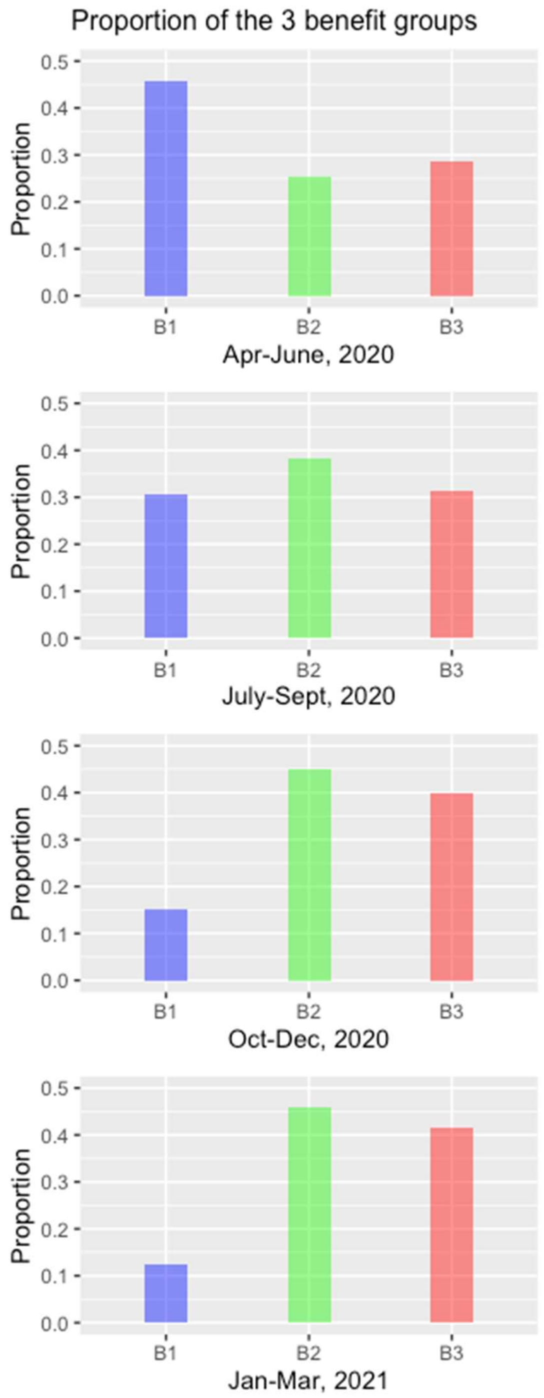


© 2022 Park H et al. *JAMA Network Open.*

## eTable 24. Baseline Patient Characteristics for the Subgroup of Patients Who Would and Would Not Be Advised to Receive CCP, Determined by the Basic TBI

In the bottom of the table, we also provide the frequentist subgroup-specific CCP efficacy OR estimates with respect to all six outcomes and the associated 95% confidence intervals.

| Patient Characteristics | Subgroup mean (SD) or proportion in % | | Difference in mean divided by pooled-SD | Test for difference in means (proportions) | |
|---|---|---|---|---|---|
| | CCP advised (B) (n=1222) | CCP not advised (NB) (n=1065) | | T-statistic | P-value ∗(significant) |
| Age in years | 62.17(14.6) | 58.17(15.6) | 0.27 | 6.33 | <10-2 * |
| Sex (proportion) | | | | | |
| Female | 33 % | 38 % | -0.11 | -2.67 | 0.01 * |
| Male | 67 % | 62 % | 0.11 | 2.67 | 0.01 * |
| Baseline WHO (proportion) | | | | | |
| 4 | 37 % | 0 % | 1.04 | 24.77 | <10-2 * |
| 5 | 56 % | 71 % | -0.31 | -7.42 | <10-2 * |
| 6 | 8 % | 29 % | -0.59 | -14.1 | <10-2 * |
| Blood type (proportion) | | | | | |
| O | 45 % | 48 % | -0.04 | -1.04 | 0.30 |
| A | 35 % | 32 % | 0.06 | 1.48 | 0.14 |
| B | 16 % | 17 % | -0.03 | -0.71 | 0.48 |
| AB | 4 % | 4 % | 0.02 | 0.41 | 0.68 |
| Diabetes (proportion) | | | | | |
| Yes | 44 % | 22 % | 0.49 | 11.75 | <10-2 * |
| Pulmonary disease (proportion) | | | | | |
| Yes | 16 % | 7 % | 0.27 | 6.45 | <10-2 * |
| Cardiovascular (proportion) | | | | | |
| Yes | 70 % | 10 % | 1.53 | 36.58 | <10-2 * |
| Enrollment quarter (proportion) | | | | | |
| April – June 2020 | 33 % | 20 % | 0.29 | 6.98 | <10-2 * |
| July – September 2020 | 20 % | 19 % | 0.02 | 0.42 | 0.67 |
| October – December 2020 | 34 % | 42 % | -0.17 | -4.10 | <10-2 * |
| January – March 2021 | 13 % | 18 % | -0.15 | -3.50 | <10-2 * |
| Days since symptoms (proportion) | | | | | |
| 0 to 3 | 13 % | 11 % | 0.06 | 1.40 | 0.16 |
| 4 to 6 | 35 % | 36 % | -0.03 | -0.69 | 0.49 |
| 7 to 10 | 35 % | 37 % | -0.05 | -1.14 | 0.26 |
| 11 to 14 | 12 % | 10 % | 0.09 | 2.11 | 0.03 |
| More than 14 | 5 % | 6 % | -0.04 | -1.07 | 0.28 |
| | | | | | |

| CCP efficacy Odds Ratio (95% CI) | CCP advised (B) | CCP not advised (NB) |
|---|---|---|
| OR for day 14 WHO ordinal | 0.77(0.63,0.94) | 1.06(0.86,1.31) |
| OR for day 14 WHO ≥ 7 | 0.56(0.39,0.80) | 1.15(0.84,1.58) |
| OR for day 14 WHO = 10 | 0.45(0.28,0.73) | 1.05(0.68,1.62) |
| OR for day 28 WHO ordinal | 0.79(0.64,0.96) | 1.10(0.89,1.37) |
| OR for day 28 WHO ≥ 7 | 0.52(0.37,0.73) | 1.22(0.89,1.67) |
| OR for day 28 WHO = 10 | 0.47(0.32,0.69) | 1.07(0.75,1.53) |

© 2022 Park H et al. *JAMA Network Open.*

## eAppendix 24. CCP Efficacy in the 3 Benefit Groups With Respect to All 6 Outcomes, Determined by the Basic TBI

Within each of the 3 groups defined by the Basic TBI, eTable 25 characterizes the posterior distributions of the odds ratios using the 2.5$^{th}$ percentile, the median, and the 97.5$^{th}$ percentile (i.e., the 95% credible interval) and the posterior probabilities that the odds ratio is less than 1 (P(OR<1) and less than 0.8 (P(OR<0.8).

The results in eTable 25 indicate that, with respect to all six outcomes the benefit group B1 (identified from the Basic TBI) has $P(OR < 1) \geq 90\%$ and $P(OR < 0.8) \geq 75\%$, suggesting strong evidence for more than minimal efficacy of CCP against control for the group with respect to all six outcomes.

eFigure 23 displays the medians and the 95% CrIs for the ORs in eTable 25. The 2.5% and the 97.5% of the posterior distributions reported in eTable 25 are the lower and the upper boundaries of the 95% CrIs for the ORs, respectively.

## eTable 25. Posterior Probability Distributions of the Odds Ratios of CCP vs Control for All Outcomes, by Benefit Group Determined From the Basic TBI

The 2.5th percentile and the 97.5th percentile are the lower and upper boundaries of the 95% credible intervals. Bayesian cumulative proportional odds models are used for the ordinal WHO scores and Bayesian logistic regression models are used for the binary outcomes (WHO ≥7 and WHO =10), adjusted for age, sex, baseline WHO scores, duration of symptoms prior to treatment, diabetes, cardiovascular disease, pulmonary disease and quarter of enrollment.

| Outcome | Benefit Group | 2.5% | **Median** | 97.5% | P(OR<1) | P(OR<0.8) |
|---|---|---|---|---|---|---|
| WHO score at day 14 (ordinal) | Group B1 (n=597) | 0.440 | **0.642** | 0.964 | 0.983 | 0.870 |
| | Group B2 (n=879) | 0.693 | **0.926** | 1.271 | 0.700 | 0.163 |
| | Group B3 (n=811) | 1.008 | **1.373** | 1.864 | 0.022 | 0.001 |
| | Overall (n=2292) | 0.731 | **0.936** | 1.194 | 0.718 | 0.093 |
| WHO ≥ 7 at day 14 (binary) | Group B1 (n=597) | 0.330 | **0.552** | 0.953 | 0.982 | 0.913 |
| | Group B2 (n=879) | 0.621 | **0.929** | 1.421 | 0.640 | 0.232 |
| | Group B3 (n=811) | 0.893 | **1.402** | 2.183 | 0.075 | 0.007 |
| | Overall (n=2292) | 0.682 | **0.926** | 1.288 | 0.688 | 0.175 |
| WHO = 10 at day 14 (binary) | Group B1 (n=597) | 0.460 | **0.680** | 1.208 | 0.927 | 0.757 |
| | Group B2 (n=879) | 0.671 | **0.912** | 1.247 | 0.727 | 0.198 |
| | Group B3 (n=811) | 0.948 | **1.310** | 1.828 | 0.050 | 0.002 |
| | Overall (n=2292) | 0.606 | **0.877** | 1.264 | 0.768 | 0.307 |
| WHO score at day 28 (ordinal) | Group B1 (n=597) | 0.334 | **0.549** | 0.997 | 0.976 | 0.907 |
| | Group B2 (n=879) | 0.596 | **0.899** | 1.379 | 0.701 | 0.286 |
| | Group B3 (n=811) | 0.902 | **1.440** | 2.270 | 0.059 | 0.010 |
| | Overall (n=2290) | 0.744 | **0.936** | 1.187 | 0.722 | 0.086 |
| WHO ≥ 7 at day 28 (binary) | Group B1 (n=597) | 0.375 | **0.639** | 1.132 | 0.940 | 0.789 |
| | Group B2 (n=879) | 0.593 | **0.948** | 1.493 | 0.592 | 0.240 |
| | Group B3 (n=811) | 0.675 | **1.224** | 2.102 | 0.238 | 0.076 |
| | Overall (n=2290) | 0.670 | **0.908** | 1.238 | 0.736 | 0.202 |
| WHO = 10 at day 28 (binary) | Group B1 (n=597) | 0.345 | **0.577** | 1.043 | 0.967 | 0.873 |
| | Group B2 (n=879) | 0.585 | **0.880** | 1.338 | 0.719 | 0.324 |
| | Group B3 (n=811) | 0.696 | **1.222** | 2.098 | 0.236 | 0.066 |
| | Overall (n=2290) | 0.615 | **0.847** | 1.184 | 0.843 | 0.360 |

© 2022 Park H et al. *JAMA Network Open.*

eFigure 23. Forest Plots of Median Odds Ratios and 95% Credible Intervals From the Posterior Probability Distributions for the Odds Ratios by Benefit Groups Determined From the Basic TBI for All 6 Outcomes

The posterior distributions are estimated from the Bayesian cumulative proportional odds models for the ordinal WHO scores and logistic regression, adjusted for age, sex, baseline WHO scores, duration of symptoms prior to treatment, diabetes, cardiovascular disease, pulmonary disease and quarter of enrollment.

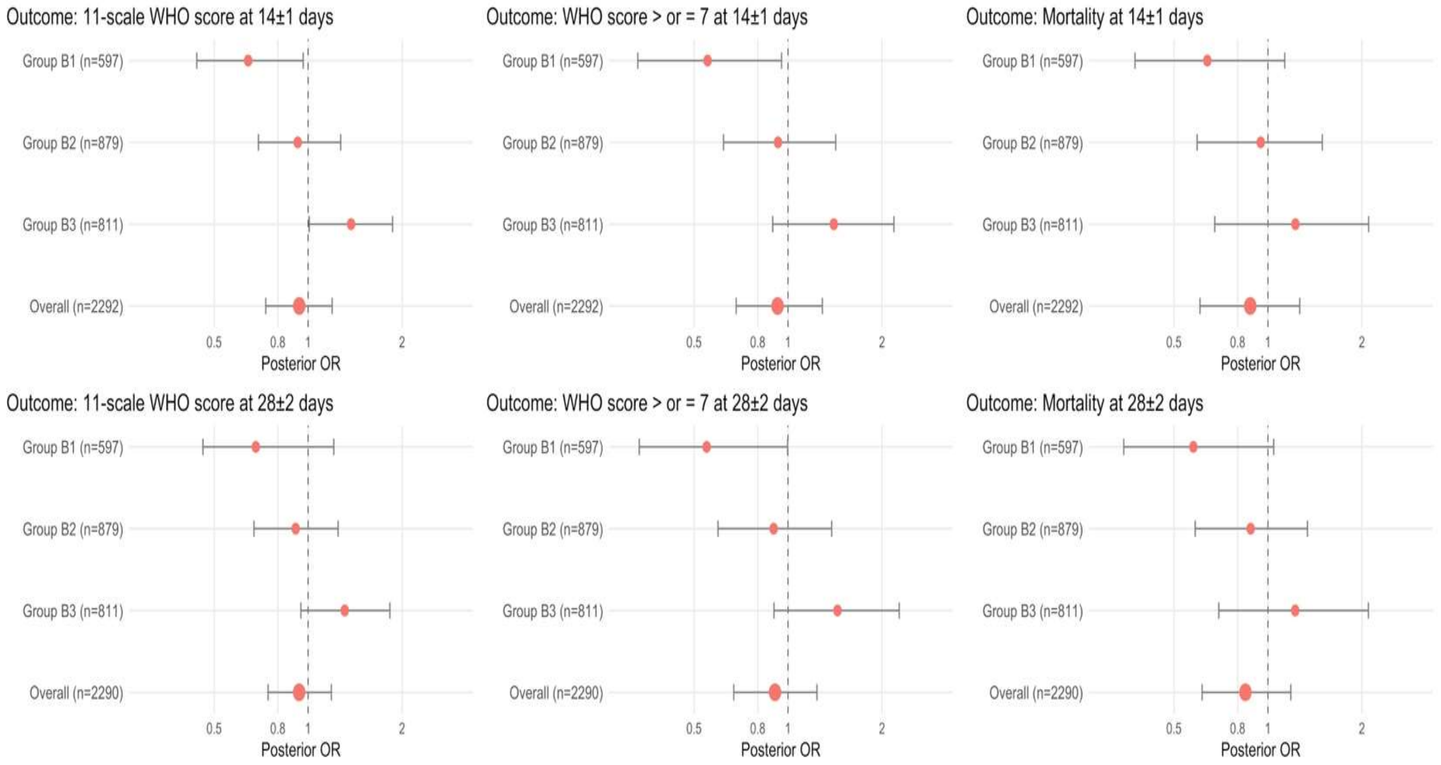


© 2022 Park H et al. *JAMA Network Open.*

## eAppendix 25. Kaplan-Meier Plots for Time to Death and Time to Hospital Discharge, With Log-Rank Test for Time to Death and Gray Competing Risk Test for Time to Hospital Discharge, Basic TBI

Unlike the results from the Expanded TBI (provided in Figure 2 of the main text), in the competing risk analysis results presented in eFigure 24, the group B3 identified by the Basic TBI does not exhibit a meaningful difference between the CCP and control effects, in terms of time to death and time to hospital discharge.

© 2022 Park H et al. *JAMA Network Open.*

## eFigure 24. Comparison Between CCP and Control Patients for Time to Death and Time to Hospital Discharge by Benefit Level, Determined by the Basic TBI

The top panel shows the Kaplan-Meier curves for time to death (the two decreasing trajectories in the panel) and time to hospital discharge (the two increasing trajectories in the panel) for group B1, and the middle and bottom panels show the same results for groups B2 and B3, respectively. Results from frequentist inferences from stratified log-rank and the Gray's tests are shown in the individual panel.

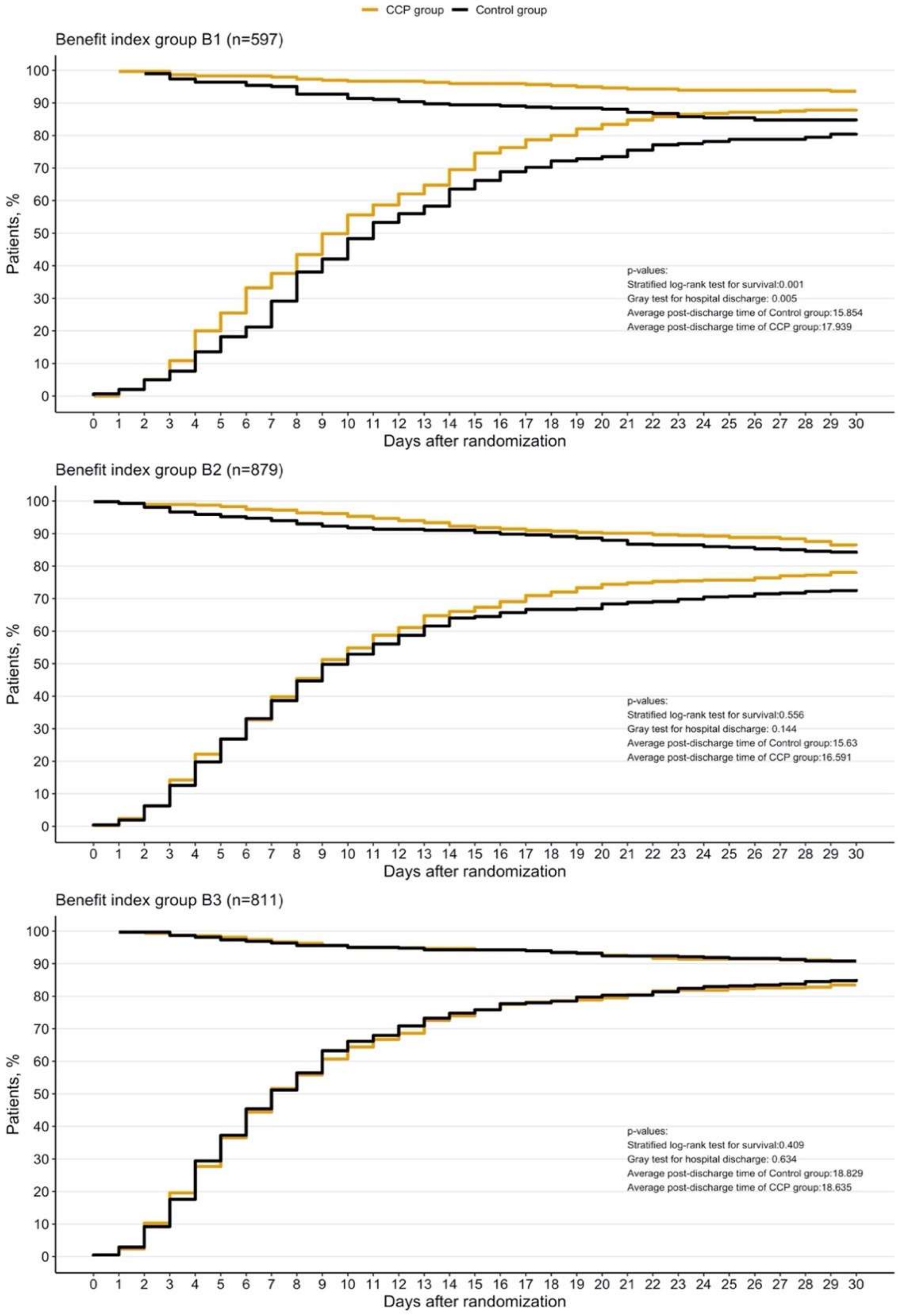


© 2022 Park H et al. *JAMA Network Open.*

## eAppendix 26. Validation on Expanded Access Program (EAP) Data

Description of the EAP validation cohort

A single-arm Expanded Access Program (EAP) sponsored by the Mayo Clinical was established to provide access to CCP for hospitalized patients with COVID-19[17]. For subjects treated under EAP, the neutralization titers in the donor CCP were measured post-hoc, which indicated presence of SARS-CoV-2 antibody titers[18]. There were 8,698 EAP subjects (there were 5303 subjects who received high-titers CCP, as qualified by a live viral neutralization titer of ≥ 1:250) who had no missing information for neutralization titer in the transfused CCP and the following covariates: quarter (or month) of treatment, age, sex, diabetes indicator, pulmonary disease indicator, cardiovascular disease indicator, blood type, WHO baseline score (ranging from 4 to 6 on a 0-10 scale), and a known mortality status at day 28 (which was the only available outcome from the study), see eTable 26 below for baseline characteristics of this EAP validation cohort.

Approach

The EAP sample contains only CCP treated subjects. A matching of the EAP sample with a cohort of control-treated subjects from COMPILE was performed. Considering evidence that outcomes improved over the course of the pandemic independently of known patient risk factors and that no EAP patients were enrolled after September 2020, the time period of the analysis was restricted to April 2020 through September 2020. There were 546 control patients in COMPILE treated from April 2020 through September 2020 that were available for matching. This gave the set of 546 control-treated and 8698 CCP-treated subjects, a total of n=9244 patients in the sample, which was available for matching. Since blood type information was available for the whole study sample, both the Basic and Expanded TBI scores could be computed and tested.

*Matching results*
Coarsened exact matching was used to provide matched cohorts, in which the continuous covariate (age) was coarsened into 5-year bins, and a complete cross of the coarsened covariates is used to form subclasses defined by each combination of the coarsened covariate levels. The matching was performed via R package MatchIt[19]. This resulted in an effective sample size of the control-treated subjects n = 212 and of the CCP-treated subjects n =1896 in the matched set, see eTable 27 for the summary of balance of the matched data.

We also considered matching of the COMPILE control-treated subjects with the high titer EAP subjects only, using the same coarsened exact matching procedure, which resulted in a high titers matched set consisting of an effective sample size of n=1145 CCP-treated patients and n=197 control-treated, see eTable 28 below for the results (summary of balance) of the matched data.

*Primary analysis*
TBIs provide binary subgroups: the group predicted to benefit from treatment with CCP (group B) or the group not predicted to benefit from treatment with CCP (group NB). As a primary analysis, the TBI would be considered to be validated, if OR(B) < OR(NB).

*Sensitivity analysis*
As a sensitivity analysis (sensitivity with respect to different partitioning of benefit levels), the TBI would be considered to be validated, if the CCP efficacy ORs were ordered across the three benefit groups: OR(B1) <

---

[17] Senefeld JW, Johnson PW, Kunze KL et al. Program and patient characteristics for the United States Expanded Access Program to COVID-19 convalescent plasma. *medRxiv* 2021.04.08.21255115; doi: https://doi.org/10.1101/2021.04.08.21255115

[18] Joyner MJ, Carter RE, Senefeld JW. *et al*. Convalescent Plasma Antibody Levels and the Risk of Death from Covid-19, 2021, *NEJM*, 384 (11), pp 1015-1027, doi: 10.1056/NEJMoa2031893

[19] Ho DE, Imai K, King G, Stuart EA (2011). MatchIt: Nonparametric Preprocessing for Parametric Causal Inference. *Journal of Statistical Software*, **42**(8), 1–28. https://www.jstatsoft.org/v42/i08/.

© 2022 Park H et al. *JAMA Network Open.*

OR(B2) < OR(B3). As another sensitivity analysis (sensitivity with respect to different definition of CCP treatment), we also considered the case where the analysis was restricted to the patients who received high titers CCP (as qualified by a live viral neutralization titer of ≥ 1:250).

*Determining the groups with different expected benefit*

To obtain the B and NB and the B1, B2 and B3 groups associated with different levels of benefit, we computed the TBI scores of all patients in the matched sets. Using the prespecified cut-points (see eTable 14), we partitioned the patients in the matched sets into the B and NB and the B1, B2 and B3 groups.

To compute the subgroup specific ORs within each benefit subgroup, we performed a subgroup-specific logistic regression with just the treatment indicator as a covariate, using the weights according to the subclasses formed during matching (i.e., the matching weights) in computing the OR. These subgroup-specific ORs are then compared with one another to validate the relationship between the TBIs and the CCP efficacy.

Results

eTable 29 below shows the distribution of the subjects in the matched data across levels of benefit (two or three levels of benefit) defined by the Basic TBI or the Expanded TBI, respectively, and depending on whether partitioning was restricted to the patients with high titers CCP or not.

eTable 30 below reports the results (i.e., the subgroup-specific ORs) of this validation analysis. The results of the primary analysis are highlighted.

Both the Basic TBI and the Expanded TBI were validated with respect to the two-levels of benefit subgroups (showing the relationship OR(B) < OR(NB)), for both the primary analysis using all EAP patients (reported in the highlighted cells in the 3rd and 5th columns) and the sensitivity analysis using the high titer EAP patients only (reported in the 4th and 6th columns). Also, with respect to the three-levels of benefit subgroups, the Expanded TBI was validated (showing the relationship OR(B1) < OR(B2) < OR(B3)), in both cases of the all EAP patients matching and the high titers CCP patients matching. However, with respect to the three-levels of benefit subgroups, the Basic TBI was not validated, in both cases of the all EAP patients matching and the high titers CCP patients matching. As the Basic TBI does not incorporate blood type in the determination of the index, this may indicate the relevance/importance of blood type information for determination of the CCP benefit levels.

© 2022 Park H et al. *JAMA Network Open.*

## eTable 26. Baseline Characteristics for Participants Treated Under the EAP With Available Data for All Specified Parameters

"CCP (All)" corresponds to the group of patients who received any CCP, and "CCP (High titer)" corresponds to the group of patients who received high titers CCP (as qualified by a live viral neutralization titer of ≥ 1:250).

| | CCP (All) | CCP (High titer) |
|---|---|---|
| n | 8,698 | 5,303 |
| Age (median, IQR) | 61.6 [50.0, 72.6] | 61.3 [49.8, 72.4] |
| Female sex (n, %) | | |
| Male | 5,058 (58.2) | 3047 (57.5) |
| Female | 3,611 (41.5) | 2237 (42.2) |
| NA | 29 (0.3) | 19 (0.4) |
| Baseline WHO severity (n, %) | | |
| 4 - hospitalized/ no $O_2$ | 478 (5.5) | 287 (5.4) |
| 5 - hospitalized/ $O_2$ by mask or nasal | 3971 (45.7) | 2473 (46.6) |
| 6 - hospitalized/ $O_2$ by non-invasive ventilation | 4249 (48.9) | 2543 (48.0) |
| NA | 0 | 0 |
| Blood group (n, %) | | |
| O | 4417 (50.8) | 2737 (51.6) |
| A | 2937 (33.8) | 1760 (33.2) |
| B | 1061 (12.2) | 643 (12.1) |
| AB | 283 (3.3) | 163 (3.1) |
| NA | 0 | 0 |
| History of diabetes (n, %) | | |
| No | 5338 (61.4) | 3269 (61.6) |
| Yes | 3360 (38.6) | 2034 (38.4) |
| NA | 0 | 0 |
| History of pulmonary disease (n, %) | | |
| No | 7339 (84.4) | 4486 (84.6) |
| Yes | 1359 (15.6) | 817 (15.4) |
| NA | 0 | 0 |
| History of cardiovascular disease (n, %) | | |
| No | 4619 (53.1) | 2787 (52.6) |
| Yes | 4079 (46.9) | 2516 (47.4) |
| NA | 0 | 0 |
| Enrollment quarter (n, %) | | |
| Apr-June 2020 | 5338 (61.4) | 3301 (62.2) |
| July-Sept 2020 | 3360 (38.6) | 2002 (37.8) |
| Oct-Dec 2020 | 0 | 0 |
| Jan-Mar 2021 | 0 | 0 |

## eTable 27. Summary of Balance for Matched Data for EAP

| | Mean CCP Treated (effective n= 1896) | Mean Control (effective n= 212) | Std. Mean Diff. |
|---|---|---|---|
| Age | 59.889 | 60.143 | -0.016 |
| Sex | 0.333 | 0.333 | 0.000 |
| Diabetes | 0.271 | 0.271 | 0.000 |
| Pulmonary disease | 0.054 | 0.054 | 0.000 |
| Cardiovascular disease | 0.399 | 0.399 | 0.000 |
| Blood type A | 0.340 | 0.340 | 0.000 |
| Blood type B | 0.066 | 0.066 | 0.000 |
| Blood type AB | 0.004 | 0.004 | -0.000 |
| Blood type O | 0.590 | 0.590 | 0.000 |
| Baseline WHO =4 | 0.066 | 0.066 | -0.000 |
| Baseline WHO =5 | 0.664 | 0.664 | 0.000 |
| Baseline WHO =6 | 0.270 | 0.270 | 0.000 |
| Enrollment quarter 2 | 0.576 | 0.576 | 0.000 |
| Enrollment quarter 3 | 0.424 | 0.424 | 0.000 |

© 2022 Park H et al. *JAMA Network Open.*

## eTable 28. Summary of Balance for Matched Data for EAP, High Titer Only

| | Mean CCP Treated (effective n= 1145) | Mean Control (effective n= 197) | Std. Mean Diff. |
|---|---|---|---|
| Age | 58.994 | 59.277 | -0.018 |
| Sex | 0.332 | 0.332 | 0.000 |
| Diabetes | 0.253 | 0.253 | 0.000 |
| Pulmonary disease | 0.054 | 0.054 | 0.000 |
| Cardiovascular disease | 0.397 | 0.397 | 0.000 |
| Blood type A | 0.330 | 0.330 | 0.000 |
| Blood type B | 0.066 | 0.066 | 0.000 |
| Blood type AB | 0.001 | 0.001 | 0.000 |
| Blood type O | 0.603 | 0.603 | 0.000 |
| Baseline WHO =4 | 0.064 | 0.064 | 0.000 |
| Baseline WHO =5 | 0.669 | 0.669 | -0.000 |
| Baseline WHO =6 | 0.267 | 0.267 | 0.000 |
| Enrollment quarter 2 | 0.592 | 0.592 | 0.000 |
| Enrollment quarter 3 | 0.408 | 0.408 | -0.000 |

© 2022 Park H et al. *JAMA Network Open.*

eTable 29. Participants in the Matched Data, by Benefit Levels Defined by the Expanded and the Basic TBIs and Depending on Whether All EAP Patients or Only Those Treated With High-Titer Plasma Were Used For Validation

| Levels of benefit | Group | Basic TBI | | Expanded TBI | |
|---|---|---|---|---|---|
| | | Matching of all EAP patients | Matching of high titer EAP patients only | Matching of all EAP patients | Matching of high titer EAP patients only |
| | | % (n) | % (n) | % (n) | % (n) |
| Two levels of benefit | B | 40.0 (1,387) | 40.2 (605) | 47.1 (1,088) | 46.8 (705) |
| | NB | 60.0 (925) | 59.8 (900) | 52.9 (1,224) | 53.2 (800) |
| Three levels of benefit | B1 | 16.0 (371) | 16.2 (224) | 20.9 (483) | 21.2 (319) |
| | B2 | 40.6 (939) | 39.8 (599) | 37.4 (864) | 36.5 (550) |
| | B3 | 43.3 (1,002) | 44.0 (662) | 41.7 (965) | 42.3 (636) |

© 2022 Park H et al. *JAMA Network Open.*

## eTable 30. CCP Benefit Odds Ratios Within Different Benefit Levels Defined by the Basic and the Expanded TBIs and Depending on Whether Subgrouping Was Restricted to Patients with High-Titer CCP

The group-specific ORs were computed from the matched data, where matching of the control-treated subjects was either to all EAP patients (reported in the 3rd and 5th columns) or to only EAP patients with high titers CCP (reported in the 4th and 6th columns).

| Levels of benefit | Group | Basic TBI | | Expanded TBI | |
|---|---|---|---|---|---|
| | | Matching of all EAP patients | Matching of high titer EAP patients only | Matching of all EAP patients | Matching of high titer EAP patients only |
| | | OR (95% CI) | OR (95% CI) | OR (95% CI) | OR (95% CI) |
| Two levels of benefit | B | 0.50 (0.33, 0.76) | 0.52 (0.33, 0.83) | 0.52 (0.36, 0.75) | 0.55 (0.36, 0.82) |
| | NB | 0.95 (0.64, 1.41) | 0.99 (0.63, 1.54) | 1.11 (0.70, 1.77) | 1.12 (0.67, 1.88) |
| Three levels of benefit | B1 | 0.40 (0.21, 0.76) | 0.44 (0.21, 0.91) | 0.41 (0.24, 0.71) | 0.48 (0.27, 0.88) |
| | B2 | 0.87 (0.58, 1.30) | 0.93 (0.59, 1.47) | 0.71 (0.45, 1.12) | 0.72 (0.43, 1.21) |
| | B3 | 0.69 (0.41, 1.17) | 0.65 (0.36, 1.16) | 1.09 (0.65, 1.81) | 1.06 (0.60, 1.85) |

© 2022 Park H et al. *JAMA Network Open.*

## eAppendix 27. Validation on an Emergency Use Authorization (EUA) Study

Description of the EUA validation cohort

Use of CCP was permitted outside of RCTs under the Emergency Use Authorization (EUA) as of August 2020[20]. Patients at Montefiore Medical Center (MMC) received CCP under this EUA from October 2020 to March 2021. Patients received 1 unit of high titer CCP obtained from the New York Blood Center that met the criteria defined by the FDA on the Ortho-Clinical Diagnostics VITROS Anti-SARS-CoV-2 IgG platform (S/C ≥ 12) or 2 units considered low titer (having a S/C <12)[21] until February 4, 2021, when the FDA updated their guidelines to limit the authorization to the high titer CCP (defined by S/C >9.5)[22].

The demographics and clinical course of n=216 adult CCP recipients who received CCP from October 2020 to March 2021 were studied retrospectively (see eTable 31 below for baseline characteristics of the patients). According to FDA guidance, CCP was mainly used in patients considered to have early COVID-19 based on the assessment of an infectious diseases physician, lack of or non-invasive oxygen supplementation, and patients with B cell immunodeficiency. Eligibility was determined when patients were referred by the primary team physician. Clinical, laboratory, and patient outcome data were collected through chart review from the electronic health record under Albert Einstein College of Medicine/MMC (Einstein/MMC) IRB protocol 2020-11426. De-identified data were provided to New York University with a data sharing agreement approved by Einstein/MMC and NYU Langone to validate the TBI.

Approach

Because there were no control-treated patients in the EUA study, we designed a validation analysis including all subjects from the EUA cohort compared to matched controls from the COMPILE cohort. Given that outcomes improved over the course of the pandemic independent of known patient risk factors and no EUA patients were enrolled before October 2020, we restricted the time of the analysis to October 2020-March 2021, so EUA and COMPILE patients could be selected from a concurrent time period. Therefore, the analysis included a control arm of COMPILE patients (n=551) enrolled in the 4$^{th}$ quarter of 2020 (October-December 2020) and the 1st quarter of 2021 (January-March 2021) and all EUA patients (n=210) who were not ventilated at time of treatment.

Unlike the EAP sample (n=8698) considered in Section eAppendix 25, the EUA study has only a small number of CCP-treated patients (n=210). Given this relatively small sample size, the coarsened exact matching used in Section eAppendix 25 limited the effective sample size for the CCP-treated patients of the EAP sample to only n=29. Thus, instead of using this line of exact matching, we assigned each treated patient in the EUA sample to a control unit as a match, one by one. Specifically, age, sex, diabetes indicator, pulmonary disease indicator, cardiovascular disease indicator, enrollment quarter and the baseline WHO score were used to compute a distance between each treated unit and each control unit, based on which the nearest neighbor matching was performed via R package MatchIt[23]. For 210 treated subjects of the EUA sample, 210 untreated subjects from

[20] US Food and Drug Administration. FDA issues Emergency Use Authorization for convalescent plasma as potential promising COVID-19 treatment, another achievement in administration's fight against pandemic. 2020.
[21] Recommendations for Investigational COVID-19 Convalescent Plasma tFaDAA, 2020. Available from: https://www.fda.gov/media/141480/download.
[22] US Food and Drug Administration. Convalescent Plasma EUA Letter of Authorization March 9, 2021. https://www.fda.gov/media/141477/download
[23] Ho DE, Imai K, King G, Stuart EA (2011). MatchIt: Nonparametric Preprocessing for Parametric Causal Inference. *Journal of Statistical Software*, **42**(8), 1–28. https://www.jstatsoft.org/v42/i08/.

© 2022 Park H et al. *JAMA Network Open.*

COMPILE were matched, resulting in a total of n=420 subjects in the matched sample. The summary of balance for these matched data is given in eTable 32.

Blood type information was available for the whole study sample, and thus both the Basic and Expanded TBI scores for all n=420 patients were able to be computed. These TBIs were used to stratify the sample (n=420) into 1) two groups: expected to benefit (B) and not expected to benefit (NB); and 2) three groups: large benefit (B1), moderate benefit (B2) and potential harm (B3) expected from the CCP treatment, using the cutoff points specified in eTable 14, for the Basic TBI and the expanded TBI, respectively. We then computed the subgroup-specific ORs. Although the 11-point WHO scale ordinal outcome at day 28 was not available for most of the EUA patients (65% missing), that at day 14 was available for all the subjects. Thus, we computed the subgroup-specific ORs with respect to the ordinal 11-point WHO outcome at day 14, using cumulative logistic regression, with the treatment indicator as the only covariate.

<u>Results</u>:

eTable 33 below shows the distribution of the subjects across levels of benefit (two or three levels of benefit) defined by the Basic TBI or the Expanded TBI, respectively.

eTable 34 shows the corresponding ORs. The results in eTable 34 indicate validation of the TBIs. The results of the primary analysis are highlighted.

Although evaluated on a small sample (external data set n=210), both the Basic and Expanded TBIs are considered externally validated, because specification for the patient subgroups were determined independently using the COMPILE data set and the respective ORs (for both the Basic TBI and the Expanded TBI) have the benefit relationships, OR(B) < OR(NB), and OR(B1) < OR(B2) < OR(B3), that were predicted by the TBIs.

© 2022 Park H et al. *JAMA Network Open.*

eTable 31. Baseline Characteristics of CCP Recipients at Montefiore Medical Center, Bronx, NY

| Characteristic | CCP recipients |
|---|---|
| Median age (IQR) – year | 69 (58 – 78) |
| Sex – no. (%) | |
| Female | 90 (41.7) |
| Male | 126 (58.3) |
| BMI[A], median (IQR) | 27.5 (23.8 – 31.3) |
| Ethnicity[B] – no. (%) | |
| Hispanic | 85 (39.4) |
| Non-Hispanic | 118 (54.6) |
| Unknown | 13 (6.0) |
| Race[B] – no. (%) | |
| White | 51 (23.6) |
| African American | 52 (24.1) |
| Asian | 6 (2.8) |
| Other | 72 (33.3) |
| Unknown | 35 (16.2) |
| Coexisting disorder – no. (%) | |
| Hypertension | 143 (66.2) |
| Hyperlipidemia | 53 (24.5) |
| Cardiovascular disease | 53 (24.5) |
| Pulmonary disease | 35 (16.2) |
| Chronic kidney disease | 31 (14.4) |
| End stage renal disease | 27 (12.5) |
| Diabetes | 82 (37.9) |
| Obesity (BMI ≥ 30) | 69 (31.9) |
| Immunocompromising condition | 80 (37.0) |
| ABO blood group | |
| Type A | 65 (30.1) |
| Type B | 37 (17.1) |
| Type O | 107 (49.5) |
| Type AB | 7 (3.2) |
| Quarter of enrollment | |
| October – December 2020 | 28 (12.9) |
| January – March 2021 | 188 (87.0) |
| No. of days between symptom onset and admission, median (IQR) | 4 (3 – 7) |
| No. of days between admission and transfusion, median (IQR) | 2 (1 – 3) |
| No. of days between symptom onset and transfusion, median (IQR) | 6 (4 – 8) |
| Baseline WHO 11-point ordinal scale for clinical improvement – no. (%) | |
| 4 – No oxygen therapy | 103 (47.7) |
| 5 – Oxygen by mask or nasal prongs | 80 (37.0) |
| 6 – Oxygen by NIV or High flow | 27 (12.5) |
| 7 – Intubation & Mechanical ventilation; pO2/FIO2 >/= 150 or SpO2/FIO2 >/=200 | 3 (1.4) |
| 8 – Mechanical ventilation pO2/FIO2 < 150 (SpO2/FIO2 <200) or vasopressors | 3 (1.4) |
| Pharmacologic interventions – no. (%) | |
| Corticosteroids (prior to plasma) | 137 (63.4) |
| Corticosteroids (anytime during hospitalization) | 147 (68.1) |
| Remdesivir (anytime during hospitalization) | 165 (76.4) |
| Laboratory values on the day of transfusion, median (IQR) | |

© 2022 Park H et al. *JAMA Network Open.*

| | |
|---|---|
| Lymphocyte count, x $10^9$/L (n = 215) | 0.8 (0.5 – 1.2) |
| C-reactive protein, mg/dL (n = 211) | 6.8 (3.2 – 12.2) |
| SARS-CoV-2 PCR Cycle threshold value, median (IQR) (n = 183) | 22.1 (18.6 – 26.1) |

[A]Body mass index (BMI) is the weight in kilograms divided by the square of the height in meters. [B] Information on race and ethnic group was obtained from entries in the medical record, as reported by the patients. IQR, interquartile range; NIV, noninvasive ventilation; PCR, polymerase chain reaction.

© 2022 Park H et al. *JAMA Network Open.*

## eTable 32. Summary of Balance for Matched Data for the EUA

| | Mean CCP Treated (n= 210) | Mean Control (n= 210) | Std. Mean Diff. |
|---|---|---|---|
| Age | 66.452 | 64.838 | 0.095 |
| Sex | 0.423 | 0.419 | 0.009 |
| Diabetes | 0.390 | 0.419 | -0.058 |
| Pulmonary disease | 0.161 | 0.138 | 0.064 |
| Cardiovascular disease | 0.247 | 0.452 | -0.474 |
| Blood type A | 0.304 | 0.371 | -0.144 |
| Blood type B | 0.166 | 0.157 | 0.025 |
| Blood type AB | 0.033 | 0.023 | 0.053 |
| Blood type O | 0.495 | 0.447 | 0.095 |
| Baseline WHO =4 | 0.490 | 0.152 | 0.676 |
| Baseline WHO =5 | 0.381 | 0.638 | -0.529 |
| Baseline WHO =6 | 0.129 | 0.209 | -0.241 |
| Enrollment quarter 4 | 0.129 | 0.290 | -0.483 |
| Enrollment quarter 5 | 0.871 | 0.710 | -0.483 |

© 2022 Park H et al. *JAMA Network Open.*

eTable 33. Patients in Different Benefit Levels defined by the Basic TBI and the Expanded TBI for the EUA

| | Benefit Group | Basic TBI | Expanded TBI |
|---|---|---|---|
| | | % of patients | % of patients |
| Two levels of benefit | B | 56% (n=237) | 59% (n=246) |
| | NB | 44% (n=183) | 41% (n=174) |
| Three levels of benefit | B1 | 39% (n=163) | 32% (n=133) |
| | B2 | 25% (n=107) | 37% (n=155) |
| | B3 | 36% (n=150) | 31% (n=132) |

© 2022 Park H et al. *JAMA Network Open.*

eTable 34. Subgroup-Specific CCP Benefit Odds Ratios for Benefit Levels Defined by the Basic TBI and the Expanded TBI for the EUA

| | Group | Basic TBI | Expanded TBI |
|---|---|---|---|
| | | OR (95% CI) | OR (95% CI) |
| Two levels of benefit | B | 0.87 (0.56, 1.37) | 0.80 (0.51, 1.24) |
| | NB | 2.66 (1.60, 4.42) | 3.24 (1.92, 5.45) |
| Three levels of benefit | B1 | 0.63 (0.35, 1.13) | 0.91 (0.50, 1.65) |
| | B2 | 2.34 (1.15, 4.78) | 1.17 (0.67, 2.04) |
| | B3 | 3.14 (1.78, 5.54) | 3.00 (1.64, 5.46) |

© 2022 Park H et al. *JAMA Network Open.*

## eAppendix 28. Validation on External RCT 1

Description of the validation RCT:

A single center open label phase II RCT was done to assess the pathogen and host-intrinsic factors influencing clinical and immunological benefits of passive immunization using CCP therapy, in addition to standard of care (SOC) therapy (clinical trial registration: Clinical Trial Registry of India No. CTRI/2020/05/025209)[24]. Convalescent plasma was collected from patients recovered from COVID-19 (with disease remission at least 28 days prior to screening and on attaining negative status on SARS-CoV-2 RT-PCR) following a screening protocol which also included measuring plasma anti SARS-CoV-2 spike IgG content. Severe COVID-19 patients with evidence for acute respiratory distress syndrome (ARDS) with PaO2/FiO2 ratio 100-300 (moderate ARDS) were recruited and randomized into two parallel arms of SOC and CCP, n=40 in each arm. See Table S.6.10 below for the patients' pretreatment characteristics and their clinical information. Patients were followed up for 30 days post-admission to assess the primary outcomes of all-cause mortality and immunological correlates for clinical benefits.

The standard of care received by the patients (which was added with two transfusions of 200ml CCP on two consecutive days for the CCP arm) included variable pharmacotherapy, in addition to standard protocols for O2 therapy as described in eTable 36 below.

Approach:

In this study, blood type information was only available in patients randomized to CCP and therefore, only the Basic TBI was able to be computed and used for the validation. The Basic TBI scores for all n=80 patients were computed, and these TBI scores were then used to stratify the whole (n=80) sample into 1) two groups: expected to benefit (B) and not expected to benefit (NB); and 2) three groups: large benefit (B1), moderate benefit (B2) and potential harm (B3) expected from the CCP treatment, using the cutoff points specified in eTable 14. Then we computed the subgroup-specific ORs to validate the relationship between the TBI and the CCP efficacy. Mortality at day 30 was the only available outcome in common to the COMPILE outcomes. We used logistic regression, with the treatment indicator as the only covariate, to analyze this outcome.

Results:

Based on the TBI scores, we identified that 20 and 60 belong to the subgroups B and NB, respectively, and that 16, 48 and 16 belong to the subgroups B1, B2 and B3 respectively, as reported in eTable 37 below.

The ORs (and 95% CIs) computed within the subgroups defined by the Basic TBI are reported in eTable 38 below, supporting validation of the TBI. The results of the primary analysis are highlighted.

Since the data are from an RCT, we can draw the relationship between the CCP efficacy odds ratio and the TBI, using the whole n=80 sample (as in Figure 1 in the main text), as displayed in eFigure 25 below.

Although the confidence bounds for the OR's are wide (a consequence of the small sample size), the Basic TBI is considered externally validated because specification for the subgroups were determined independently using the COMPILE data set and the respective ORs have the benefit relationships, OR(B) < OR(NB) and OR(B1) < OR(B2) < OR(B3), that were predicted by the TBI to exhibit on this external dataset. (We note that imputing blood type information and using the Expanded TBI produced results similar to those of the Basic TBI.)

[24] Ray Y, Paul SR, Bandopadhyay P, et al. Clinical and immunological benefits of convalescent plasma therapy in severe COVID-19: insights from a single center open label randomised control trial. *medRxiv.* 2020:2020.2011.2025.20237883.

© 2022 Park H et al. *JAMA Network Open.*

eTable 35. Baseline Patient Characteristics and Clinical Information of the First RCT

| | Control (n=40) | CCP (n=40) | Overall (n=80) |
|---|---|---|---|
| Age (median, IQR) | 65.00 [57.50, | 57.50 [51.75, | 61.50 [52.00, |
| Female sex (n, %) | | | |
| Male | 30 (75.0) | 27 (67.5) | 57(71.2) |
| Female | 10 (25.0) | 13 (32.5) | 23(28.8) |
| Baseline WHO severity (n, %) | | | |
| 4 - hospitalized/ no $O_2$ | 0(0.0) | 0(0.0) | 0(0.0) |
| 5 - hospitalized/ $O_2$ by mask or nasal | 1(2.5) | 3(7.5) | 4(5.0) |
| 6 - hospitalized/ $O_2$ by non-invasive | 39(97.5) | 37(92.5) | 76(95.0) |
| Blood group (n, %) | | | |
| O | | 12(30.0) | 12(15.0) |
| A | | 9(22.5) | 9(11.3) |
| B | | 17(42.5) | 17(21.3) |
| AB | | 2(5.0) | 2(2.5) |
| NA | 40(100.0) | 0(0.0) | 40(50.0) |
| History of diabetes (n, %) | | | |
| No | 23(57.5) | 22 (55.0) | 45(56.2) |
| Yes | 17(42.5) | 18 (45.0) | 35(43.8) |
| History of pulmonary disease (n, %) | | | |
| No | 37(92.5) | 38(95.0) | 75(93.8) |
| Yes | 3(7.5) | 2(5.0) | 5(6.2) |
| History of cardiovascular disease (n, %) | | | |
| No | 25(62.5) | 17(42.5) | 48 (60.0) |
| Yes | 15 (37.5) | 23 (57.5) | 32 (40.0) |
| Enrollment quarter (n, %) | | | |
| Apr-June 2020 | 6 (15.0) | 6 (15.0) | 12 (15.0) |
| July-Sept 2020 | 28 (70.0) | 26 (65) | 54 (67.5) |
| Oct-Dec 2020 | 6 (15.0) | 8 (20.0) | 14 (17.5) |
| Jan-Mar 2021 | 0 (0.0) | 0 (0.0) | 0 (0.0) |
| All-cause mortality at day 30 (n, %) | 14 (35.0) | 10 (25.0) | 24 (30.0) |

## eTable 36. Major Pharmacotherapy as the Standard of Care

| Major pharmacotherapy | Control (n=40) | CCP (n=40) |
|---|---|---|
| Hydroxycholoroquin | 12 (30%) | 19 (47.5%) |
| Ivermectin | 26 (65%) | 27(67.5%) |
| Doxycyclin | 21 (52.5%) | 25 (62.5%) |
| Azithromycin | 16 (40%) | 19 (47.5%) |
| Meropenem | 7 (17.5%) | 9 (22.5%) |
| Faropenem | 9 (22.5%) | 3 (7.5%) |
| Ceftriaxone | 4 (10%) | 4 (10%) |
| Remdesivir | 13 (32.5%) | 12 (30%) |
| Intravenous corticosteroid | 19 (47.5%) | 21 (52.5%) |
| Oral corticosteroid | 14 (35%) | 9 (22.5%) |
| Metformin | 8 (20%) | 11 (27.5%) |
| Insulin | 17 (42.5%) | 17 (42.5%) |
| Prophylactic low mol wt heparin | 24 (60%) | 28 (70%) |
| Therapeutic low mol wt heparin | 5 (12.5%) | 4 (10%) |

© 2022 Park H et al. *JAMA Network Open.*

eTable 37. Patients in Different Benefit Levels Based on the Basic TBI in the First RCT

| | Benefit Group | Basic TBI |
|---|---|---|
| | | % of patients |
| Two levels of benefit | B | 25% (n=20) |
| | NB | 75% (n=60) |
| Three levels of benefit | B1 | 20% (n=16) |
| | B2 | 60% (n=48) |
| | B3 | 20% (n=16) |

© 2022 Park H et al. *JAMA Network Open.*

eTable 38. Subgroup-Specific CCP Benefit Odds Ratios With Respect to 30-Day Mortality, by Benefit Level Defined by the Basic TBI, in the First RCT

| | Benefit Group | Basic TBI |
|---|---|---|
| | | OR (95% CI) |
| Two levels of benefit | B | 0.35 (0.03, 4.65) |
| | NB | 0.71 (0.24, 2.07) |
| Three levels of benefit | B1 | 0.31 (0.02, 4.41) |
| | B2 | 0.69 (0.21, 2.30) |
| | B3 | 0.80 (0.09, 6.85) |

© 2022 Park H et al. *JAMA Network Open.*

eFigure 25. CCP vs Control as a Function of the Basic TBI (Which Does Not Require Blood Type Information), With the 3 Benefit Groups Overlaid, Evaluated on the First External RCT

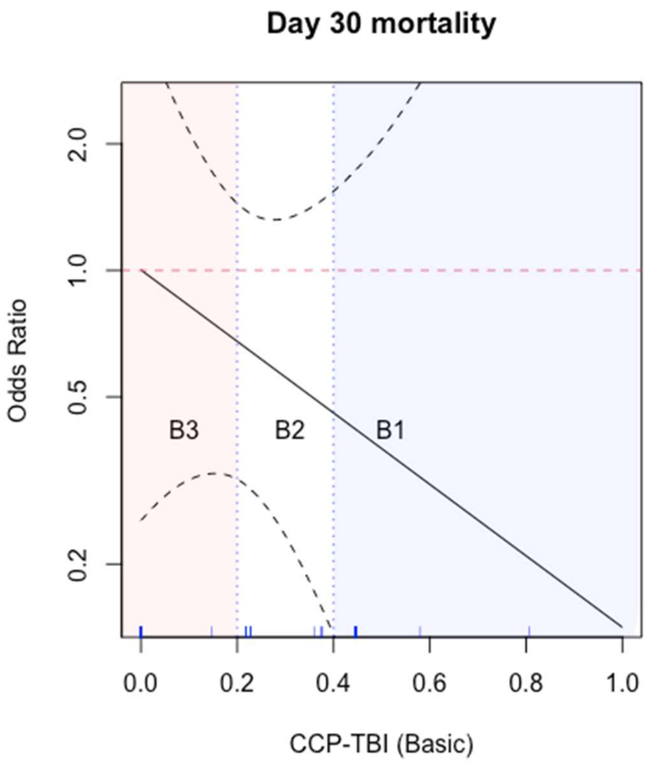


© 2022 Park H et al. *JAMA Network Open.*

## eAppendix 29. Validation on External RCT 2

Description of the validation RCT:

A total of 228 patients were assigned to receive convalescent plasma and 105 to receive placebo (PlasmAr ClinicalTrials.gov number, NCT04383535)[25]. The infused CCP had a median titer of 1:3200 of total SARS-CoV-2 antibodies (interquartile range, 1:800 to 1:3200]. No patients were lost to follow-up. The primary outcome of the study was clinical status 30 days after intervention, as represented by one of six mutually exclusive ordinal categories on an adapted version of the World Health Organization (WHO) clinical scale:
1: Not hospitalized without limitation in activity
2: Not hospitalized with limitation in activity
3: Hospitalized not on supplemental oxygen
4: Hospitalized on supplemental oxygen
5: Hospitalized on invasive mechanical ventilation
6: Death

At day 30 day, no significant difference was noted between the CCP group and the placebo group in the distribution of clinical outcomes according to the ordinal scale (odds ratio, 0.83 (95% confidence interval [CI], 0.52 to 1.35; p-value=0.46). Overall mortality was 10.96% in the convalescent plasma group and 11.43% in the placebo group, for a risk difference of -0.46 percentage points (95% CI, -7.8 to 6.8). See eTable 39 for the patients' pretreatment characteristics and clinical information.

Approach:

Both the Basic TBI and the Expanded TBI were considered for this external validation. The Basic and Expanded TBI scores for n=309 (we excluded n=24 patients with missing baseline oxygen supplementation status) patients were computed to stratify the sample into 1) two groups: expected to benefit (B) and not expected to benefit (NB); and 2) three groups: large benefit (B1), moderate benefit (B2) and potential harm (B3) expected from the CCP treatment, using the cutoff points specified in Table 1 in the main text. The ORs computed within these subgroups, evaluated with respect to the WHO 6-point scale ordinal outcome specified above, were compared one another to validate the relationship between the TBIs and the CCP efficacy. The cumulative ORs, computed for both the days 14 and 30 outcomes, were obtained based on cumulative logit proportional odds models with the treatment indicator as the only covariate.
Since the study does not have the baseline WHO scale, the information about oxygen supplementation and ICU at baseline were used to surrogate the baseline WHO 11-point scale (4, 5 and 6), using the following scheme:

1. No oxygen supplementation at baseline: WHO 11-point scale 4.
2. Oxygen supplementation but no ICU admission at baseline: WHO 11-point scale 5.
3. Oxygen supplementation and ICU admission at baseline: WHO 11-point scale 6.

Results:

eTable 40 below shows the proportion of patients in different benefit levels defined by the Basic and the Expanded TBIs.

eTable 41 and eTable 42 below report the corresponding ORs, for the days 14 and 30 WHO 6-point scale ordinal outcomes (see eTable 41), and for the days 14 and 30 binary outcomes of ventilation or worse (see eTable 42). The results of the primary analysis are highlighted. eFigure 26 (for the ordinal outcomes) and

[25] Simonovich VA, Burgos Partx LD, Scibona P, et al. A randomized trial of convalecent plasma in Covid-19 severe pneumonia. *The New England Journal of Medicine*. 202; 384:619-629.

© 2022 Park H et al. *JAMA Network Open.*

eFigure 27 (for the binary outcomes) display the smoothed odds ratios as continuous functions of the Basic and Extended TBIs, respectively.

In eFigure 26 and eFigure 27, for both the Basic and Expanded TBIs, there is scarcity in the evaluated TBI scores greater than 0.6 (the small blue ticks on the horizontal axis represent the values of the TBI scores). As a consequence, the confidence bounds for the associated ORs are wide for the TBI values greater than 0.6. This is because of the difference in the enrolled patient population between this RCT and the COMILE study. Nevertheless, they exhibit monotone decreasing relationships with the TBIs (i.e., a large value of the TBI designates a large benefit), indicating validation of the TBIs on this external RCT.

© 2022 Park H et al. *JAMA Network Open.*

## eTable 39. Baseline Patient Characteristics for the Second RCT

| | Control (n=105) | CCP (n=228) | Overall (n=333) |
|---|---|---|---|
| Age (median, IQR) | 62.00 [49.00, 71.00] | 62.08 [53.00, 72.25] | 62.00 [52.00, 72.00] |
| Sex (n, %) | | | |
| Male | 64 (60.9) | 161(70.6) | 225(67.6) |
| Female | 41(39.1) | 67(29.4) | 108(32.4) |
| Baseline oxygen supplementation (n, %) | | | |
| No | 12(11.4) | 20(8.8) | 32(9.6) |
| Yes | 86(81.9) | 191(83.8) | 277(83.1) |
| NA | 7(6.7) | 17(7.5) | 24(7.2) |
| ICU admission at baseline (n, %) | | | |
| No | 80(76.2) | 161(70.6) | 241(72.4) |
| Yes | 25(23.8) | 67(29.4) | 92(28.2) |
| Blood group (n, %) | | | |
| O | 44(42.0) | 112(49.0) | 156(47.0) |
| A | 46(44.0) | 87(38.0) | 133(40.0) |
| B | 12(11.0) | 16(7.0) | 28(8.0) |
| AB | 3(3.0) | 13(3.0) | 16(5.0) |
| History of diabetes (n, %) | | | |
| No | 84(80.0) | 180(82.5) | 272(81.7) |
| Yes | 21(20.0) | 40(17.5) | 61(18.3) |
| History of pulmonary disease (n, %) | | | |
| No | 98(93.3) | 198(86.8) | 296(88.9) |
| Yes | 7(6.7) | 30(13.2) | 37(11.1) |
| History of cardiovascular disease (n, %) | | | |
| No | 94(89.5) | 203(89.0) | 297(89.2) |
| Yes | 11(10.5) | 25(11.0) | 36(10.8) |
| Enrollment quarter (n, %) | | | |
| Apr-June 2020 | 13(12.4) | 25(11.0) | 38(11.4) |
| July-Sept 2020 | 92(87.6) | 203(89.0) | 295(88.6) |
| Oct-Dec 2020 | 0(0.0) | 0(0.0) | 0(0.0) |
| Jan-Mar 2021 | 0(0.0) | 0(0.0) | 0(0.0) |
| All-cause mortality at day 30 (n, %) | 12(11.4) | 25(11.0) | 37(11.1) |

© 2022 Park H et al. *JAMA Network Open.*

eTable 40. Patients in Different Benefit Levels Based on the Expanded and Basic TBIs for the Second RCT.

| | Benefit Group | Basic TBI | Expanded TBI |
|---|---|---|---|
| | | % of patients | % of patients |
| Two levels of benefit | B | 21% (n=65) | 27% (n=84) |
| | NB | 79% (n=244) | 73% (n=225) |
| Three levels of benefit | B1 | 14% (n=44) | 13% (n=40) |
| | B2 | 23% (n=70) | 39% (n=120) |
| | B3 | 63% (n=195) | 48% (n=149) |

© 2022 Park H et al. *JAMA Network Open.*

eTable 41. CCP Benefit Odds Ratios for Ordinal Outcomes at Days 14 and 30 for the Benefit Groups Defined by the Basic and Expanded TBIs, Evaluated on the Second External RCT

| | | Day 14 ordinal outcome | | Day 30 ordinal outcome | |
|---|---|---|---|---|---|
| | Group | Basic TBI | Expanded TBI | Basic TBI | Expanded TBI |
| | | OR (95% CI) | OR (95% CI) | OR (95% CI) | OR (95% CI) |
| Two levels of benefit | B | 0.47 (0.17, 1.29) | 0.69 (0.28, 1.69) | 0.53 (0.19, 1.46) | 0.71 (0.28, 1.82) |
| | NB | 1.08 (0.66, 1.77) | 1.02 (0.61, 1.70) | 1.43 (0.80, 2.53) | 1.35 (0.75, 2.42) |
| Three levels of benefit | B1 | 0.58 (0.17, 2.02) | 0.44 (0.12, 1.65) | 0.72 (0.20, 2.55) | 0.43 (0.12, 1.61) |
| | B2 | 0.85 (0.33, 2.22) | 0.99 (0.49, 2.01) | 1.16 (0.36, 3.73) | 1.42 (0.63, 3.21) |
| | B3 | 1.02 (0.59, 1.77) | 1.04 (0.55, 1.96) | 1.26 (0.68, 2.33) | 1.25 (0.61, 2.55) |

© 2022 Park H et al. *JAMA Network Open.*

eFigure 26. CCP Benefit Odds Ratios for WHO 6-Point Scale Ordinal Outcomes at Days 14 and 30, as Functions of the Basic and Expanded TBIs, Evaluated on the Second External RCT

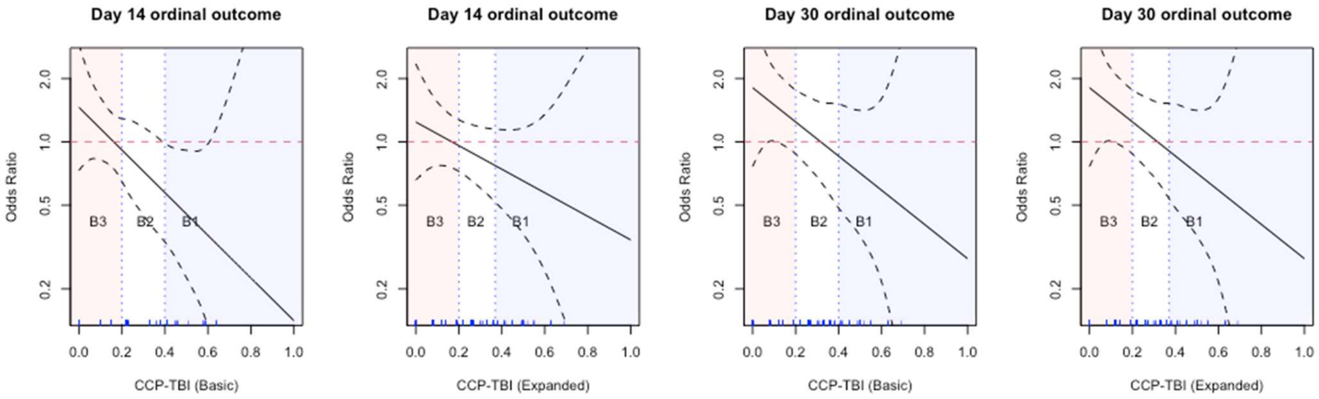


© 2022 Park H et al. *JAMA Network Open.*

eTable 42. CCP Benefit Odds Ratios for the Binary Outcome of Ventilation or Worse at Days 14 and 30 for Benefit Groups Defined by the Basic and Expanded TBIs, Evaluated on the Second External RCT

| | | Day 14 ventilation or worse | | Day 30 ventilation or worse | |
|---|---|---|---|---|---|
| | Group | Basic TBI | Expanded TBI | Basic TBI | Expanded TBI |
| | | OR (95% CI) | OR (95% CI) | OR (95% CI) | OR (95% CI) |
| Two levels of benefit | B | 0.23 (0.07, 0.80) | 0.40 (0.13, 1.19) | 0.40 (0.12, 1.33) | 0.49 (0.16, 1.48) |
| | NB | 1.04 (0.53, 2.01) | 0.94 (0.47, 1.85) | 1.09 (0.54, 2.19) | 1.06 (0.52, 2.15) |
| Three levels of benefit | B1 | 0.31 (0.07, 1.34) | 0.19 (0.04, 0.94) | 0.54 (0.12, 2.36) | 0.36 (0.08, 1.74) |
| | B2 | 0.75 (0.22, 2.56) | 0.70 (0.27, 1.81) | 0.65 (0.17, 2.53) | 0.74 (0.28, 1.98) |
| | B3 | 0.91 (0.44, 1.89) | 1.09 (0.47, 2.52) | 1.05 (0.50, 2.20) | 1.18 (0.50, 2.80) |

© 2022 Park H et al. *JAMA Network Open.*

eFigure 27. CCP Benefit Odds Ratios for Binary Outcome of Ventilation or Worse at Days 14 and 30 for Benefit Groups Defined by the Basic and Expanded TBIs, Evaluated on the Second External RCT

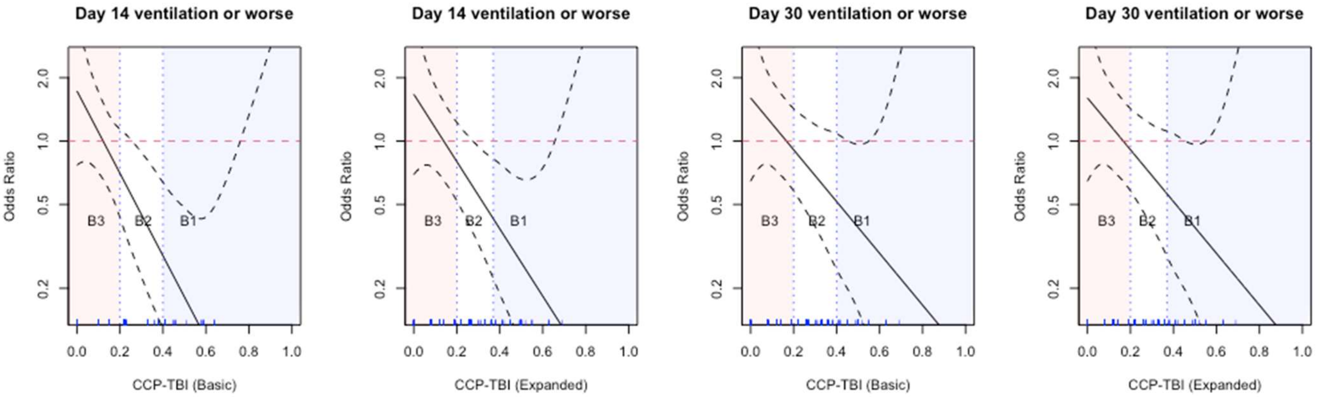


© 2022 Park H et al. *JAMA Network Open.*

## eAppendix 30. More Results From the Expanded TBI

In eFigure 28 below, we categorized the 11-point WHO ordinal outcome scales into 4 ordinal categories: i) WHO scale 0-3 (non-hospitalized); ii) WHO scale 4-6 (hospitalized but without mechanical ventilation); iii) WHO scale 7-9 (mechanically ventilated); iv) WHO scale 10 (death), and displayed the stacked probability of these 4 ordinal categories, as a function of the CCP-TBI and the treatment conditions (CCP or Control).

In the plots, the darkest (black) colored band corresponds to the binary outcome WHO score =10 (i.e., death). As the value of the CCP-TBI increases (from 0 to 1), the risk of a bad outcome (e.g., death) under the CCP treatment generally *decreases*, whereas the risk under the Control treatment generally *increases*, for the both outcomes at days 14 (in the left panel) and 28 (in the right panel).

When TBI ≤ 0.2, the heights of all color bands are similar for both treatments (CCP and Control) or the Control is slightly favored, indicating that such patients would not benefit much from the CCP treatment. On the other hand, when TBI ≥ 0.4, CCP-treated patients are expected to have a better outcome than control-treated. In other words, the patients with TBI ≥ 0.4 are predicted to experience a better outcome with CCP treatment, compared to what is predicted under the Control treatment (i.e., the height of the lighter-colored bands is greater for the CCP-treated than the Control-treated when TBI ≥ 0.4).

© 2022 Park H et al. *JAMA Network Open.*

## eFigure 28. Probability of the 11-Point WHO Ordinal Scales Categorized into 4 Ordinal Categories, as a Function of the Expanded TBI

The probability of each outcome category, predicted by the TBI ranging from 0 to 1, under the two treatment conditions (CCP and Control, respectively) is displayed. These stacked probability plots describe the relationship between the CCP-TBI and the treatment outcomes at day 14 (in the left panel) and day 28 (in the right panel).

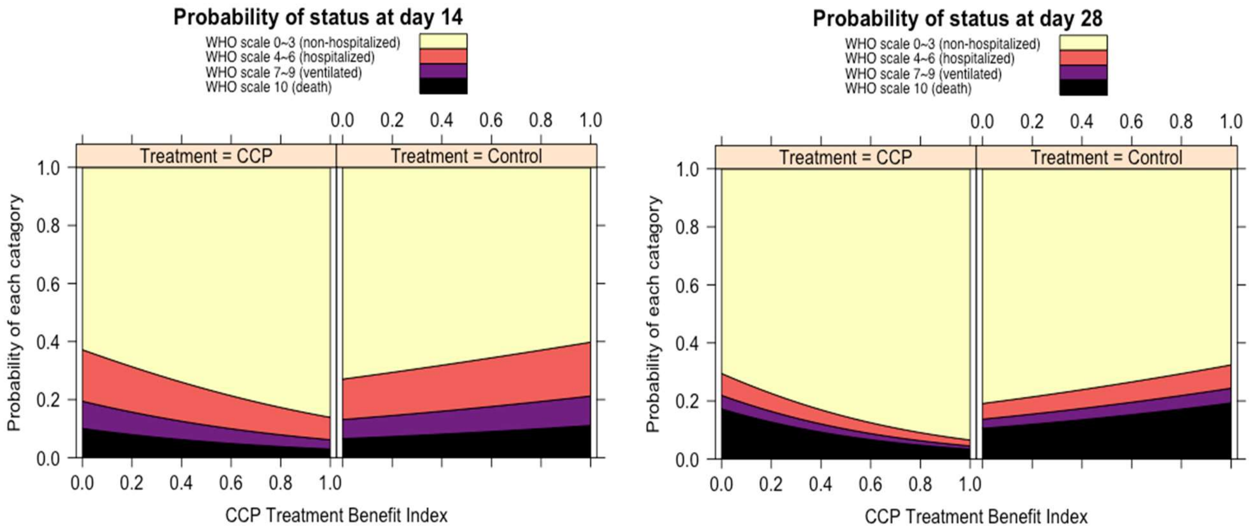


© 2022 Park H et al. *JAMA Network Open.*

## eAppendix 31. Response Surface and Scatterplots Associated With the Expanded TBI Model

We display the response surface of the fitted POM (S1.1 in eAppendix 1) with respect to the logit scale and the probability scale.

© 2022 Park H et al. *JAMA Network Open.*

## eFigure 29. Fitted POM

The left two panels: the cumulative ODDS($Y \geq y$) predicted by the cumulative logit POM (S1.1 in eAppendix 1) (for a fixed value of $y$), as a 2-dimensional response surface of the Risk index and the TBI, under the CCP treatment (left panel) and under the control treatment (middle panel), respectively. Risk Index refers to the term $\mu(\boldsymbol{Z})$ in POM (S1.1in eAppendix 1), that is scaled to [0,1]; see eTable 4 for the term $\mu(\boldsymbol{Z})$. TBI refers to the component $\boldsymbol{\beta}^{\boldsymbol{T}}\boldsymbol{X}$ in POM (S1.1 in eAppendix 1), that is scaled to [0,1].
The right panel: the cumulative odds ratio comparing CCP vs. control, implied by POM (S1.1), as a function of Risk Index and TBI. Notice that the odds ratio (OR) surface depends only on TBI (and not on Risk Index). This is because, in POM (S1.1 in eAppendix 1), the CCP efficacy OR is a function only of TBI, and not of Risk Index. Thus, independently of Risk Index, TBI can be used to evaluate the relative benefit of CCP vs. control.

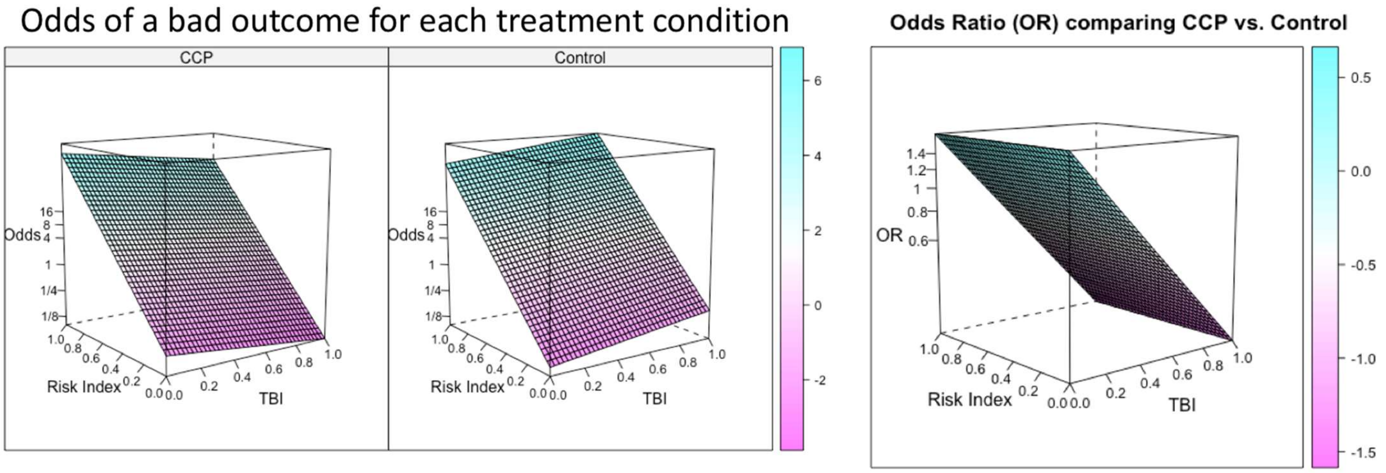


© 2022 Park H et al. *JAMA Network Open.*

# eFigure 30. Fitted POM Displayed on the Probability Scale

<u>The left two panels</u>: the fitted cumulative probability $P(Y \geq y)$ predicted by the cumulative logit POM (S1.1 in eAppendix 1) (for a fixed value of $y$), as a 2-dimensional response surface of the Risk index and TBI, for the CCP treatment (left panel) and the control treatment (right panel).
<u>The right panel</u>: the CCP-efficacy relative risk for a bad outcome (comparing CCP vs. control), predicted by the POM (S1.1 in eAppendix 1), as a function of the Risk Index and TBI.

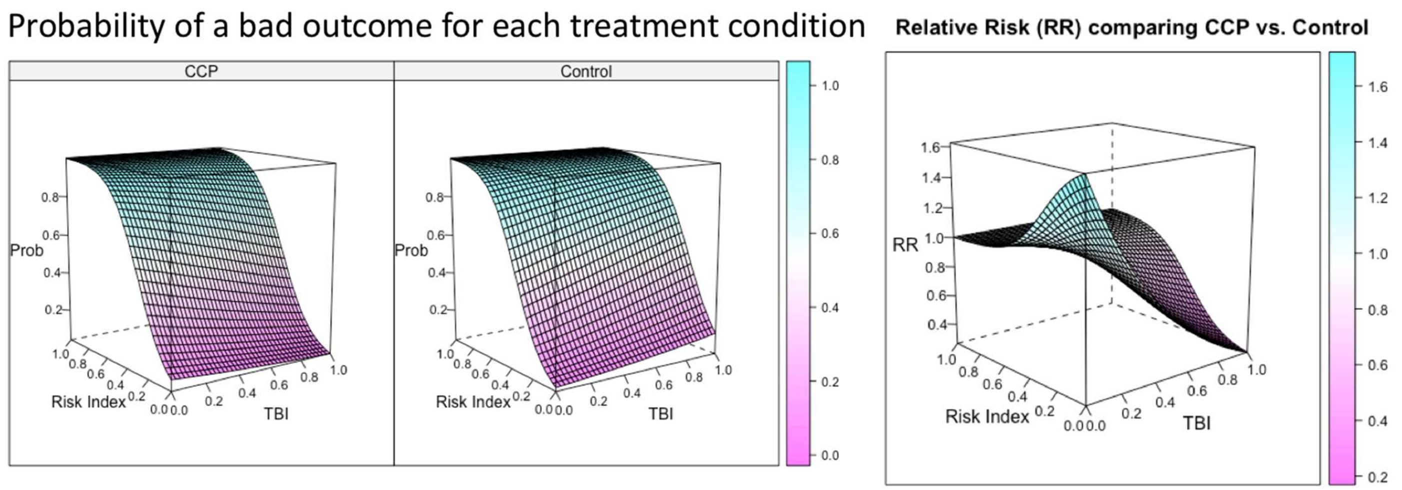


© 2022 Park H et al. *JAMA Network Open.*

# eFigure 31. Scatterplot of the Risk Index and the TBI in the Fitted POM

The Pearson correlation coefficient is -0.08, indicating only small correlation between the Risk Index and TBI. Unlike the Risk Index, TBI, by design, has a stronger interaction with treatment than any risk score.

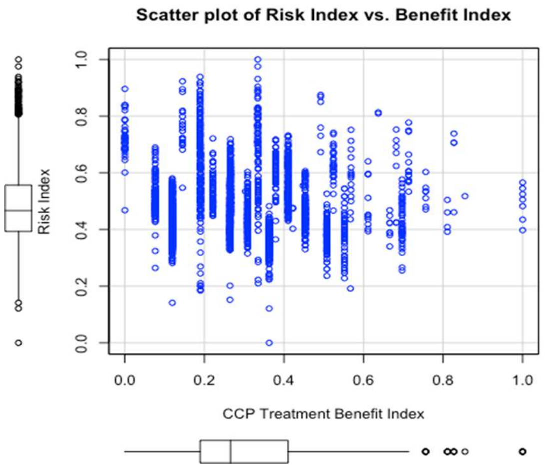


© 2022 Park H et al. *JAMA Network Open.*

# eFigure 32. Scatterplot of the Risk Index and the TBI-by-Treatment Interaction Term in the Fitted POM

By design, the Pearson correlation coefficient between these two terms is essentially 0, due to the constraint $E[g(\boldsymbol{\beta}^T \boldsymbol{X}, A)\,|\boldsymbol{X}] = 0$ that we imposed in the model (S1.1 in eAppendix 1).

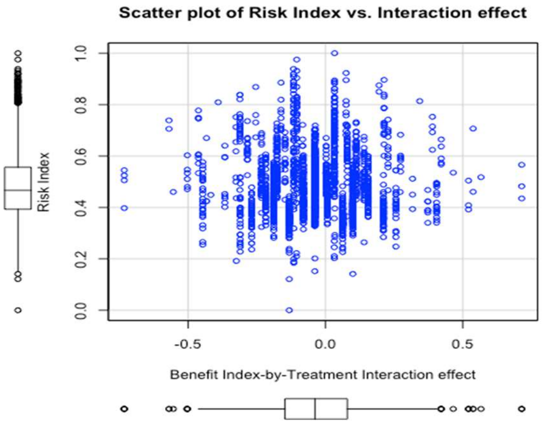


© 2022 Park H et al. *JAMA Network Open.*

## eFigure 33. Empirical Cumulative Distribution Functions for the Basic and Expanded TBIs With the Associated Benefit Levels Overlaid

The distribution of the basic TBI is coarser than that of the expanded TBI: the expanded TBI provides a finer gradation than the basic TBI.

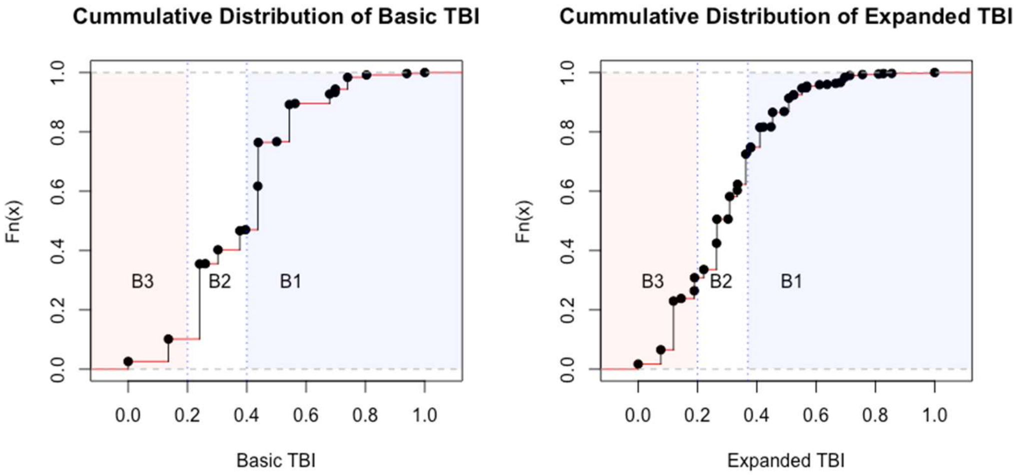


© 2022 Park H et al. *JAMA Network Open.*
